# Dysadherin/YAP axis fuels stem plasticity and immune escape in liver cancer

**DOI:** 10.1038/s41392-025-02520-4

**Published:** 2025-12-29

**Authors:** Tae-Young Jang, So-El Jeon, Hyeon-Ji Yun, Choong-Jae Lee, Da-Ye Lim, Sang Hoon Lee, Dajun Lee, Seungwon Lee, Jungmin Choi, Hyung-Sik Kim, Jeong-Seok Nam

**Affiliations:** 1 Department of Life Sciences, Gwangju Institute of Science and Technology, Gwangju, Republic of Korea; 2 Geninus Inc., Seoul, Republic of Korea; 3 Department of Biomedical Sciences, Korea University College of Medicine, Seoul, Republic of Korea; 4 Department of Oral Biochemistry, Dental and Life Science Institute, School of Dentistry, Pusan National University, Yangsan, Republic of Korea

**Keywords:** Cancer stem cells, Cancer therapy, Tumour biomarkers

## Abstract

Hepatocellular carcinoma (HCC) is an aggressive malignancy that is often refractory to chemotherapy and immune checkpoint inhibitors. This therapeutic resistance is driven in part by the persistence of cancer stem-like cells (CSCs) and the development of an immune-cold tumor microenvironment. However, the upstream regulators that coordinate these malignant features remain poorly defined. In this study, we identified dysadherin as a novel upstream activator of YAP that promotes both CSC plasticity and immune evasion through the FAK/YAP/TEAD2 signaling axis. Using single-cell transcriptomic analysis, in vitro assays, and multiple in vivo models including a humanized immune mouse system, we showed that dysadherin enhances the expression of pluripotency genes, such as *OCT4* and upregulates PD-L1. These changes support stem-like tumor behavior and contribute to T-cell exclusion, fostering an immunosuppressive niche. Notably, genetic knockdown or peptide-based pharmacologic inhibition of dysadherin effectively restored antitumor immune activation, suppressed metastasis and improved therapeutic responsiveness. Our findings reveal a mechanistic link between dysadherin-mediated cell adhesion signaling and the transcriptional regulation of both stemness and immune escape. Collectively, these findings establish the dysadherin/YAP axis as a key driver of HCC progression and resistance, and highlight it as a compelling therapeutic target that could overcome treatment failure in advanced liver cancer.

## Introduction

Hepatocellular carcinoma (HCC) is a highly heterogeneous malignancy that exhibits substantial variability both within individual tumors and across patients.^1,2^ This heterogeneity complicates treatment, driving therapeutic resistance and increasing the risks of metastasis and recurrence.^3^ A key factor in this complexity is the presence of cancer stem-like cells (CSCs) within HCC tumors.^4^ CSCs are characterized by their increased aggressiveness, therapy resistance, and a strong propensity for recurrence, all of which worsen clinical outcomes and hinder treatment efficacy.^5^ However, efforts to effectively target these cells have been hampered by an incomplete understanding of the molecular mechanisms that drive CSC phenotypes in HCC. Elucidating these mechanisms is critical for identifying actionable signaling pathways to overcome drug resistance and improve therapeutic outcomes.

Dysadherin is a glycoprotein found on the cell membrane that is associated with cancer progression.^6,7^ Previous clinical reports have described that elevated dysadherin expression is common in several human cancer cells but rare in normal cells.^8–11^ Elevated expression of dysadherin has been linked to tumor growth and metastasis and is associated with poor prognosis.^12,13^ In our previous study, we demonstrated that dysadherin-high cells exhibit CSC features and promote HCC progression.^14^ We also showed that dysadherin overexpression or silencing modulates resistance to doxorubicin-induced apoptosis in liver cancer cells.^14^ Despite these findings, the precise mechanisms by which dysadherin regulates CSC traits in HCC remain unclear. Unraveling these mechanisms may uncover novel therapeutic targets to combat drug resistance, metastasis, and recurrence in HCC.

Yes-associated protein (YAP), a key effector in the Hippo pathway, plays a central role in regulating cell growth and apoptosis.^15^ Under normal conditions, the Hippo signaling pathway tightly limits YAP activity to prevent uncontrolled cell proliferation, thereby maintaining tissue development and homeostasis.^16^ In HCC, however, dysregulation of this pathway leads to aberrant activation of YAP, which drives tumor growth and enhances CSC properties.^17–20^ Once activated, YAP interacts with TEA domain (TEAD) transcription factors and induces the expression of genes associated with tumor progression and drug resistance.^20–23^ Accumulating evidence also indicates that YAP facilitates immune evasion by enabling cancer cells to escape immune surveillance, ultimately conferring resistance to immune checkpoint inhibitors.^24–26^ Given its central role in HCC pathogenesis, YAP has emerged as a compelling therapeutic target, prompting efforts to develop direct inhibitors and combination strategies to counter its oncogenic functions. However, effectively targeting YAP remains challenging because of its complex upstream regulatory network and context-dependent activation. These limitations highlight the importance of identifying critical upstream regulators of YAP in HCC, which may offer more precise and effective avenues for therapeutic intervention.

This study provides evidence that the dysadherin/YAP axis enhances cancer stemness and promotes immune evasion in HCC. Elevated dysadherin expression is correlated with increased YAP transcriptional activity and the enrichment of cancer stem-like traits in both HCC mouse models and patient samples. Furthermore, dysadherin- and YAP-dependent upregulation of programmed death-ligand 1 (PD-L1) contributes to the development of an immunosuppressive tumor microenvironment. Our findings provide mechanistic insight into how dysadherin drives HCC progression and highlight its potential as a therapeutic target for disrupting YAP-mediated stemness and immune escape.

## Results

### Dysadherin promotes cancer stemness and tumor progression in HCC

**Fig. 1:**
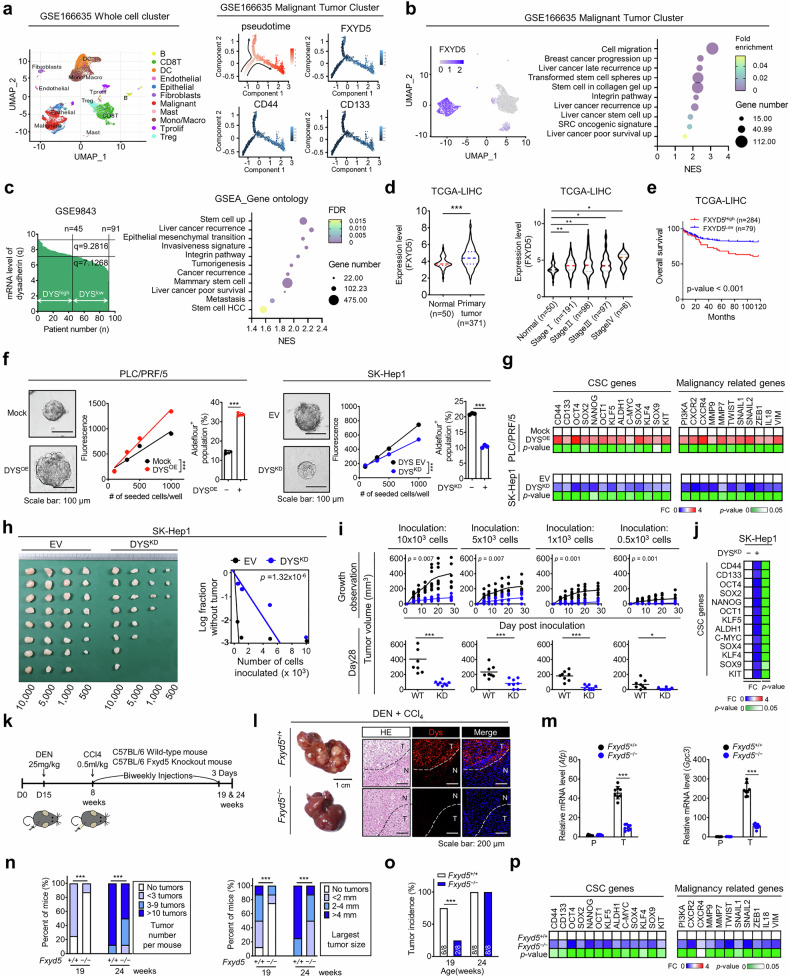
Dysadherin promotes cancer stem-like cell features and tumor progression in HCC. **a** UMAP plot of single-cell transcriptomes from HCC patient samples (GSE166635; *n* = 2). Cell lineage trajectories were inferred using Monocle2, highlighting the progression toward a malignant state. The expression of CSC- and malignancy-associated genes was mapped along the trajectory. **b** UMAP and GSEA of tumor clusters from GSE166635, comparing dysadherin^high^ (*n* = 1647) and dysadherin^low^ (*n* = 2853) tumor cells. **c** GSEA of DEGs between dysadherin^high^ and dysadherin^low^ tumors from bulk RNA-seq data (GSE9843; FDR < 0.05). **d** Violin plots showing *FXYD5* expression levels in normal liver tissue (*n* = 50) and primary HCC tissues (*n* = 371; TCGA-LIHC), and stratified by tumor grades. **e** Kaplan–Meier survival analysis for overall survival based on *FXYD5* expression in TCGA-LIHC cohort. Statistical significance was assessed by log-rank tests. **f** Limiting dilution sphere formation assay (left) and Aldefluor assay (right) evaluating the effect of dysadherin expression on CSC properties. Scale bar = 50 μm. **g** Heatmaps of CSC- and malignancy-related gene expression in HCC cells with dysadherin overexpression or knockdown. **h** In vivo limiting dilution assay (LDA) assessing the tumor-initiating potential of SK-Hep1 cells with or without dysadherin-knockdown (DYS^KD^). **i** Tumor growth curves from the in vivo limiting dilution assay comparing tumor-initiating capacity of DYS^KD^ versus control (WT) SK-Hep1 cells across multiple inoculation doses (0.5–10 × 10^3^ cells/mouse). **j** Heatmaps showing the expression of CSC-associated genes in dysadherin^KD^ versus control SK-Hep1 cells. **k** Schematic view of DEN/CCl_4_ -induced HCC mouse model. **l** Representative gross liver images and immunofluorescence staining of liver sections from *Fxyd5*^+/+^ and *Fxyd5*^−/−^ mice. Scale bar = 200 μm. **m** Expression of HCC markers in tumor (T) and paired adjacent normal tissues (P) from DEN/CCl_4_-treated mice. **n** Quantification of tumor number and distribution of tumor sizes, including largest tumor per mouse. **o** Tumor incidence in mice at the indicated time points. **p** Heatmap showing expression levels of CSC- and malignancy-associated genes in liver tissues from mice. Data are presented as means ± SEM. Statistical significance was determined by unpaired two-tailed Student’s t-tests and one-way ANOVA with Dunnett’s multiple comparison tests. *, ** and *** indicate p < 0.05, p < 0.01, and p < 0.001

To investigate the role of dysadherin in HCC progression, we analyzed single-cell RNA-sequencing (scRNA-seq) data from HCC patients (GSE166635) (Fig. 1a). Transcriptomic clustering revealed that *FXYD5* (dysadherin) expression was markedly increased in epithelial tumor clusters compared with normal epithelial cells (Fig. 1a and Supplementary Fig. 1a). Immunoblotting of paired patient tissues confirmed greater dysadherin protein expression in tumors than in adjacent non-tumor samples (Supplementary Fig. 1b). Pseudotime trajectory analysis reconstructed the progression of tumors toward an advanced malignant state. Along this trajectory, *FXYD5* expression progressively increased and became highly enriched in advanced tumors. This pattern coincided with the upregulation of key markers associated with cancer stemness (CD133, CD44) and metastasis/invasion (MMP7, CD74) (Fig. 1a and Supplementary Fig. 1c, d), highlighting *FXYD5* as a potential driver of malignant progression. To further define dysadherin-associated transcriptional programs, tumor cells were stratified into *FXYD5*^high^ and *FXYD5*^low^ groups for gene set enrichment analysis (GSEA). *FXYD5*^high^ tumor cells were enriched for gene signatures related to cancer stemness, metastasis, and poor prognosis (Fig. 1b and Supplementary Fig. 1c), which was validated in independent datasets (GSE9843 and GSE54236) (Fig. 1c and Supplementary Fig. 1e). Analysis of The Cancer Genome Atlas Liver Hepatocellular Carcinoma (TCGA-LIHC, v32) dataset further confirmed elevated *FXYD5* expression across tumor stages, and its association with reduced overall survival (Fig. 1d, e). A stratified survival analysis further revealed that while *FXYD5* expression had no significant prognostic value in early-stage (I/II) HCC, high *FXYD5* expression was significantly correlated with poor overall survival in late-stage (III/IV) patients (Supplementary Fig. 1f).

Functionally, dysadherin-overexpressing (OE) PLC/PRF/5 cells presented increased sphere formation and aldehyde dehydrogenase-positive (ALDH⁺) CSC populations, whereas knockdown (KD) of SK-Hep1 cells had the opposite effect (Fig. 1f and Supplementary Fig. 1g, h). Dysadherin overexpression also upregulated the expression of CSC- and malignancy-related genes, whereas its knockdown suppressed their expression (Fig. 1g). For in vivo validation, we determined the tumor-initiating capacity of SK-Hep1 cells using a limiting dilution assay. Dysadherin knockdown markedly suppressed the frequency of tumor formation (Fig. 1h, i) and downregulated the expression of CSC-associated genes (Fig. 1j).

In a diethylnitrosamine (DEN) / carbon tetrachloride (CCl₄)-induced HCC mouse model, *Fxyd5*-knockout (KO) mice (*Fxyd*5^−/−^) developed fewer and smaller tumors, with significantly lower tumor incidence at 19 weeks, than wild-type controls did (Fig. 1k–o and Supplementary Fig. 1i–k). Tumors from knockout mice also presented reduced expression of CSC and malignancy markers (Fig. 1p). Collectively, these findings demonstrate that dysadherin promotes HCC progression by enhancing cancer stemness and tumor aggressiveness, supporting its potential as both a therapeutic target and a prognostic biomarker.

### Dysadherin acts as an upstream regulator of YAP signaling to promote malignancy in HCC

**Fig. 2:**
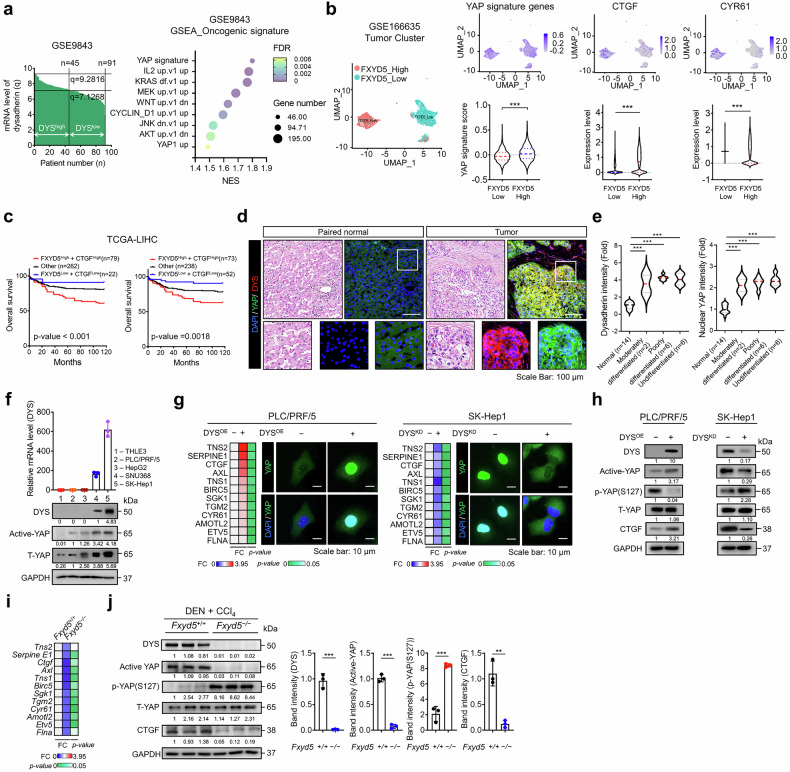
Dysadherin acts as an upstream regulator of YAP signaling to promote malignancy in HCC. **a** GSEA of DEGs between dysadherin^high^ and dysadherin^low^ HCC tumors (GSE9843), highlighting enrichment of YAP-related oncogenic signatures. **b** Left: UMAP plot of tumor cell clusters from GSE166635, color-coded by *FXYD5* expression. Right: UMAPs and violin plots showing YAP signature scores and expression of YAP target genes (*CTGF*, *CYR61*) in *FXYD5*^high^ versus *FXYD5*^low^ clusters. **c** Kaplan–Meier survival analysis of overall survival in TCGA-LIHC cohort stratified by high or low expression of *FXYD5* and YAP target genes (*CTGF* or *CYR61*). Significance was assessed by log-rank test. **d** Immunofluorescence (IF) staining of dysadherin and nuclear YAP in paired normal and tumor tissues from HCC patients (*n* = 14). Representative images and quantification of nuclear YAP signal intensity are shown. Scale bar = 100 μm. **e** Violin plots comparing dysadherin expression and nuclear YAP intensity across histological grades of HCC in patient tissues (*n* = 14). **f** RT-qPCR and immunoblot analyses of dysadherin, total YAP, and active YAP expression in normal hepatic (THLE-3) and HCC cell lines. **g** Heatmaps and immunofluorescence staining showing expression of YAP target genes and subcellular localization of active YAP in dysadherin-overexpressing (OE) and knockdown (KD) cells. Scale bar = 10 μm. **h** Immunoblotting analysis of active YAP (non-phosphorylated) and phospho-YAP (S127) following dysadherin OE or KD. **i** Heatmap showing YAP target gene expression in tumor from DEN/CCl₄-treated *Fxyd5*^+/+^ and *Fxyd5*^−/−^ mice. **j** Immunoblotting of YAP pathway components and downstream targets (CTGF) in liver lysates from *Fxyd5*^+/+^ and *Fxyd5*^−/−^ mice. Data are presented as means ± SEM. Statistical significance was determined by unpaired two-tailed Student’s t-tests and one-way ANOVA with Dunnett’s multiple comparison tests. *p < 0.05, **p < 0.01, ***p < 0.001

To investigate the mechanistic link between dysadherin and malignancy in HCC, we analyzed two independent patient-derived bulk RNA datasets (GSE9843 and GSE54236). GSEA between *FXYD5*^high^ and *FXYD5*^low^ tumors revealed significant enrichment of *YAP* transcriptional targets, with the conserved YAP signature ranking among the top oncogenic pathways (false discovery rate (FDR) < 0.05; Fig. 2a and Supplementary Fig. 2a). This finding was supported by strong positive correlations between *FXYD5* expression and the expression of canonical target genes of YAP, including *CTGF* and *CYR61*, in both datasets (Supplementary Fig. 2b). scRNA-seq analysis of primary HCC tumors (GSE166635) confirmed elevated YAP signature gene expression in *FXYD5*^high^ tumor clusters (Fig. 2b). TCGA-LIHC survival analysis further revealed that coexpression of *FXYD5* with *CTGF* or *CYR61* was significantly predictive of poor overall survival (p < 0.01; Fig. 2c), supporting the clinical relevance of the dysadherin/YAP axis.

Compared with those in matched normal tissue samples, histological and immunofluorescence (IF) analyses showed elevated dysadherin expression and augmented YAP nuclear translocation and activity in tumor samples (Fig. 2d and Supplementary Fig. 2c, d). Quantitative analysis of these patient samples further confirmed this finding, revealing a significant increase in nuclear YAP intensity in dysadherin^high^ tumors compared to their dysadherin^low^ counterparts (Supplementary Fig. 2d). These markers were highest in poorly differentiated or undifferentiated tumors and were correlated with advanced histological grade (Fig. 2e). Immunoblotting showed low levels of dysadherin and YAP in normal hepatic epithelial cells (THLE-3), but elevated total and active YAP in HCC cell lines (Fig. 2f). Dysadherin OE in PLC/PRF/5 cells increased the expression of nuclear YAP and its target genes, whereas dysadherin KD in SK-Hep1 cells reduced both (Fig. 2g and Supplementary Fig. 2e). Because YAP activation is regulated by Ser127 phosphorylation,^27,28^ we examined whether dysadherin affects this modification. Dysadherin OE reduced Ser127 phosphorylation and increased active YAP, whereas KD had the opposite effects (Fig. 2h). We confirmed these findings in another HCC cell line, SNU-368, in which dysadherin knockdown similarly impaired CSC properties (Supplementary Fig. 2f, g) and suppressed the YAP signaling pathway by preventing its nuclear accumulation and promoting its inhibitory phosphorylation (Supplementary Fig. 2h, i).

Our previous work demonstrated that dysadherin activates YAP by inducing integrin clustering and subsequent FAK activation.^29^ To confirm whether disrupting this axis could block YAP signaling in our model, we utilized a synthetic peptide previously designed to competitively inhibit the dysadherin-integrin interaction.^29^ Treatment with a dysadherin-targeting peptide reduced nuclear YAP levels, decreased the CSC population as indicated by ALDH activity, and downregulated the expression of CSC-associated, malignancy-related, and YAP target genes in SK-Hep1 cells (Supplementary Fig. 2j–m). These changes were confirmed by immunoblotting (Supplementary Fig. 2n).

Finally, in DEN/CCl₄-induced HCC mouse model, tumors from *Fxyd5*^−/−^ mice showed lower expression of YAP target genes and reduced nuclear YAP compared to wild-type controls (Fig. 2i, j). Taken together, these findings identify dysadherin as an upstream regulator of YAP signaling in HCC. Dysadherin enhances nuclear YAP accumulation by suppressing Ser127 phosphorylation, thereby promoting pro-malignant gene expression, cancer stemness, and tumor progression.

### Dysadherin promotes cancer stem features via YAP activation

**Fig. 3:**
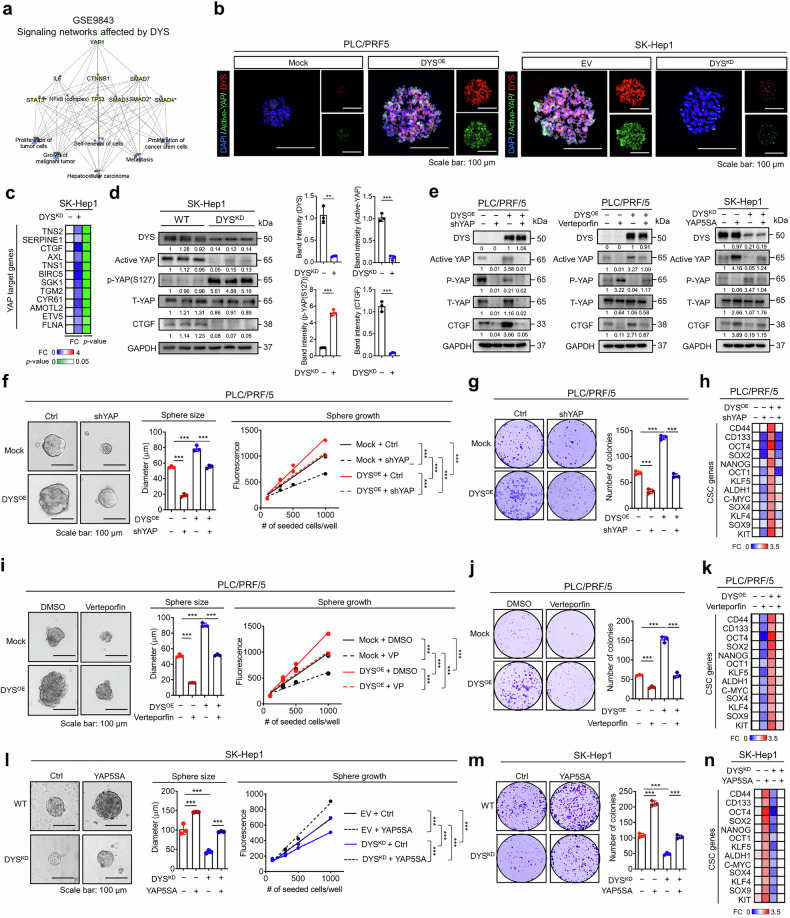
Dysadherin promotes CSC features through YAP activation in HCC. **a** Upstream regulator analysis using dysadherin-correlated transcriptional signatures in HCC tumors (GSE9843), predicting YAP as a central effector. **b** Immunofluorescence (IF) staining showing active YAP localization in spheroid cultures of dysadherin-overexpressing (OE) or knockdown (KD) HCC cells (PLC/PRF/5 and SK-Hep1). Scale bar = 100 μm. **c** Heatmaps showing the expression of YAP target genes in tumors derived from the LDA experiment. **d** Immunoblot analysis of active and total YAP, phospho-YAP (S127), and CTGF expression in tumors derived from the LDA experiment. **e** Immunoblot analysis showing YAP activation status in dysadherin OE or KD cells treated with YAP knockdown (shYAP), verteporfin (VP), or constitutively active YAP (YAP5SA). **f**, **i**, **l** Sphere formation assays showing size and growth of spheroids under the indicated conditions (shYAP, verteporfin, or YAP5SA). Scale bar = 100 μm. **g**, **j**, **m** Clonogenic survival assays measuring colony-forming ability in PLC/PRF/5 or SK-Hep1 cells. **h**, **k**, **n** Heatmaps showing expression of CSC-associated genes in dysadherin OE or KD cells. Data are presented as means ± SEM. Statistical significance was determined by unpaired two-tailed Student’s t-tests and one-way ANOVA with Dunnett’s multiple comparison tests. *p < 0.05, **p < 0.01, ***p < 0.001

To elucidate how dysadherin enhances cancer stemness and contributes to HCC malignancy, we performed upstream regulator analysis using DEGs from dysadherin^high^ tumors (GSE9843). Ingenuity pathway analysis (IPA) revealed that YAP signaling is a central node regulating self-renewal and malignancy-associated transcriptional programs (Fig. 3a), which is consistent with earlier findings of dysadherin-dependent YAP activation. To assess whether dysadherin modulates YAP activity in CSCs, we used 3D sphere cultures to enrich for stem-like cells. IF analysis showed that dysadherin OE in PLC/PRF/5 cells increased sphere size and active YAP levels, whereas dysadherin KD in SK-Hep1 and SNU-368 cells significantly reduced both (Fig. 3b and Supplementary Fig. 3a). Treatment with a dysadherin-targeting peptide phenocopied the KD effects, reducing sphere size and YAP activity (Supplementary Fig. 3b). Molecular analysis of tumors harvested from the limiting dilution assay (Fig. 1h) confirmed that dysadherin knockdown suppressed YAP activity (Fig. 3c, d).

To confirm that dysadherin enhances CSC traits via YAP signaling, we inhibited YAP genetically and pharmacologically. YAP silencing in dysadherin-OE PLC/PRF/5 cells reduced YAP activation (Fig. 3e and Supplementary Fig. 3c, d) and impaired CSC properties, including sphere formation, aldefluor activity, and colony growth (Fig. 3f, g and Supplementary Fig. 3f). Consistently, dysadherin-induced CSC gene expression was diminished. (Fig. 3h). Verteporfin, a YAP–TEAD inhibitor, similarly decreased YAP activity and suppressed CSC features and clonogenicity (Fig. 3e, i–k and Supplementary Fig. 3e, g). Conversely, the expression of constitutively active YAP (YAP5SA) in dysadherin-KD SK-Hep1 cells restored YAP activity, as did sphere and colony formation, as well as CSC gene expression (Fig. 3e, l–n and Supplementary Fig. 3h). These findings demonstrate that dysadherin drives HCC stemness through YAP activation, promoting YAP nuclear translocation and transcriptional activity to enhance self-renewal and tumor aggressiveness.

### Dysadherin upregulates pluripotent gene expression via the FAK/YAP/TEAD2 axis

**Fig. 4:**
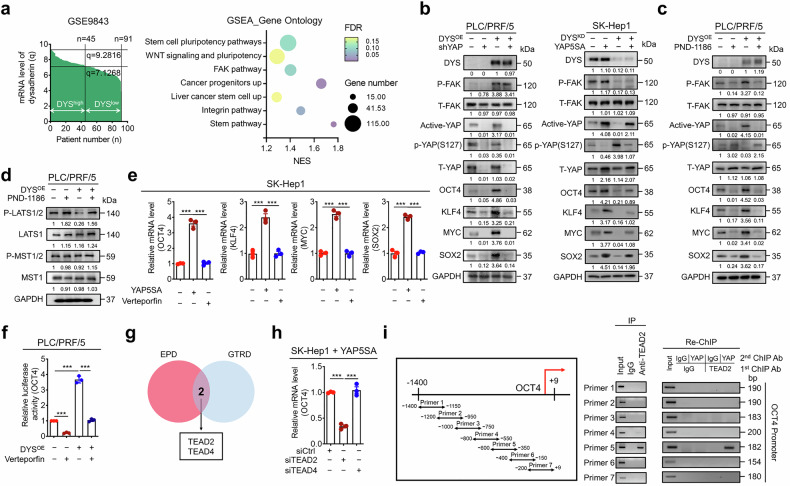
The dysadherin–FAK–YAP axis drives TEAD2-dependent transcription of pluripotency genes in HCC. **a** GSEA of DEGs from dysadherin^high^ versus dysadherin^low^ HCC tumors (GSE9843). **b**, **c** Immunoblot analyses showing levels of phosphorylated FAK (p-FAK), total and active YAP, phospho-YAP (S127), and pluripotency transcription factors (OCT4, KLF4, MYC, SOX2) in PLC/PRF/5 and SK-Hep1 cells. Cells were subjected to dysadherin OE or KD and treated with shYAP, constitutively active YAP mutant (YAP5SA), or the FAK inhibitor PND-1186. **d** Immunoblot analysis showing levels of dysadherin (DYS), phosphorylated FAK (P-FAK), total FAK (T-FAK), phosphorylated LATS1/2 (P-LATS1/2), total LATS1, phosphorylated MST1/2 (P-MST1/2), and total MST1 in PLC/PRF/5 cells. Cells were subjected to dysadherin overexpression with or without a FAK inhibitor. **e** RT-qPCR analysis of pluripotency genes in SK-Hep1 cells expressing YAP5SA or treated with verteporfin. **f** Luciferase reporter assay assessing *OCT4* promoter activity in dysadherin^OE^ PLC/PRF/5 cells treated with or without verteporfin. **g** Venn diagram illustrating overlap of TEAD2 and TEAD4 as candidate transcriptional regulators of OCT4 from the EPD and GTRD databases. **h** RT-qPCR analysis of *OCT4* mRNA levels in YAP5SA-expressing SK-Hep1 cells following siRNA-mediated knockdown of TEAD2 or TEAD4. **i** ChIP-qPCR confirming direct binding of TEAD2 to the OCT4 promoter region using seven primer sets spanning from −1400 to +9 bp relative to the transcription start site. For ChIP-Re-ChIP analysis in SK-Hep1 cells, chromatin was first immunoprecipitated with an anti-TEAD2 antibody, and the resulting material was re-immunoprecipitated with an anti-YAP antibody. PCR was performed with primers spanning the OCT4 promoter. IgG was used as a negative control. Data are presented as means ± SEM. Statistical significance was determined by unpaired two-tailed Student’s t-tests or one-way ANOVA with Dunnett’s multiple comparison test. *p < 0.05, **p < 0.01, ***p < 0.001

Given that focal adhesion kinase (FAK) acts upstream of YAP and regulates cancer stemness in multiple tumor contexts, and that dysadherin is known to activate integrin–FAK signaling,^29–31^ we explored whether the dysadherin–FAK–YAP axis drives the transcription of pluripotency genes in HCC. Gene set enrichment and upstream regulator analyses (GSEA and IPA) of dysadherin^high^ HCC samples revealed strong enrichment of FAK activation, YAP signaling, and stem cell pluripotency pathways (Fig. 4a and Supplementary Fig. 4a). We first sought to confirm the physical association of dysadherin with the integrin-FAK pathway in our HCC model. Through coimmunoprecipitation, we confirmed a clear interaction between endogenous dysadherin and fibronectin (Supplementary Fig. 4b). We assessed the expression of core pluripotency factors (*OCT4*, *KLF4*, *MYC*, and *SOX2*) in PLC/PRF/5, SK-Hep1, and SNU-368 cells. Dysadherin OE increased phosphorylated FAK (p-FAK), activated YAP, and upregulated pluripotency markers, whereas YAP inhibition via shRNA or verteporfin reversed these effects (Fig. 4b and Supplementary Fig. 4c). Dysadherin KD suppressed YAP activity and pluripotency gene expression, which was partially rescued by YAP5SA expression (Fig. 4b). To confirm the role of integrin–FAK signaling, we used the FAK inhibitor, PND-1186, and the integrin inhibitor, MK-0429. Both inhibitors specifically suppressed the dysadherin-induced increase in FAK phosphorylation, YAP activation, and pluripotency gene expression, supporting a role for integrin–FAK-YAP signaling in this transcriptional cascade (Fig. 4c and Supplementary Fig. 4d, e). To further elucidate how the FAK-YAP axis is regulated by dysadherin, we investigated its effect on the core Hippo pathway kinases. Strikingly, dysadherin overexpression markedly suppressed the phosphorylation of LATS1/2, the direct upstream kinase that inhibits YAP (Fig. 4d). This suppression was reversed by treatment with a FAK inhibitor, indicating that dysadherin regulates LATS1/2 phosphorylation in a FAK-dependent manner (Fig. 4d). In contrast, the phosphorylation of MST1/2 was not affected.

Among the pluripotency factors, *OCT4* was most strongly regulated by dysadherin (Figs. 1g and 4e). Luciferase reporter assays showed that dysadherin OE increased *OCT4* promoter activity, which was suppressed by verteporfin (Fig. 4f), indicating that YAP activity is required for dysadherin-driven *OCT4* transcription. To further dissect transcriptional mechanism, we used the Eukaryotic Promoter Database (EPD) and the Gene Transcription Regulation Database (GTRD) and identified TEAD2 and TEAD4 as candidate YAP-associated factors at the *OCT4* promoter (Fig. 4g). Knockdown of TEAD2 by selected siRNA (#1), but not TEAD4, significantly reduced *OCT4* expression in YAP5SA-expressing cells, implicating TEAD2 as the primary mediator (Fig. 4h and Supplementary Fig. 4f). Chromatin immunoprecipitation (ChIP) analysis confirmed that TEAD2 binds to the *OCT4* promoter, particularly between −600 and −350 bp (Fig. 4i). Sequential immunoprecipitation using an anti-TEAD2 antibody followed by an anti-YAP antibody revealed specific enrichment at the site corresponding to Primer 5 (Fig. 4i), providing direct evidence that YAP and TEAD2 cooccupy the OCT4 promoter. JASPAR analysis further identified conserved TEAD2 motifs in the promoter regions of *KLF4*, *MYC*, and *SOX2* (Supplementary Fig. 4g). TEAD2 knockdown abrogated the dysadherin-induced upregulation of pluripotency factors at both the transcript and protein levels (Supplementary Fig. 4h).

Collectively, these findings define a dysadherin/FAK/YAP/TEAD2 signaling axis that activates OCT4 and other stemness genes. This transcriptional circuit drives stem-like traits in HCC cells and contributes to their malignant potential.

### The dysadherin/YAP axis modulates drug resistance and PD-L1-mediated immune evasion

**Fig. 5:**
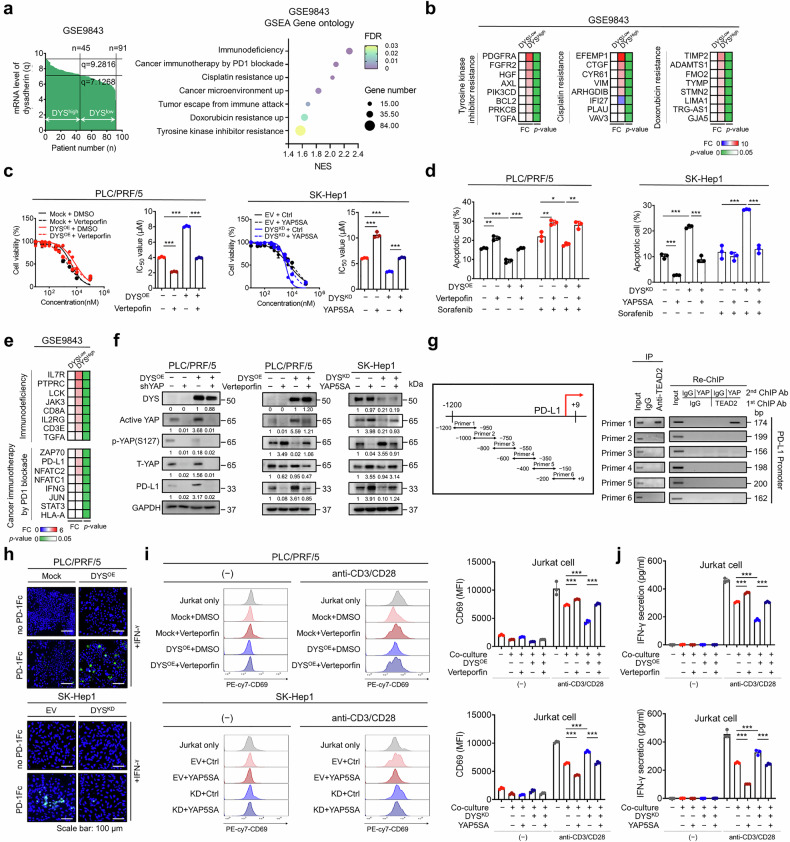
The dysadherin–YAP axis modulates drug resistance and PD-L1–mediated immune evasion in HCC. **a** GSEA of DEGs between dysadherin^high^ and dysadherin^low^ tumors (GSE9843), revealing enrichment of immunotherapy resistance and drug resistance signatures. **b** Heatmaps showing increased expression of gene sets associated with resistance to tyrosine-kinase inhibitors (left), cytostatic drugs (middle), and doxorubicin (right) in dysadherin^high^ HCC tumors. **c** Cell viability and IC_50_ curves of PLC/PRF/5 (dysadherin OE) and SK-Hep1 (dysadherin KD) cells treated with verteporfin or YAP5SA, in the presence of sorafenib. **d** Apoptosis analysis using Annexin V/PI staining in PLC/PRF/5 and SK-Hep1 cells under the same conditions as in (**c**). **e** Heatmap showing elevated expression of immunosuppressive genes and immune checkpoint–related transcripts in dysadherin^high^ tumors from GSE9843. **f** Immunoblot analysis of active YAP, phospho-YAP (S127), total YAP, and PD-L1 in dysadherin-modified cells treated with shYAP, verteporfin, or YAP5SA. **g** ChIP-qPCR showing TEAD2 binding to the PD-L1 promoter region in dysadherin-OE PLC/PRF/5 cells. ChIP-Re-ChIP analysis showing co-occupancy of TEAD2 and YAP on the PD-L1 promoter in SK-Hep1 cells. Sequential immunoprecipitation was performed first with an anti-TEAD2 antibody, followed by an anti-YAP antibody. IgG served as a negative control. **h** Immunofluorescence analysis of PD-L1 and PD-1 binding in dysadherin-OE and KD cells using PD-1-Fc fusion protein staining. Scale bar = 100 μm. Flow cytometry analysis of CD69 expression (**i**) and the measurement of IFN-γ secretion (**j**) in Jurkat T cells co-cultured with dysadherin-modified HCC cells, treated with verteporfin or YAP5SA under CD3/CD28 stimulation. Data are presented as means ± SEM. Statistical significance was determined by unpaired two-tailed Student’s t-tests and one-way ANOVA with Dunnett’s multiple comparison test. *p < 0.05, **p < 0.01, ***p < 0.001

GSEA of the GSE9843 cohort revealed that dysadherin^high^ tumors were enriched for immunosuppression pathways, programmed cell death protein 1 (PD-1) checkpoint signatures, and resistance to tyrosine-kinase inhibitors (TKIs) and chemotherapy (Fig. 5a). These tumors also exhibited the upregulation of multiple drug-resistance genes (Fig. 5b). Functionally, dysadherin OE rendered PLC/PRF/5 cells resistant to sorafenib, and this effect was reversed by verteporfin (Fig. 5c, d and Supplementary Fig. 5a). Conversely, constitutively active YAP (YAP5SA) increased sorafenib resistance in SK-Hep1 and SNU-368 cells, but this effect was blunted by dysadherin KD or an inhibitory peptide (Fig. 5c, d and Supplementary Fig. 5b, c).

Next, we assessed whether dysadherin promotes immune evasion by regulating PD-L1. Dysadherin^high^ tumors exhibited upregulated expression of *PD-L1* and multiple immunotherapy- and immune evasion-related genes in the GSE9843 dataset (Fig. 5e and Supplementary Fig. 5d). Dysadherin OE upregulated *PD-L1* in both adherent and sphere-cultured PLC/PRF/5 cells (Supplementary Fig. 5e), whereas dysadherin KD in SK-Hep1 cells markedly lowered *PD-L1* expression under both culture conditions. This upregulation of PD-L1 expression by dysadherin OE was reversed by YAP silencing and verteporfin treatment (Fig. 5f). In SK-Hep1 cells, dysadherin KD reduced YAP activation and *PD-L1* expression, which was reversed by YAP5SA (Fig. 5f). scRNA-seq analysis further confirmed *PD-L1* enrichment in *FXYD5*^high^ malignant clusters (Supplementary Fig. 5f), which was consistent with a strong positive correlation between *FXYD5* and *PD-L1* expression (Supplementary Fig. 5g). The treatment of inhibitory peptide for dysadherin also suppressed PD-L1 expression in SK-Hep1 spheroids (Supplementary Fig. 5h). Moreover, YAP inhibition by verteporfin in dysadherin-OE PLC/PRF/5 cells markedly reduced the expression of immunosuppressive genes. Conversely, the expression of YAP5SA in dysadherin-KD SK-Hep1 cells restored the expression of these factors, confirming the dependence of dysadherin-PD-L1 regulatory axis on YAP (Supplementary Fig. 5i).

To mechanistically elucidate transcriptional regulation, ChIP-qPCR revealed direct binding of TEAD2 to the *PD-L1* promoter region in dysadherin-OE cells, supporting a YAP–TEAD2-driven transcriptional mechanism (Fig. 5g). Sequential chromatin immunoprecipitation confirmed that YAP and TEAD2 were simultaneously present on the PD-L1 promoter (Fig. 5g). TEAD2 KD resulted in impaired sphere formation and a marked reduction in PD-L1 expression (Supplementary Fig. 5j). Furthermore, functional assays demonstrated that dysadherin OE increased PD-1 binding to PD-L1 over time, whereas dysadherin KD reduced PD-1 engagement (Fig. 5h and Supplementary Fig. 5k). In Jurkat T-cell cocultures, dysadherin OE in PLC/PRF/5 cells suppressed CD69 upregulation and IFN-γ release, and these effects were reversed by verteporfin. Dysadherin KD in SK-Hep1 cells led to reduced CD69 expression and IFN-γ production, which was reversed by YAP5SA expression (Fig. 5i, j). Dysadherin KD in SNU-368 cells similarly relieved T-cell suppression, an effect that was reversed by the expression of YAP5SA (Supplementary Fig. 5l).

Collectively, these results show that dysadherin drives sorafenib resistance and PD-L1–mediated immune escape via YAP activation. Targeting the dysadherin–YAP–PD-L1 axis may overcome chemoresistance and restore antitumor immunity in dysadherin^high^ liver cancers.

### The dysadherin/YAP axis drives HCC progression and facilitates immune evasion in vivo

**Fig. 6:**
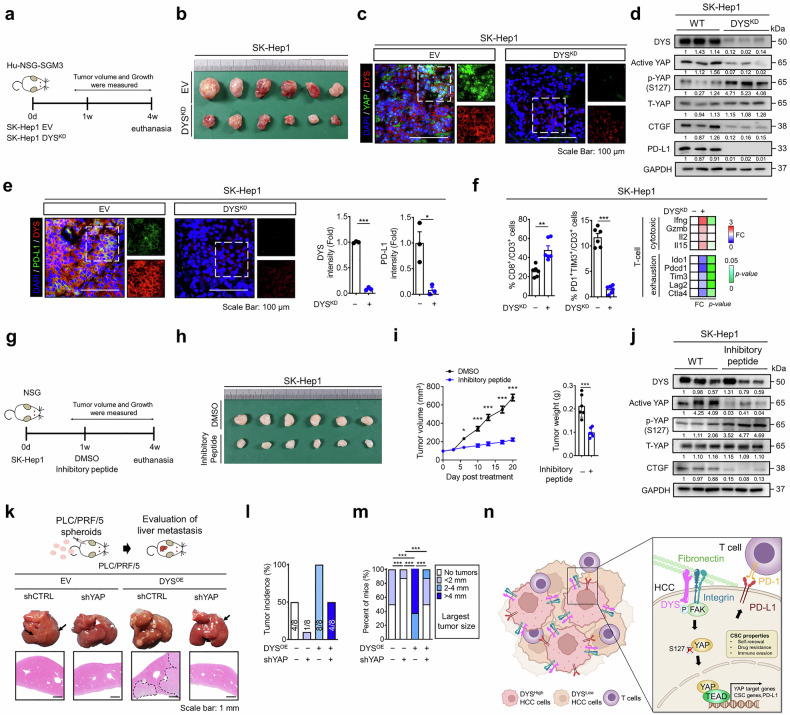
The dysadherin–YAP axis drives HCC progression and facilitates immune evasion in vivo. **a** Schematic overview of the humanized mouse model (Hu-NSG-SGM3) used for in vivo tumor studies. **b** Tumor growth of SK-Hep1 cells with or without dysadherin knockdown (DYS^KD^) in the humanized mouse model. Immunofluorescence (**c**, **e**) and immunoblot (**d**) analysis of dysadherin, YAP, CTGF, and PD-L1 expression in SK-Hep1 tumors. Scale bar = 100 μm. **f** Left: Flow cytometric quantification of tumor-infiltrating CD8⁺ T cells and PD1⁺TIM3⁺ exhausted T cells. Right: Heatmap of cytotoxic and exhaustion marker gene expression profiles of T cells in DYS^KD^ versus control tumors. **g** Schematic overview of the in vivo peptide treatment protocol in NSG mice. Representative tumor images (**h**) and quantification of tumor growth and weight (**i**) in SK-Hep1 xenografts treated with the dysadherin-inhibitory peptide versus vehicle (DMSO) for 28 days (n = 6 mice/group). **j** Immunoblotting of dysadherin, YAP activation markers, and CTGF expression in tumor tissues after peptide treatment. **k** Experimental design for splenic injection model of liver metastasis using PLC/PRF/5 spheroids with or without dysadherin OE and shYAP. Scale bar = 1 mm. Quantification of tumor incidence (**l**), tumor size (**m**) in the liver metastasis model. **n** Schematic diagram of integrin-FAK-YAP-TEAD axis-mediated stemness acquisition and immune escape in liver cancer. Created with BioRender.com. Data are presented as means ± SEM. Statistical significance was determined by unpaired two-tailed Student’s t-tests and one-way ANOVA with Dunnett’s multiple comparison test. *p < 0.05, **p < 0.01, ***p < 0.001

To assess the in vivo significance of dysadherin in HCC, we employed a humanized mouse model (Hu-NSG-SGM3) reconstituted with human CD34⁺ hematopoietic stem cells (Fig. 6a). Flow cytometry of peripheral blood confirmed successful engraftment, with more human CD45⁺ cells in the reconstituted mice than in the control mice (Supplementary Fig. 6a). In this model, dysadherin KD in SK-Hep1 cells significantly suppressed tumor growth and reduced tumor weight (Fig. 6b and Supplementary Fig. 6b). These effects were accompanied by decreased dysadherin expression, reduced nuclear YAP intensity, and attenuated the expression of PD-L1 (Fig. 6c, d and Supplementary Fig. 6c, d). IF imaging revealed strong dysadherin and PD-L1 expression in SK-Hep1 tumors, whereas dysadherin-deficient tumors exhibited reduced signals (Fig. 6e). Immunofluorescence analysis on HCC patient tissues validated a strong positive correlation, where tumors with high dysadherin expression showed significantly higher levels of PD-L1 expression (Supplementary Fig. 6e). Flow cytometry and transcriptomic profiling revealed enhanced infiltration of CD8⁺ cytotoxic T cells, along with a pronounced decrease in PD1⁺TIM3⁺ exhausted T cells, in tumors derived from dysadherin-KD cells (Fig. 6f).

To further characterize the immune microenvironment, we analyzed scRNA-seq data from HCC tumors, comparing *FXYD5*^high^ and *FXYD5*^low^ regions. High *FXYD5* expression was associated with an increased proportion of regulatory T cells (Tregs) and exhausted CD8 T cells (CD8 Tex), along with a reduced proportion of cytotoxic CD8^+^ T cells (CTLs) (Supplementary Fig. 6f), indicating a shift toward an immunosuppressive T-cell landscape. Spatial transcriptomic analysis further showed that *FXYD5*^High^ regions were infiltrated by immunosuppressive SPP1^+^ TAMs and protumorigenic CAFs (Supplementary Fig. 6g–i). These regions also displayed a pronounced immune exhaustion signature, with elevated expression of multiple T-cell-associated checkpoint molecules and exhaustion markers, including CTLA4, LAG3, TIGIT, PDCD1, and TOX (Supplementary Fig. 6j, k). Together, these findings indicate that dysadherin shapes a spatially organized, functionally exhausted, and immunosuppressive TME.

We next tested the therapeutic potential of a dysadherin-inhibitory peptide in mice bearing subcutaneous SK-Hep1 tumors. Compared to vehicle control treatment, peptide treatment substantially reduced tumor growth (Fig. 6g–i), accompanied by decreased YAP activation and decreased expression of its downstream targets (Fig. 6j and Supplementary Fig. 7a). Pharmacokinetic analysis revealed that the peptide remained stable in vitro and in vivo, with a short plasma half-life and no systemic accumulation (Supplementary Fig. 7b–d). No systemic toxicity was observed, as confirmed by serum biochemistry and organ function panels (Supplementary Fig. 7e).

To assess metastatic potential, we employed a splenic injection liver metastasis model using PLC/PRF/5 spheroids. Dysadherin OE markedly increased the incidence and burden of hepatic metastasis, whereas YAP KD abolished these effects (Fig. 6k–m and Supplementary Fig. 7f, g). Histological analysis confirmed fewer and smaller metastatic nodules in YAP-silenced tumors, and immunoblotting revealed decreased CTGF and OCT4 expression (Fig. 6k and Supplementary Fig. 7h).

Collectively, these findings establish that dysadherin orchestrates integrin/FAK/YAP signaling to drive cancer stemness and immune evasion through PD-L1 induction (Fig. 6n). The dysadherin/YAP axis plays a pivotal cell-intrinsic role in HCC progression and shapes a cold tumor microenvironment. Importantly, genetic ablation or pharmacologic inhibition of dysadherin effectively curbs tumor growth, relieves immune suppression, and blocks metastasis. Targeting this axis may provide a new avenue for therapeutic approaches in advanced, treatment-resistant HCC.

## Discussion

We identified dysadherin as a key driver of cancer stemness, tumor progression, and immune evasion in HCC. Integrated single-cell and bulk transcriptomic analyses demonstrated its enrichment in malignant epithelial cells and strong association with poor prognosis. Mechanistically, dysadherin activates YAP signaling via the integrin–FAK axis, leading to YAP–TEAD2-dependent expression of pluripotency genes such as *OCT4*, *KLF4*, *MYC*, and *SOX2*, enhancing stem-like traits and resistance to sorafenib. Dysadherin also upregulates PD-L1 through YAP–TEAD2, promoting immune escape. Genetic or pharmacological inhibition of dysadherin suppresses tumor growth and metastasis and restores cytotoxic T-cell function. These results imply that the dysadherin/YAP axis represents a promising therapeutic target in HCC.

Dysadherin has been implicated in promoting tumor aggressiveness by modulating the tumor microenvironment (TME) and epithelial plasticity in various solid tumors.^14,32^ In this study, we demonstrated that dysadherin also maintains CSC properties in HCC. While previous studies suggested a link between dysadherin and CSC traits, showing that dysadherin overexpression enhances self-renewal and tumor-initiating potential, the underlying mechanisms remain unclear.^14^ Our results revealed that dysadherin activates the FAK/YAP/TEAD2 pathway to induce key pluripotency genes, thereby promoting CSC maintenance and therapy resistance. These findings are consistent with earlier reports that YAP/TAZ signaling supports CSC populations and drives drug resistance in breast, bladder, and glioblastoma cancers,^33–35^ and that YAP regulates SOX2 and Nanog, reinforcing its central role in tumor plasticity.^35^ Our work presents initial evidence that TEAD2 binds to the OCT4 promoter, a result that remains to be validated with additional experimental approaches despite its potential significance.

YAP activation is a key mediator of therapy resistance in multiple cancers, including HCC.^23,24^ We identified a dysadherin/YAP axis that drives sorafenib resistance by promoting YAP nuclear localization and transcriptional activity, thereby enhancing stemness and survival under treatment stress. Dysadherin inhibition, either genetically or with a peptide, restores sorafenib sensitivity and reduces CSC traits, indicating that YAP-dependent stem plasticity underlies chemoresistance. YAP has also been reported to reprogram metabolism and DNA repair,^36,37^ drive lipid metabolic adaptation,^38^ and stabilize NRF2 signaling to promote redox balance and survival in HCC.^39^ These findings position dysadherin as a druggable upstream target to overcome YAP-mediated resistance. Interestingly, partial attenuation of sorafenib resistance by dysadherin KD in YAP5SA cells suggests that dysadherin may also promote resistance through YAP-independent pathways, potentially involving FAK-driven PI3K/AKT and MAPK/ERK signaling.

Tumor cells evade immune surveillance by upregulating immune checkpoints, contributing to progression and immunotherapy resistance.^40,41^ YAP has been implicated in PD-L1 regulation across multiple cancers, where its activation enhances PD-L1 expression and suppresses T-cell function.^25,42^ In this study, we identified dysadherin as an upstream activator of the YAP–TEAD2 complex in HCC that directly drives PD-L1 transcription and impairs cytotoxic T-cell activity in a humanized HCC model. Dysadherin inhibition reduces both cancer stemness and immune evasion, highlighting its potential as a therapeutic target and supporting combination strategies with PD-1/PD-L1 inhibitors in immune-refractory HCC. Our single-cell and spatial transcriptomics analyses further revealed how dysadherin contributes to a “cold” tumor phenotype. In particular, the spatial colocalization of *FXYD5*^High^ malignant cells with SPP1^+^ TAMs and CAFs, together with the marked upregulation of T-cell-associated exhaustion markers in these regions, suggests that dysadherin not only sustains CSC traits but also remodels the TME into a protumorigenic niche that shields tumors from immune attack.

In a prior study, we identified fibronectin-binding sequences within the extracellular domain of dysadherin and developed a synthetic peptide that blocks this interaction, thereby disrupting integrin/FAK/YAP signaling and suppressing tumor growth and metastasis in colorectal cancer.^29^ The peptide selectively reduced the viability of dysadherin-expressing cancer cells. In the present study, we applied this peptide to HCC models and reported that it significantly inhibited YAP activation, PD-L1 expression, and sphere-forming ability, ultimately restoring antitumor immunity and enhancing drug responsiveness. These findings suggest that the dysadherin inhibitory peptide effectively disrupts the dysadherin/YAP/PD-L1 axis and represents a promising strategy to target both cancer stemness and immune evasion in dysadherin^high^ tumors.

A critical question is what drives dysadherin expression in HCC. Our IPA results predict that *FXYD5* expression may be upregulated through the activation of major oncogenes (e.g., *CTNNB1*, *MET*, and *TERT*) and the loss of tumor suppressors (e.g., *TP53* and *AXIN1*), suggesting that dysadherin upregulation could represent a convergent outcome of primary driver mutations in HCC (Supplementary Fig. 7i). However, these predicted upstream regulators remain to be experimentally validated. Moreover, although our findings indicate that dysadherin contributes to immune evasion and therapy resistance, in vivo confirmation is still needed. Evaluating the efficacy of dysadherin inhibition in combination with TKIs and immune checkpoint inhibitors in preclinical HCC models is critical to clarify its therapeutic potential and clinical relevance.

In conclusion, this study highlights dysadherin as a key upstream regulator of YAP signaling that promotes cancer stemness, immune evasion, and therapeutic resistance in HCC. Through characterization of the dysadherin/FAK/YAP/TEAD2 axis, we uncovered a mechanistic link between cell adhesion and the transcriptional activation of genes associated with pluripotency, chemoresistance, and immune checkpoints. Targeting dysadherin, either genetically or with an inhibitory peptide, suppresses tumor growth and restores antitumor immune responses. Our results suggest that dysadherin might be a promising therapeutic target with important translational relevance for patient stratification and therapeutic strategies. Patients with high dysadherin expression and YAP activation may benefit the most from dysadherin-targeted therapy, and combining dysadherin inhibition with TKIs or immune checkpoint inhibitors could resensitize refractory tumors and address a major clinical challenge in advanced HCC.

## Materials and methods

### Cell culture

The human HCC cell lines, HepG2, SNU368, and SK-Hep1 were obtained from the Korean Cell Line Bank (KCLB, Seoul, Republic of Korea). The Jurkat T-lymphocyte cell line, used in T-cell co-culture experiments, was also obtained from the KCLB. The human normal hepatocyte cell line THLE3 was purchased from American Type Culture Collection (ATCC, Rockville, MD, USA). The PLC/PRF/5, AV1 (mock-transfected (Mock) PLC/PRF/5), and AL3-1 (dysadherin PLC/PRF/5 OE) cell lines were generously provided by Dr. Setsuo Hirohashi, as previously reported.^6^ Cell lines were handled strictly according to supplier’s guidelines. To ensure culture integrity, Mycoplasma was tested every 6 months using the e-Myco™ Mycoplasma Detection Kit (iNtRON Biotechnology, Seongnam, Korea). All analyses were performed only on cultures that did not exceed 20 passages after initial thaw.

### Small interfering RNA-based silencing and generation of dysadherin-deficient cells

For gene silencing, siRNAs targeting *FXYD5*, *KLF4*, *OCT4*, and *MYC*, together with a nontargeting control siRNA (Bioneer, Daejeon, South Korea), were employed. siRNA transfection was conducted as previously described.^43^ The siRNA sequences are provided in Supplementary Table 1. Stable dysadherin-deficient cell lines were generated via short hairpin RNA (shRNA) as previously described.^43^

### Mice

Mice were maintained in specific pathogen–free facilities at controlled temperature (~24 °C) and humidity (~50%), with a 12 h light–dark cycle. Animal welfare was assessed routinely according to the IACUC guidelines of GIST, and humane endpoints included >20% loss of body weight, lethargy, reduced mobility, hunched posture, or rapid tumor expansion. Animals that reached any of these predetermined endpoints were euthanized. To induce HCC, male *Fxyd5*^+/+^ and *Fxyd5*^−/−^ mice received intraperitoneal (i.p.) injections of DEN (25 mg/kg) prepared in sterile 0.9 saline, on postnatal day 14. Starting at week 8, mice were administered CCl_4_ (0.5 mL/kg, diluted in corn oil) via i.p. injection twice weekly for 16 weeks. Tumor burden was evaluated at 19 and 24 weeks. Tumors were classified as small (<2 mm), medium (≥2 mm and <4 mm), or large (≥4 mm).

For in vivo limiting dilution assay (LDA), NSG mice (NOD.Cg-Prkdc^scid^ Il2rg^tm1Wjl/SzJ^; Jackson Laboratory, Bar Harbor, ME, USA) were subcutaneously injected with empty vector (EV) or dysadherin KD SK-Hep1 cells at serial dilutions (50000, 10000, 5000, 1000 cells/mouse, n = 6/group). The tumor incidence was determined by definitive necropsy after 42 days. Graphs for LDA were acquired, and statistical analysis was performed via software provided by Walter+Eliza Hall Bioinformatics (http://bioinf.wehi.edu.au/software/elda/).

A splenic injection experiment was conducted to evaluate metastasis. In this model, EV-transfected control cells or dysadherin-KD sphere cells were administered to the spleens of male NSG mice at 8 weeks of age followed by splenectomy (1 × 10^6^ cells/mouse); surviving cells that migrated out of the spleen and grew in distant organs contributed to the formation of liver metastases. After sacrifice, the livers were harvested to quantify metastasis.

For subcutaneous xenografts, SK-Hep1 cells were subcutaneously infused into 5-week-old male NSG mice (5 × 10^6^ cells/mouse). Tumor growth was measured based on tumor volume by calculating (length × width^2^)/2 for 28 days after cell inoculation (n = 6 for each group). Upon reaching 100 mm^3^, mice were treated with inhibitory peptide three times per week for the experimental period.

For the generation of humanized mice, female NSG-SGM3 mice (Jackson Laboratory) were selected, as female hosts show higher efficiency for CD34⁺ cell engraftment. Transplantation was carried out in 4-week-old mice. NSG-SGM3 mice received 1 × 10^5^ human CD34^+^ cells via intravenous route. Humanization was confirmed by flow cytometry of CD45^+^ cells in peripheral blood after 8 weeks. Blood was obtained from the retro-orbital plexus. For immunostaining, PE anti-mouse CD45 and APC anti-human CD45 antibodies were added, and samples were incubated for 20 min at room temperature in the dark. Following RBC lysis with commercial lysis buffer (Sigma-Aldrich), samples were subjected to flow cytometric analysis using a BD Accuri™ C6 instrument (Becton Dickinson, Sunnyvale, CA, USA). Flow cytometry data were processed using FlowJo software (Tree Star Inc., Ashland, OR, USA). Following confirmation of human cell reconstitution, SK-Hep1 cells (1 × 10^7^ cells/mouse) were inoculated subcutaneously in each mice. Tumor growth was monitored for 21 days post-inoculation by measuring tumor volume until necropsy (n = 6 for each group).

### Aldefluor assay

The ALDH enzymatic activity of the cells was determined by ALDEFLUOR kit (Stem Cell Technologies, Vancouver, BC, Canada). Flow cytometric detection of cells expressing ALDH was performed. Diethylaminobenzaldehyde (DEAB), a specific ALDH inhibitor, was utilized to stain negative control cells.

### Clonogenic survival assay

A clonogenic assay was performed as previously described.^43,44^ Briefly, cells were seeded in 12-well plates (200 cells/well) and cultured for 14 days. Colonies >50 μm in diameter were counted after crystal violet staining (n = 3/group).

### Luciferase reporter assay

Luciferase activity driven by the OCT4 promoter was measured using a reporter vector (GeneCopoeia, Rockville, MD, USA). For transfection, plasmid DNA, β-galactosidase control, and Lipofectamine (Sigma-Aldrich) were mixed at 1 μg:1 μg:2 μL in 300 μL of OptiMEM (Thermo Fisher Scientific, Waltham, MA, USA) medium per well. Following transfection, cells were treated with verteporfin (1.5 μmol/L) and incubated for 24 h, after which cell lysates were prepared. Luciferase readout was performed using Renilla luciferase assay system (Promega, Madison, WI, USA) and quantified with a SpectraMax L luminometer (Molecular Devices, San Jose, CA, USA). Relative luminescence values were normalized to β-galactosidase activity.

### ChIP assay

ChIP assay was conducted utilizing the EZ-Magna ChIP™ A/G Kit (MilliporeSigma, Burlington, MA, USA). Briefly, 6 × 10^6^ cells were fixed with formaldehyde (1%), quenched with glycine, and lysed. Chromatin was sheared by sonication (200–700 bp) and immunoprecipitated overnight with an anti-TEAD2 antibody. Protein A agarose beads were used for capture, and DNA was purified and subjected to PCR using primers specific to TEAD2-binding sites in the OCT4 and PD-L1 promoters.

### PD-L1 and PD1 interaction assay

The interaction between HCC cell-derived PD-L1 and PD-1 was evaluated. 1 × 10^5^ HCC sphere cells were seeded in 12-well plates and treated with 500 IU/ml IFN-γ for 24 h to induce PD-L1 expression. After incubation, the cells were stained with PE-labeled PD-1 (3 μg/mL, Sigma-Aldrich). The binding of PD-1 to PD-L1 was measured and quantified every 8 h.

### CD3^+^ T-cell isolation and flow cytometry analysis

Freshly resected mouse tumor samples were dissociated using enzymatic digestion and mechanical dissociation. CD3^+^ T-cells were isolated from the tumor microenvironment using CD3 MicroBeads (Miltenyi Biotec GmbH, Bergisch Gladbach, Germany). Following isolation, CD3^+^ T-cells were stained with PE anti-human CD8 (BioLegend, Inc., San Diego, CA, USA), FITC anti-human PD-1 (CD279) (BioLegend), and APC anti-human TIM-3 (CD366) (BioLegend). Antibody-conjugated cells were analyzed using BD AccuriTM flow cytometer.

### Statistical analyses

Data are presented as means ± standard errors of the means (SEMs). For comparisons between two groups, statistical significance was assessed using Student’s t-test or two-way ANOVA with Bonferroni multiple comparison test. For analyses involving three or more groups, one-way ANOVA with Dunnett’s multiple comparison test was applied. Statistical analyses were performed using GraphPad Prism (GraphPad Software, San Diego, CA, USA). The number of biological replicates for each experiment is indicated in the figure legends. Statistical significance is denoted as follows: * p < 0.05; ** p < 0.01; *** p < 0.001.

## Supplementary information

Supplementary Information

Supplementary Information

Supplementary Information

## Data Availability

The authors state that all of the data generated in the present study are included in the manuscript and Supplementary Materials. Other information regarding this study is available upon request.
