# Supplementary material for: Dysadherin/YAP axis fuels stem plasticity and immune escape in liver cancer
Source: Signal Transduct Target Ther. 2025 Dec 29;10:421. doi: 10.1038/s41392-025-02520-4 (PMC12745361; doi:10.1038/s41392-025-02520-4)
Supplement: Supplementary file 1 — Supplementary Information [file 41392_2025_2520_MOESM1_ESM.docx]

Supplementary Materials for

**Dysadherin/YAP axis fuels stem plasticity and immune escape in liver cancer**

Tae-Young Jang^1#^, So-El Jeon^1#^, Hyeon-Ji Yun^1^, Choong-Jae Lee^1^, Da-Ye Lim^1^, Sang Hoon Lee^2^, Dajun Lee^3^, Seungwon Lee^3^, Jungmin Choi^3^, Hyung-Sik Kim^4*^, Jeong-Seok Nam^1*^

^1^School of Life Sciences, Gwangju Institute of Science and Technology, Gwangju 61005, Republic of Korea

^2^Geninus Inc, Seoul 05836, Republic of Korea

^3^ Department of Biomedical Sciences, Korea University College of Medicine, Seoul 02841, Republic of Korea

^4^Department of Oral Biochemistry, Dental and Life Science Institute, Pusan National University, Yangsan, 50612, Republic of Korea

Correspondence to: Hyung-Sik Kim (hskimcell@pusan.ac.kr) or Jeong-Seok Nam (namje@gist.ac.kr)

These authors contributed equally: Tae-Young Jang, So-El Jeon

**This PDF file includes:**

Materials and Methods

Figures. S1 to S7

Tables S1 to S4

**Materials and Methods**

Bioinformatics assessment

Single-cell RNA-seq data from the GSE166635 dataset were reanalyzed using Seurat for normalization, identification of variable features, data scaling, clustering, and generation of uniform manifold approximation and projection (UMAP) plots using default parameters. Cell type annotations were imported from the original study, and epithelial cells were categorized as normal or malignant. Malignant cells were further classified as dysadherin-expressing or non-expressing using CopyKAT. For trajectory analysis, Monocle 2 was used to infer cell differentiation trajectories based on unsupervised pseudotime ordering. To evaluate the transcriptional activity of the YAP pathway, a YAP signature score was calculated for each cell using the AddModuleScore function in Seurat (v4.0). The YAP signature gene set included the following genes: TNS2, SERPINE1, CTGF, AXL, TNS1, BIRC5, SGK1, TGM2, CYR61, AMOTL2, ETV5, and FLNA. The module score was computed based on the average expression of these genes, subtracted by the aggregated expression of a set of control feature genes.

For Gene set enrichment analysis (GSEA), briefly, differentially expressed genes (DEGs) were obtained from bulk RNA-seq datasets GSE9843 and GSE54236 by comparing HCC patients with high versus low dysadherin expression (divided by the median). The total DEG lists (p < 0.05) were ranked and subjected to GSEA using the Java-based GSEA tool (<http://www.broadinstitute.org/gsea/>) with 1,000 permutations, minimum gene set size of 15, and maximum of 500, against C2 (curated) and C6 (oncogenic) gene sets. Ingenuity Pathway Analysis (IPA) was also performed to identify significantly enriched diseases, biological functions, and upstream regulators associated with dysadherin expression.

Sphere formation assay

To examine their self-renewal ability, cells were seeded at various densities in poly-HEMA-coated plates (Sigma‒Aldrich, St. Louis, MO, USA) under non-adherent, sphere–forming condition. After incubation, spheres were counted and measured (n = 3/group). Sphere viability was assessed using the CellTiter-Blue Cell Viability Assay (Sigma-Aldrich) according to the manufacturer’s instructions. Fluorescence was measured at 560 nm excitation and 590 nm emission using the Varioskan LUX multimode reader (Thermo Fisher Scientific, Waltham, MA, USA).

RNA isolation and real-time quantitative polymerase chain reaction (RT-qPCR)

Total RNA was isolated using RNAiso reagent (Takara, Shiga, Japan), and purity was confirmed by 260/280 and 260/230 ratios. cDNA templates were synthesized from 0.5 μg of total RNA using the PrimeScriptTM 1st strand cDNA Synthesis Kit (Takara) with random primers. qPCR was performed using Power SYBR Green PCR Master Mix and Step-One Real-time PCR systems (Applied Biosystems, Foster City, CA, USA). The primers used are listed in Supplementary Table S2.

Protein isolation and immunoblot analysis

Cells were lysed in RIPA buffer for 20 minutes on ice, and protein concentrations were measured using a bicinchoninic acid (BCA) assay kit (Thermo Fisher Scientific). The proteins were denatured with SDS (Sigma‒Aldrich) and by boiling at 95 °C for 5 minutes. Equal amounts (10 µg) were resolved by 10% SDS-PAGE and transferred to PVDF membranes (Millipore, Burlington, MA, USA). The membranes were blocked with 10% bovine serum albumin (Sigma‒Aldrich) and incubated overnight at 4 °C with the indicated primary antibodies. The membranes were then incubated with secondary antibodies conjugated to horseradish peroxidase (HRP). After washing, membranes were incubated with HRP-conjugated secondary antibodies, and signals were developed using SuperSignal™ West Dura Substrate (Thermo Fisher Scientific) and visualized using a Digital 8 Imaging System (ProteinSimple, San Jose, CA, USA). The antibodies used for immunoblot analyses are listed in Supplementary Table S3.

Immunofluorescence

Cells and tissues were fixed and incubated with primary antibodies, followed by fluorophore-conjugated secondary antibodies. Nuclei were counterstained with 4′,6-diamidino-2-phenylindole (DAPI; Sigma‒Aldrich). Fluorescence signals were visualized using an Axio Imager 2 (Carl Zeiss, Oberkochen, Germany) at a total magnification of 200x or 1000x. The antibodies used are listed in Table S3.

Apoptosis assay

The rate of cell apoptosis was quantified using apoptosis assays performed with an Annexin V-Fluorescein isothiocyanate (FITC) Apoptosis Detection Kit I. (BD Biosciences, San Jose, CA, USA). Cells were washed twice with cold PBS to yield single-cell suspensions at a concentration of 1×10⁶ cells/mL. The suspension was transferred to a tube containing 5 µL of FITC, annexin V, and propidium iodide (PI). After gentle vortexing, the mixture was incubated at room temperature for 15 min in the dark. After incubation, 400 µL of 1 x binding buffer was added to each sample prior to flow cytometry analysis. FACS analysis was performed using a BD AccuriTM flow cytometer (BD Biosciences). FACS data were analyzed using FlowJo software (TreeStar, San Carlos, CA, USA) as described in our previous report.

Co-culture assay

The rate of T-cell activation after co-culture with the HCC cells were evaluated by direct co-culture system. The assay was performed in 24-well plates. HCC cells (1x10^4^) were seeded in the plates and cultured for 24 hours to allow attachment. The cells were transfected with or without YAP5SA and treated with or without verteporfin. Following 24 hours of treatment, the tumor cells were incubated for an additional 24 hours before introducing Jurkat cells (4x10^5^). After 1hour of stabilization, T-cell activation was induced by adding soluble anti-CD3 (2 μg/mL, eBioscience, San Diego, CA, USA), soluble anti-CD28 (1 μg/mL, eBioscience), and anti-mouse Ig (5 μg/mL, Southern Biotech, Birmingham, AL, USA). After 24 hours of co-culture, the supernatant was collected for an IFN-γ ELISA assay, and Jurkat cells were harvested for FACS analysis to assess T-cell activation. The cells were stained with the PE-cy7 conjugated anti-CD69 antibody in FACS buffer for at least 1hour at 4 °C in the dark and analyzed on BD AccuriTM flow cytometer and data were processed using FlowJo software.

Enzyme-linked immunosorbent assay (ELISA) for IFN-γ

The concentration of IFN-γ in cell-free supernatants was measured using a Human IFN-γ ELISA Kit (R&D Systems, Minneapolis, MN, USA), following the manufacturer’s instructions.

In vitro stability test

To evaluate plasma stability, inhibitory peptide was diluted to a final concentration of 10 μM in human plasma (Biochemed, Seoul, Republic of Korea) and incubated at 37 °C for 0, 30, and 120 minutes. At each time point, samples were removed and mixed with an internal standard solution containing chlorpropamide in methanol (0.1% formic acid). The mixtures were vortexed for 5 minutes, followed by centrifugation at 15,000 rpm for 5 minutes at 4 °C. The resulting supernatants were analyzed using LC-MS/MS to determine the remaining concentration of the peptides over time and assess their plasma stability.

LC-MS/MS Analysis

LC-MS/MS analysis was conducted using a Shimadzu Exion LC system coupled with a QTRAP 6500 plus LC-MS/MS system (Applied Biosystems, USA). Chromatographic separation was performed on an ACQUITY Premier Peptide BEH C18 column (2.1 × 100 mm, 1.7 μm particle size; Waters, Ireland) using a gradient elution method with 0.1% formic acid in water (solvent A) and 0.1% formic acid in acetonitrile (solvent B). Data acquisition was carried out in MRM (Multiple Reaction Monitoring) mode using Analyst software (version 1.7.3), and the concentrations of analytes were quantified based on internal standard normalization.

In vivo stability test

Male BALB/c mice were administered a single intravenous dose of Inhibitory peptide (10 mg/kg). Blood samples were collected at predetermined time points (0, 0.08, 0.25, 0.5, 1, 2, 4, 8, 10, 12, 24, and 48 hours) via the retro-orbital plexus using heparinized capillary tubes, which were immediately placed on ice. Plasma was separated by centrifugation at 5,000 rpm for 15 minutes at 4 °C, and the supernatants were stored at –80 °C until analysis. For sample preparation, 20 μL of plasma was mixed with 180 μL of methanol containing chlorpropamide (internal standard) and 0.1% formic acid. After vortexing for 5 minutes and centrifugation at 15,000 rpm for 5 minutes at 4 °C, the supernatant was collected for analysis.

Quantification of plasma peptide concentrations was performed using LC-MS/MS. Pharmacokinetic parameters, including AUC, T₁/₂, Cₘₐₓ, and Tₘₐₓ, were calculated using Phoenix WinNonlin software (version 6.4, Pharsight, USA) via non-compartmental analysis.


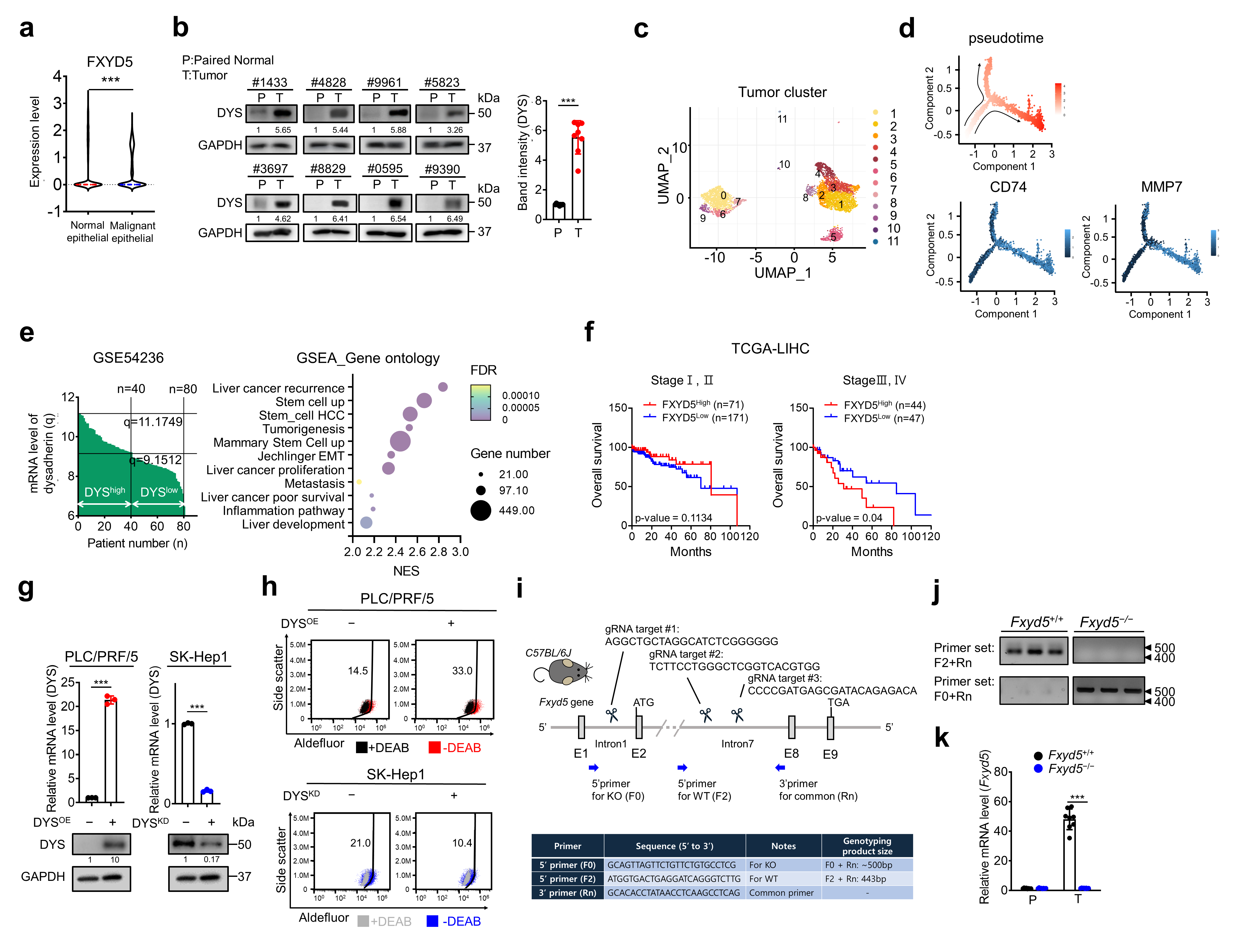


Figure. S1. Dysadherin expression defines CSC populations and promotes tumor initiation in HCC

**a** Violin plots showing *FXYD5* expression in normal versus malignant epithelial cell populations from HCC single-cell RNA-seq data (GSE166635). **b** Immunoblot analysis of dysadherin protein expression in tumor (T) and paired adjacent normal tissues (P) from eight HCC patients. **c** UMAP visualization of tumor epithelial clusters from GSE166635, color-coded by cluster identity. **d** Pseudotime trajectory analysis using Monocle2 depicting the inferred differentiation path from normal-like to malignant epithelial states. **e** Gene set enrichment analysis (GSEA) of differentially expressed genes between dysadherin^high^ and dysadherin^low^ tumors in the GSE54236 cohort (false discovery rate [FDR] < 0.05). **f** Kaplan-Meier survival analysis for overall survival based on FXYD5 expression in the TCGA-LIHC cohort, stratified by tumor stage (Stage I/II, n=242; Stage III/IV, n=91). Statistical significance was assessed by the log-rank test. **g** RT-qPCR and immunoblot validation of dysadherin overexpression (OE) and knockdown (KD) in PLC/PRF/5 and SK-Hep1 cells. **h** Aldefluor assay assessing CSC populations in dysadherin-OE and -KD cells. **i** Schematic of CRISPR-Cas9–mediated deletion of exons 2–7 of *Fxyd5*, including positions of genotyping primers. **j** PCR-based validation of dysadherin knockout (*Fxyd5*^-/-^) in mouse genomic DNA. **k** qRT-PCR analysis of *Fxyd5* mRNA expression in tumor (T) and paired adjacent normal tissues (P) from wild-type (*Fxyd5*^+/+^) and knockout (*Fxyd5*^-/-^) mice in the DEN/CCl_4_-induced HCC model. Data are presented as means ± SEM. Statistical significance was determined by unpaired two-tailed Student’s t-tests for comparisons between two groups, and one-way ANOVA with Dunnett’s multiple comparison test for comparisons among three or more groups. *p < 0.05, **p < 0.01, ***p < 0.001.


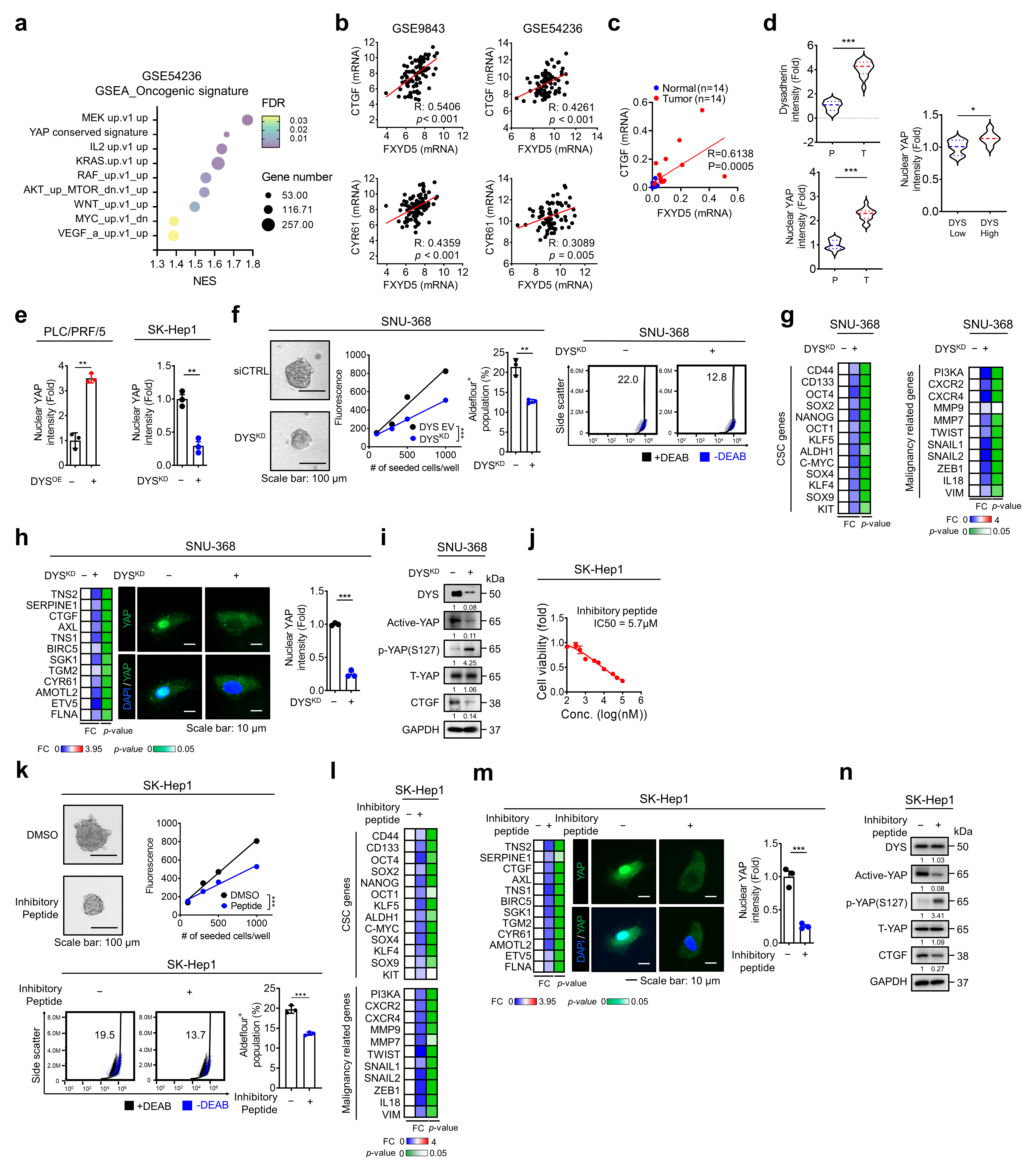


Figure. S2. Pharmacological inhibition of dysadherin suppresses YAP activation and cancer stem-like features in HCC

**a** GSEA of DEGs between dysadherin^high^ and dysadherin^low^ tumors in the GSE54236 cohort. **b** Correlation analysis of *FXYD5* mRNA levels and YAP target gene expression (*CTGF* and *CYR61*) in two HCC patient cohorts (GSE9843 and GSE54236). **c** Correlation between *FXYD5* and *CTGF* mRNA expression in paired normal and tumor liver tissues from 14 HCC patients. **d** Quantification of IF signal intensity for dysadherin and nuclear YAP in tumor (T) and paired adjacent normal tissues (P) from HCC patients (*n*=14). **e** Quantitative analysis of dysadherin expression and nuclear YAP intensity in PLC/PRF/5 and SK-Hep1 cells with or without dysadherin OE or KD. **f** Sphere formation assay (left) and Aldefluor assay (right) showing impaired CSC properties following dysadherin KD in SNU-368 cells. Scale bar = 100 µm. **g** Heatmap showing downregulation of dysadherin- and YAP-regulated gene sets after dysadherin KD (siDYS) in SNU-368 cells. **h** Immunofluorescence staining and quantification showing nuclear YAP localization in SNU-368 cells. Scale bar = 10 µm. **i** Immunoblot analysis showing active YAP and increased inhibitory p-YAP (S127) following dysadherin KD in SNU-368 cells. **j** IC_50_ determination of the dysadherin-inhibitory peptide in SK-Hep1 cells using MTT viability assay. **k** Sphere formation (left) and Aldefluor assay (right) showing reduced CSC-like properties in SK-Hep1 cells treated with the inhibitory peptide. Scale bar = 100 μm. **l** Heatmaps showing downregulation of CSC-associated and malignancy-related genes following peptide treatment in SK-Hep1 cells. **m** Heatmap of YAP transcriptional target genes and IF images showing reduced active YAP nuclear localization after inhibitory peptide treatment in SK-Hep1 cells. Scale bar = 100 μm. **n** Immunoblot analysis confirming decreased active YAP, increased phospho-YAP (S127), and reduced CTGF expression in peptide-treated SK-Hep1 cells. Data are presented as means ± SEM. Statistical significance was determined by unpaired two-tailed Student’s t-tests for comparisons between two groups, and one-way ANOVA with Dunnett’s multiple comparison test for comparisons among three or more groups. *p < 0.05, **p < 0.01, ***p < 0.001.


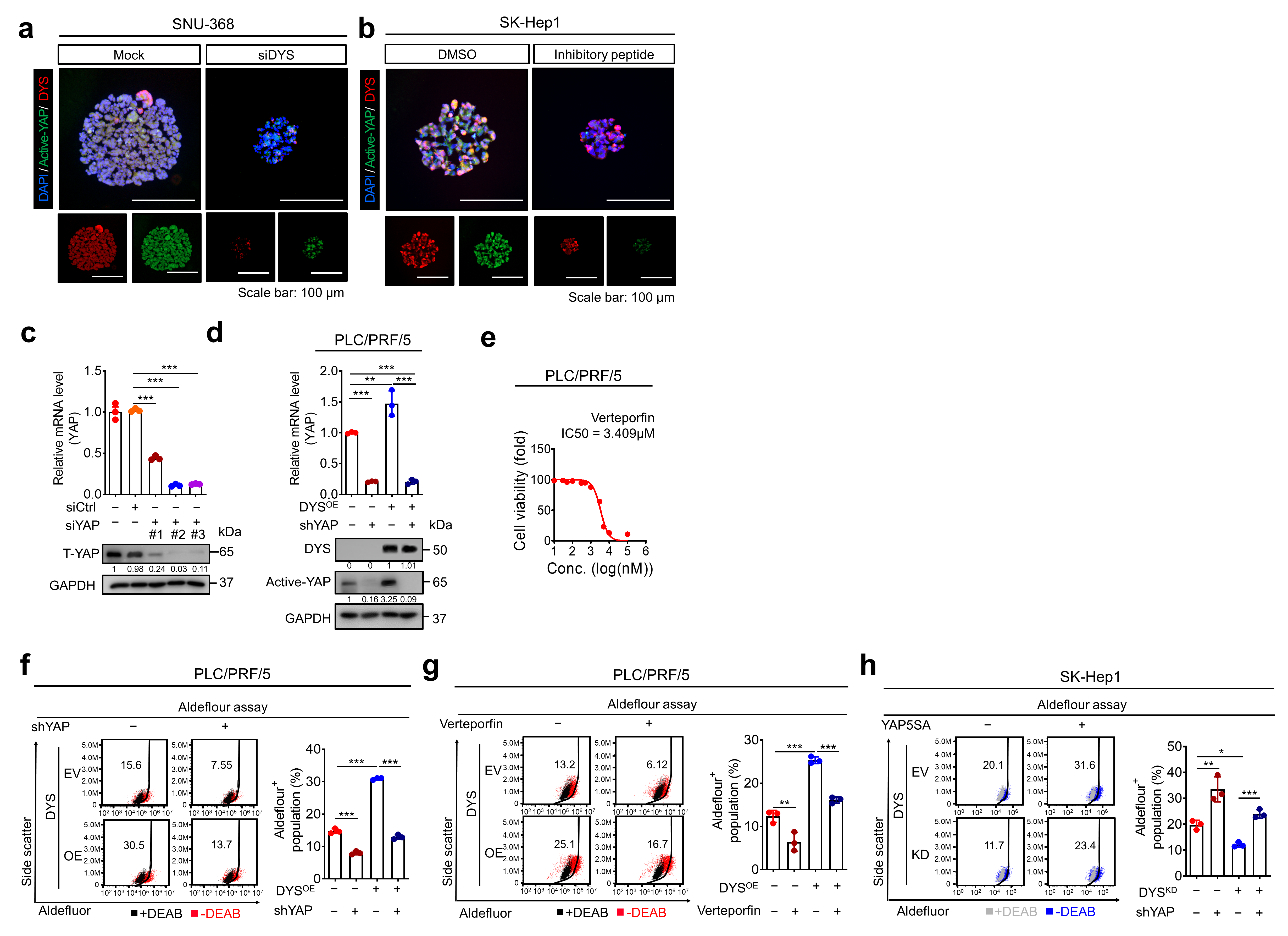


Figure. S3. Pharmacological and genetic inhibition of YAP suppresses dysadherin-induced CSC traits in HCC

**a, b** IF staining of active YAP in spheroid-forming SNU-368 and SK-Hep1 cells after dysadherin knockdown (siDYS) or inhibitory peptide treatment. Scale bar = 100 μm. **c, d** Validation of YAP silencing efficiency by siRNA (c) and shRNA (d) in PLC/PRF/5 cells, assessed by RT-qPCR and immunoblotting for total and active YAP. **e** IC_50_ determination of verteporfin (YAP-TEAD inhibitor) in PLC/PRF/5 cells using MTT viability assay. **f-h** Aldefluor assays measuring the CSC-like ALDH^+^ population in dysadherin OE or KD cells with or without YAP inhibition (shYAP or verteporfin) or rescue (YAP5SA). Representative flow cytometry plots and quantitative comparisons are shown for PLC/PRF/5 (f, g) and SK-Hep1 (h) cells. Data are presented as means ± SEM. Statistical significance was determined by unpaired two-tailed Student’s t-tests for comparisons between two groups, and one-way ANOVA with Dunnett’s multiple comparison test for comparisons among three or more groups. *p < 0.05, **p < 0.01, ***p < 0.001.


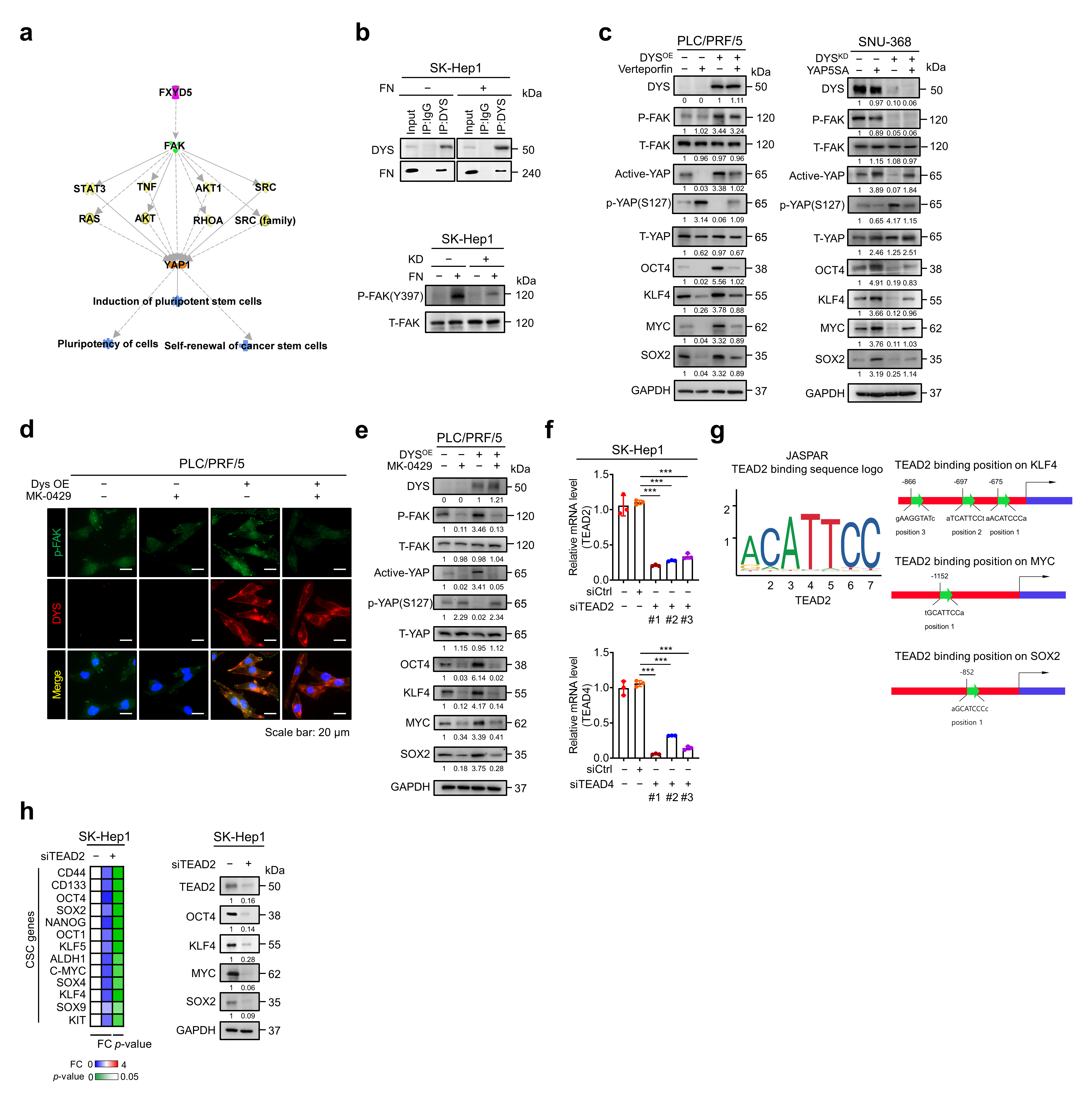


Figure. S4. The dysadherin–FAK–YAP axis regulates pluripotency through TEAD2-mediated transcription

**a** Ingenuity Pathway Analysis (IPA) network diagram illustrating the predicted signaling cascade linking dysadherin (FXYD5) to YAP via integrin–FAK–SRC/AKT signaling, ultimately promoting stem cell pluripotency programs. **b** Co-immunoprecipitation (Co-IP) assay showing the interaction between endogenous dysadherin and fibronectin in SK-Hep1 cells. Whole-cell lysates were immunoprecipitated (IP) with the indicated antibodies, followed by immunoblotting (IB). A non-specific IgG was used as a negative control. **c** Immunoblot analysis of FAK/YAP signaling components and pluripotency-related transcription factors (OCT4, KLF4, MYC, and SOX2) in dysadherin-OE PLC/PRF/5 cells treated with verteporfin (left) and dysadherin-KD SNU-368 cells treated with YAP5SA (right). **d** IF staining of dysadherin and phosphorylated FAK (p-FAK) in dysadherin-OE PLC/PRF/5 cells treated with or without MK-0429 (integrin inhibitor). Scale bar = 20 μm. **e** Immunoblot analysis showing suppression of FAK/YAP activation and downregulation of pluripotency transcription factors after MK-0429 treatment in dysadherin-OE PLC/PRF/5 cells. **f** RT-qPCR validation of TEAD2 and TEAD4 KD efficiency using three independent siRNAs per gene in SK-Hep1 cells. **g** JASPAR analysis showing the TEAD2 DNA-binding motif and predicted TEAD2 binding sites on the promoter regions of KLF4, MYC, and SOX2. **h**, Heatmap (left) and immunoblot analysis (right) of pluripotency-related genes in SK-Hep1 cells following transfection with control siRNA (siCTRL) or siRNA targeting TEAD2 (siTEAD2). Data are presented as means ± SEM. Statistical significance was determined by unpaired two-tailed Student’s t-tests for comparisons between two groups, and one-way ANOVA with Dunnett’s multiple comparison test for comparisons among three or more groups. *p < 0.05, **p < 0.01, ***p < 0.001.


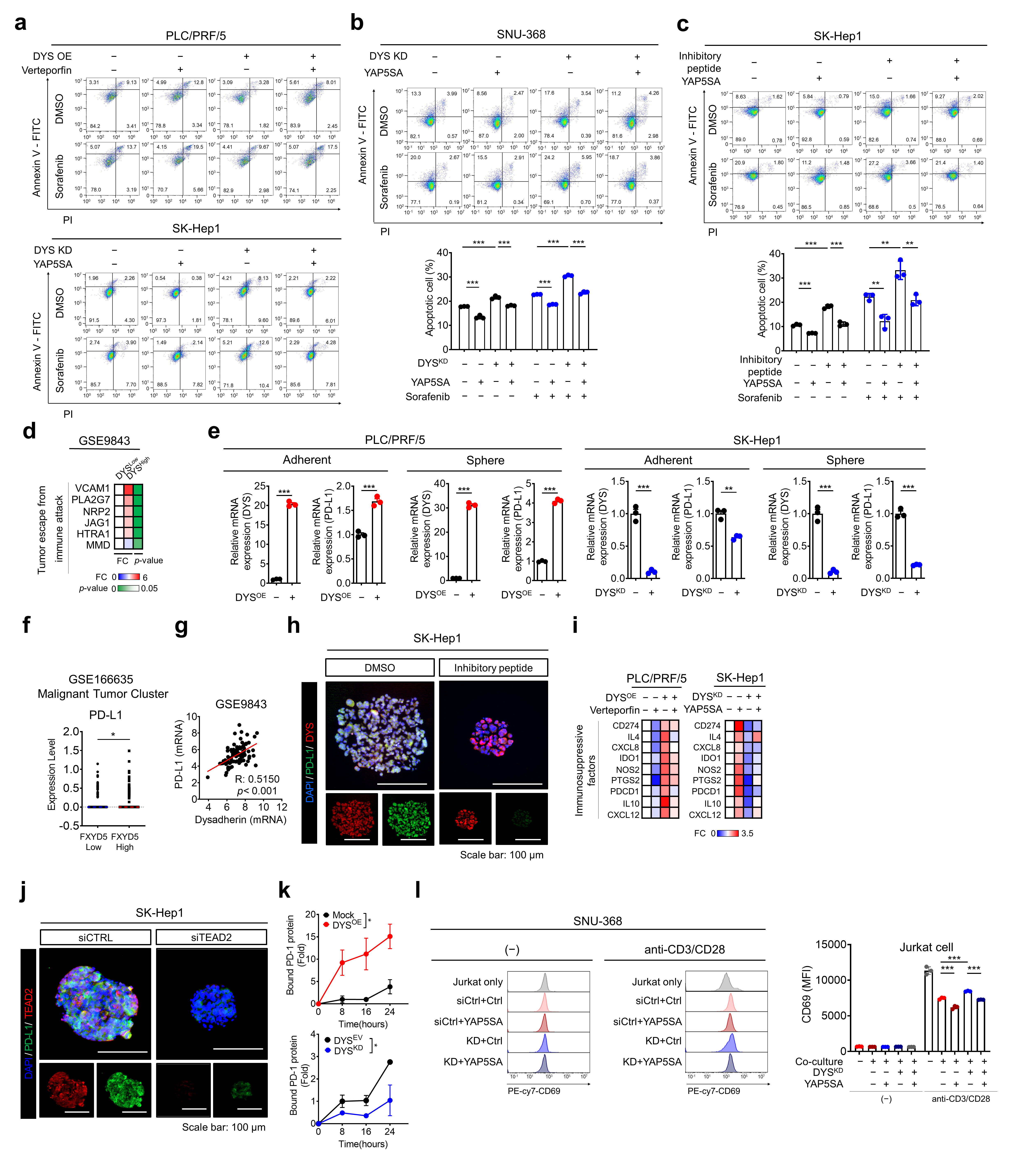


Figure. S5. Dysadherin promotes immune evasion and drug resistance via the YAP–PD-L1 axis in HCC

**a-c** Apoptosis assays using Annexin V/PI staining in PLC/PRF/5, SK-Hep1, and SNU-368 cells following treatment with sorafenib, verteporfin, or YAP5SA, in the context of dysadherin OE and KD, or pharmacologic inhibition. **d** Heatmap showing increased expression of immune escape–associated genes in dysadherin^high^ HCC tumors from the GSE9843 dataset. **e** RT-qPCR analysis of dysadherin (FXYD5) and PD-L1 (CD274) expression in adherent versus spheroid cultures of PLC/PRF/5 and SK-Hep1 cells. **f** Violin plot displaying PD-L1 expression levels in dysadherin^high^ versus dysadherin^low^ tumor clusters from GSE166635 single-cell RNA-seq data. **g** Scatter plot showing positive correlation between dysadherin and PD-L1 mRNA expression in bulk tumor samples (GSE9843). **h** IF staining showing co-localization of dysadherin and PD-L1 in SK-Hep1 spheroids with or without inhibitory peptide **i** Heatmap of immunosuppressive gene expression in dysadherin-modified PLC/PRF/5 and SK-Hep1 cells treated with verteporfin or YAP5SA. Scale bar = 100 μm. **j** IF staining showing co-localization of dysadherin and PD-L1 in SK-Hep1 spheroids with or without si TEAD2 treatment. Scale bar = 100 μm. **k** Time-course quantification of PD-L1/PD-1 binding in dysadherin OE or KD HCC cells using PD-1-Fc fusion protein staining and IF analysis. **l** T-cell activation assay using SNU-368 cells. CD69 expression on Jurkat T-cells was measured by flow cytometry after co-culture with SNU-368 cells that were subjected to dysadherin knockdown (siDYS), or rescue with YAP5SA as indicated. Representative histograms and quantification of CD69 mean fluorescence intensity are shown. Data are presented as means ± SEM. Statistical significance was determined by unpaired two-tailed Student’s t-tests for comparisons between two groups, and one-way ANOVA with Dunnett’s multiple comparison test for comparisons among three or more groups. *p < 0.05, **p < 0.01, ***p < 0.001.


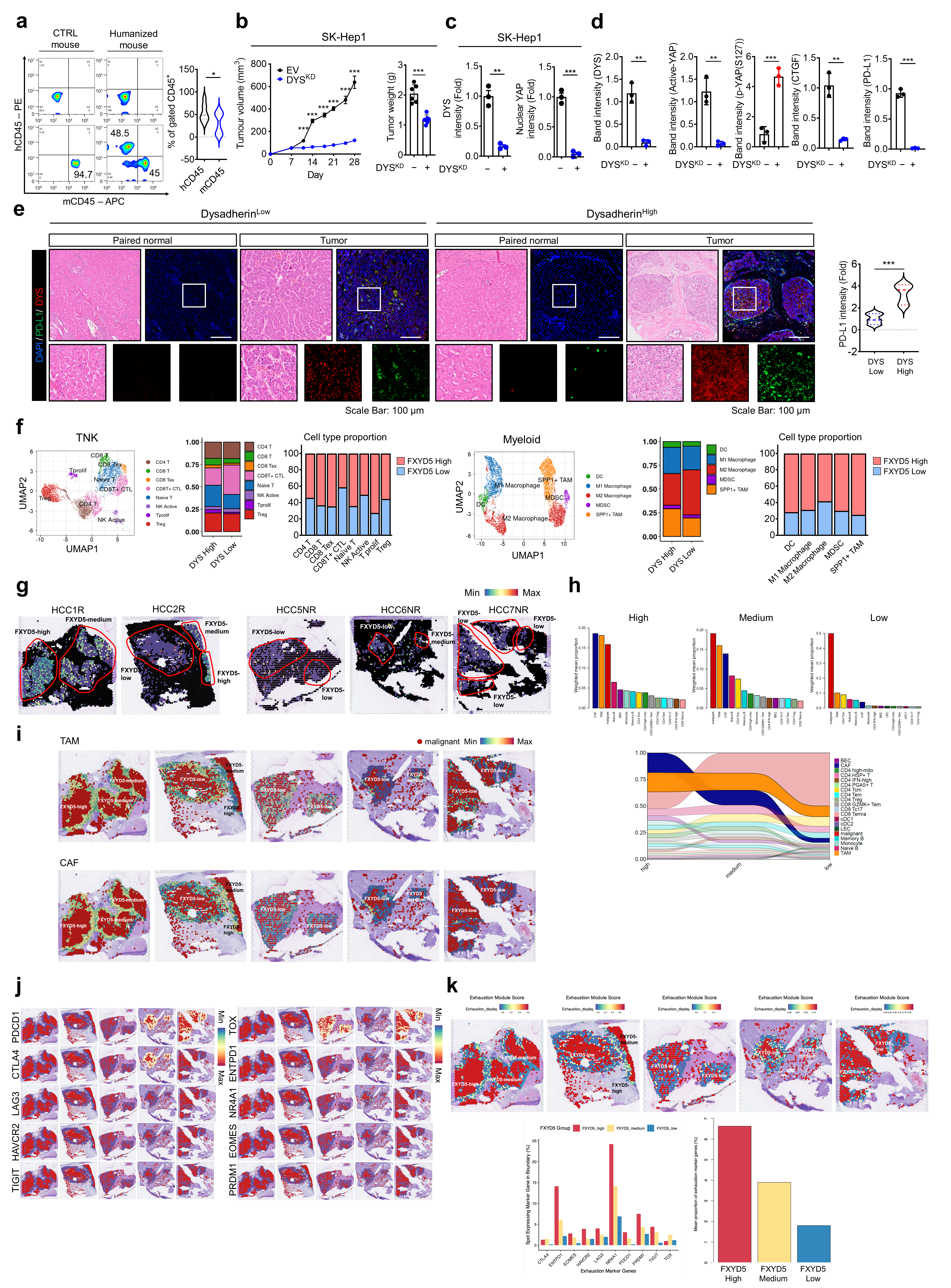


Figure. S6. Dysadherin expression is associated with an immunosuppressive and exhausted tumor microenvironment in HCC

**a** Flow cytometry analysis showing human CD45⁺ (hCD45⁺) versus mouse CD45⁺ (mCD45⁺) cell populations in peripheral blood, confirming successful human immune cell engraftment in humanized mice (n = 12). **b** Tumor growth curves and final tumor weight in SK-Hep1 xenografts with or without dysadherin knockdown (DYS^KD^). **c** Quantification of IF signal intensities for dysadherin and nuclear YAP in SK-Hep1 tumors from humanized mice. **d** Quantification of immunoblotting in Figure 6d. **e** Immunofluorescence analysis of dysadherin and PD-L1 in HCC patient tissues. (Left) Representative H&E and immunofluorescence images of paired normal and tumor tissues from patients with low and high dysadherin expression. Scale bar = 100 µm. (Right) Violin plot quantifying PD-L1 signal intensity in tumors stratified by dysadherin expression. **f** Single-cell RNA sequencing analysis of immune cells from HCC tumors, stratified by *FXYD5* expression. UMAP plots and cell type proportions are shown for T/NK cell subtypes (left) and myeloid cell subtypes (right). **g** Representative spatial transcriptomics plots of five HCC samples (GSE238264) showing heterogeneous *FXYD5* gene expression in malignant spots. **h** Quantification of cell types in *FXYD5*-defined regions. Bar charts show the weighted mean proportion of abundant cell types in high, medium, and low *FXYD5* expression regions (top). Alluvial plot shows the change in cell composition across these regions (bottom). **i** Spatial visualization showing the enrichment of Tumor-Associated Macrophages (TAMs, top) and Cancer-Associated Fibroblasts (CAFs, bottom) in *FXYD5*^High^ regions. **j** Spatial expression plots for a panel of individual T cell exhaustion marker genes across the five HCC samples. **k** Analysis of the composite immune exhaustion signature. Spatial plots show the Exhaustion Module Score (top). Bar charts quantify the expression of individual exhaustion marker genes (bottom left) and the mean proportion of these genes (bottom right) in regions stratified by *FXYD5* expression. Data are presented as means ± SEM. Statistical significance was determined by unpaired two-tailed Student’s t-tests for comparisons between two groups, and one-way ANOVA with Dunnett’s multiple comparison test for comparisons among three or more groups. *p < 0.05, **p < 0.01, ***p < 0.001.


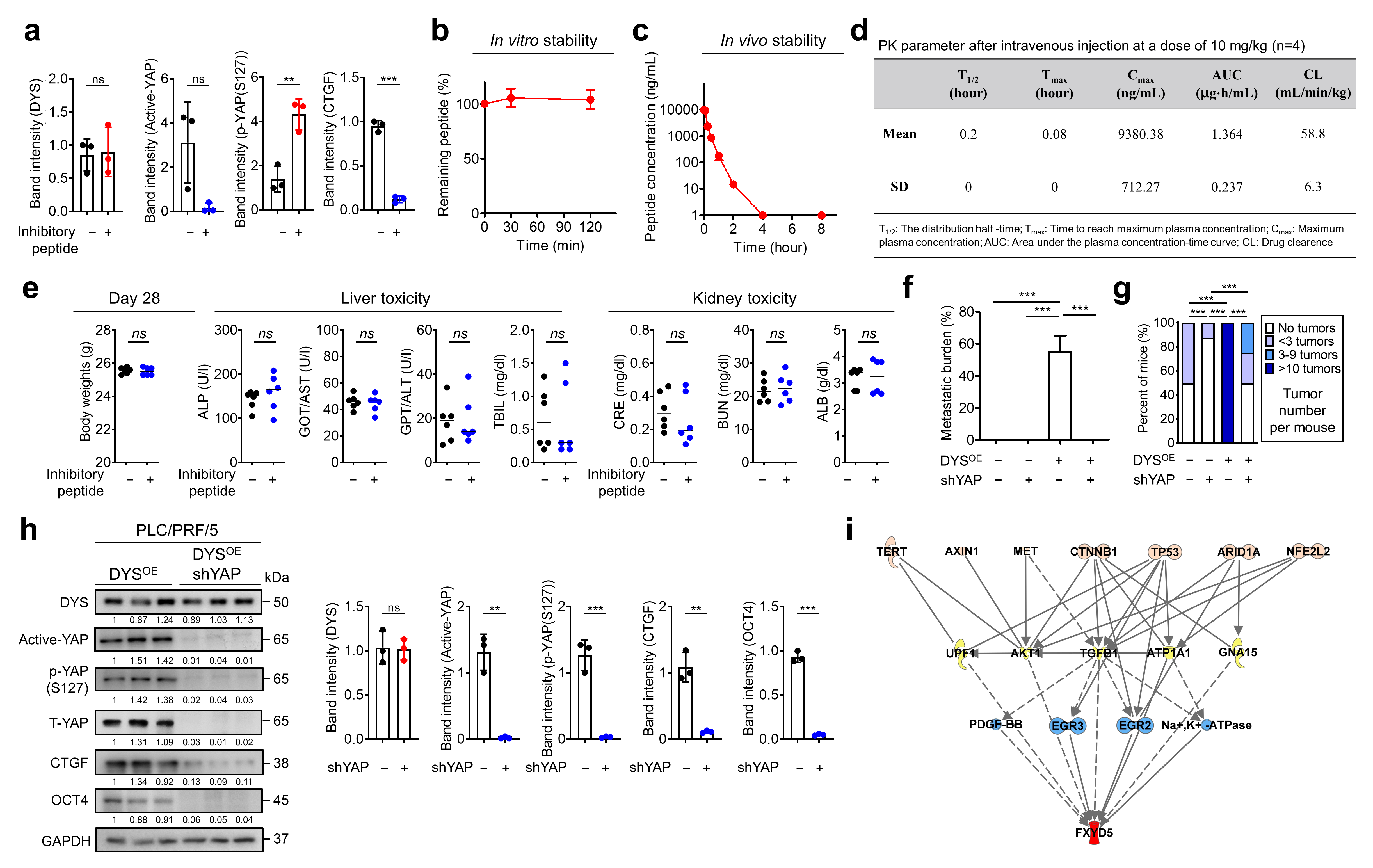


Figure. S7. In vivo efficacy, safety, and pharmacokinetics of dysadherin inhibition in HCC models

**a.** Quantification of immunoblotting in Figure 6j. **b,c** *In vitro* (b) and *in vivo* (c) plasma stability of the dysadherin-inhibitory peptide. Peptide concentrations were measured by LC-MS/MS at various time points post incubation (*in vitro*) or after intravenous injection (*in vivo*). **d** Pharmacokinetic parameters of the inhibitory peptide in mice, including elimination half-life (T₁/₂), time to peak concentration (T_max), maximum concentration (C_max), area under the curve (AUC), and clearance (CL). **e** Body weights and serum biomarkers assessing liver (ALP, GOT/AST, GPT/ALT, and TBIL) and kidney (CRE, BUN, ALB) toxicity in mice treated with the peptide versus vehicle control. **f** Quantification of metastatic burden in the liver following splenic injection of dysadherin-OE PLC/PRF/5 cells with or without YAP knockdown (shYAP). **g** Quantification of multiplicity per mouse in the liver metastasis model. **h** Immunoblot analysis of YAP, CTGF, and OCT4 expressions in liver metastatic nodules derived from dysadherin-OE or dysadherin-OE + shYAP cells. **i** An Ingenuity Pathway Analysis (IPA) network diagram showing predicted upstream regulators of FXYD5 in HCC development (CTNNB1, MET, TP53, and TERT). Data are presented as means ± SEM. Statistical significance was determined by unpaired two-tailed Student’s t-tests for comparisons between two groups, and one-way ANOVA with Dunnett’s multiple comparison test for comparisons among three or more groups. *p < 0.05, **p < 0.01, ***p < 0.001.

Table S1. Clinical information regarding the HCC patient samples used for IF staining (n = 14)

**- Clinical information**

| **# of Patients** | **Site** | **Sex** | **Age** | **Cell metaplasia** | | **PT Stage** | **Edmonson-Steiner Grade** |
| --- | --- | --- | --- | --- | --- | --- | --- |
| 07768562 | liver | F | 62 | Yes | T2 | | III |
| 07758425 | liver | M | 49 | Yes | T2 | | II |
| 07674610 | liver | M | 53 | Yes | T2 | | IV |
| 07669961 | liver | M | 60 | Yes | T2 | | III |
| 07636086 | liver | F | 70 | Yes | T1 | | IV |
| 07628731 | liver | M | 68 | Yes | T2 | | III |
| 07609920 | liver | M | 74 | Yes | T2 | | IV |
| 07575823 | liver | M | 48 | Yes | T2 | | III |
| 07478829 | liver | M | 65 | Yes | T3 | | IV |
| 07409390 | liver | M | 65 | Yes | T2 | | III |
| 07346723 | liver | F | 62 | Yes | T2 | | IV |
| 07237736 | liver | M | 69 | Yes | T2 | | III |
| 07163697 | liver | M | 62 | Yes | T2 | | IV |
| 07161975 | liver | M | 64 | Yes | T3 | | II |

Table S2. List of primers

| Target | Sequence | |
| --- | --- | --- |
|  | Forward | Reverse |
| Human  Dysadherin | TCCCACTGATGACACCACGA | AAACCAGATGGCTTGAGGGT |
| Human  YAP | TAGCCCTGCGTAGCCAGTTA | TCATGCTTAGTCCACTGTCTGT |
| Human  CD44 | GGAGCAGCACTTCAGGAGGTT | GGAATGTGTCTTGGTCTCTGGTAGC |
| Human  CD133 | CAGAGTACAACGCCAAACCA | AAATCACGATGAGGGTCAGC |
| Human  OCT4 | GGGCTCTCCCATGCATTCAAAC | CACCTTCCCTCCAACCAGTTGC |
| Human  SOX2 | TCGGCAGACTGATTCAAATA | CCATGCAGGTTGACACCGTT |
| Human  NANOG | TGGGATTTACAGGCGTGAGCCAC | AAGCAAAGCCTCCCAATCCCAAAC |
| Human  OCT1 | CCCTGTCTCAGCCCATACAGA | GCTGCAAATTGGTGGTTGGAT |
| Human  KLF5 | CCCTTGCACATACACAATGC | GGATGGAGGTGGGGTTAAAT |
| Human  ALDH1A1 | CAAATAGTGCACTGTCTCCAGG | ACGACACTACTTATTTGTAACACCT |
| Human  MYC | CAAGTATACGTGGCAATGCGT | TCAAGAGTCCCAGGGAGAG |
| Human  SOX4 | GGTCTCTAGTTCTTGCACGCTC | CGGAATCGGCACTAAGGAG |
| Human  KLF4 | ACGATCGTGGCCCCGGAAAAGGACC | CAACAACCGAAAATGCACCAGCCCCAG |
| Human  SOX9 | CATGAGCGAGGTGCACTCC | TCGCTTCAGGTCAGCCTTG |
| Human  PIK3CA | CATGCATTGTTTTGCACCCC | ATGGAAGACGGGAGATTCACAT |
| Human  CXCR2 | ATGCTGTTACGGATCCTGCC | CCCCATGTGGGCCTTAAACA |
| Human  CXCR4 | AGGAACCCTGTTTCCGTGAAG | GCAGCCTGTACTTGTCCGTC |
| Human  MMP9 | CGTTCAGGGAGATGCCCATT | TCGTAGTCAGCTGTTGTGCT |
| Human  MMP7 | TCGGAGGAGATGCTCACTTCGA | GGATCAGAGGAATGTCCCATACC |
| Human  TWIST | CCTGCGCAAGATCATCCCCA | GCTGCAGCTTGCCATCTTGGA |
| Human  SNAIL1 | CTGGGTGCCCTCAAGATGCA | CCGGACATGGCCTTGTAGCA |
| Human  SNAIL2 | TACCGCTGCTCCATTCCACG | CATGGGGGTCTGAAAGCTTGG |
| Human  ZEB1 | CCTACTCAGCCTCCTCCACTC | CCAGTAGCTGATGAAACAGAGGA |
| Human  IL18 | TTGACCAAGGAAATCGGCCT | GGTCCGGGGTGCATTATCTC |
| Human  VIM | ACCCGCACCAACGAGAAGGT | ATTCTGCTGCTCCAGGAAGCG |
| Human  TNS2 | AGCAGGACCTTTGGCATCTGCA | GACCAGACAACTCTGACGAAGC |
| Human  SERPINE1 | CTCATCAGCCACTGGAAAGGCA | GACTCGTGAAGTCAGCCTGAAAC |
| Human  CTGF | CCAATGACAACGCCTCCTG | TGGTGCAGCCAGAAAGCTC |
| Human  AXL | GTTTGGAGCTGTGATGGAAGGC | CGCTTCACTCAGGAAATCCTCC |
| Human  TNS1 | TCAAGTGGAAGAACTTGTTTGCTT | CACGACAATATAGTGGAGGCACA |
| Human  BIRC5 | AGCCCTTTCTCAAGGACCACC | TTGAAGCAGAAGAAACACTGGGC |
| Human  SGK1 | CGGAATGTTCTGTTGAAGAATGTG | TGTCAGCAGTCTGGAAAGAGAAGT |
| Human  TGM2 | AGAAGAGCGAAGGGACGTACTG | AGTCTACCACGTCGGCATTGAC |
| Human  CYR61 | GGAAAAGGCAGCTCACTGAAGC | GGAGATACCAGTTCCACAGGTC |
| Human  AMOTL2 | AGTGAGCGACAAACAGCAGACG | ATCTCTGCTCCCGTGTTTGGCA |
| Human  ETV5 | CAGTCAACTTCAAGAGGCTTGG | TGCTCATGGCTACAAGACGAC |
| Human  FLNA | CATCAAGTACGGTGGTGACG | ACATCCACCTCTGAGCCATC |
| Human  PPIA | TGCCATCGCCAAGGAGTAG | TGCACAGACGGTCACTCAAA |
| Mouse  Dysadherin | GAAAGGTACCCCTGCAGTCT | ACCAGCAGTCCCCGTTTC |
| Mouse GAPDH | ATGTGTCCGTCGTGGATCTGA | TTGAAGTCGCAGGAGACAACC |
| Mouse  CD44 | TCGATTTGAATGTAACCTGCCG | CAGTCCGGGAGATACTGTAGC |
| Mouse  CD133 | GTTGAGACTGTGCCCATGAAA | GACGGGCTTGTCATAACAGGA |
| Mouse  OCT4 | CGGAAGAGAAAGCGAACTAGC | ATTGGCGATGTGAGTGATCTG |
| Mouse  SOX2 | GCGGAGTGGAAACTTTTGTCC | CGGGAAGCGTGTACTTATCCTT |
| Mouse  NANOG | CACAGTTTGCCTAGTTCTGAGG | GCAAGAATAGTTCTCGGGATGAA |
| Mouse  OCT1 | AGCTGGGACAAGTTTACAGGC | TCCCGACTCTTCACTGGATTTA |
| Mouse  KLF5 | CCGGAGACGATCTGAAACACG | GTTGATGCTGTAAGGTATGCCT |
| Mouse  ALDH1A1 | ATACTTGTCGGATTTAGGAGGCT | GGGCCTATCTTCCAAATGAACA |
| Mouse  MYC | ATGCCCCTCAACGTGAACTTC | CGCAACATAGGATGGAGAGCA |
| Mouse  SOX4 | GACCTGCTCGACCTGAACC | ACTCCAGCCAATCTCCCGA |
| Mouse  KLF4 | GTGCCCCGACTAACCGTTG | GTCGTTGAACTCCTCGGTCT |
| Mouse  SOX9 | GAGCCGGATCTGAAGAGGGA | GCTTGACGTGTGGCTTGTTC |
| Mouse  PIK3CA | CACCTGAACAGACAAGTAGAGGC | GCAAAGCATCCATGAAGTCTGGC |
| Mouse  CXCR2 | CTCTATTCTGCCAGATGCTGTCC | ACAAGGCTCAGCAGAGTCACCA |
| Mouse  CXCR4 | GACTGGCATAGTCGGCAATGGA | CAAAGAGGAGGTCAGCCACTGA |
| Mouse  MMP9 | TAGTGAGAGACTCTACACAG | CCACTTCTTGTCAGTGTCGA |
| Mouse  MMP7 | AGGTGTGGAGTGCCAGATGTTG | CCACTACGATCCGAGGTAAGTC |
| Mouse  TWIST | GATTCAGACCCTCAAACTGGCG | AGACGGAGAAGGCGTAGCTGAG |
| Mouse  SNAIL1 | ATTCTCCTGCTCCCACTGC | GACTCTTGGTGCTTGTGGAG |
| Mouse  SNAIL2 | CTCACCTCGGGAGCATACAG | GACTTACACGCCCCAAGGATG |
| Mouse  ZEB1 | ATTCAGCTACTGTGAGCCCTGC | CATTCTGGTCCTCCACAGTGGA |
| Mouse  IL18 | GACAGCCTGTGTTCGAGGATATG | TGTTCTTACAGGAGAGGGTAGAC |
| Mouse  VIM | CGGAAAGTGGAATCCTTGCAGG | AGCAGTGAGGTCAGGCTTGGAA |
| Mouse  TNS2 | CTCAACAGGACCCTTGGCTTCT | GACAGCTCTGAAGAAGCACTGC |
| Mouse  SERPINE1 | CCTCTTCCACAAGTCTGATGGC | GCAGTTCCACAACGTCATACTCG |
| Mouse  CTGF | GGGCCTCTTCTGCGATTTC | ATCCAGGCAAGTGCATTGGTA |
| Mouse  AXL | ATGGCCGACATTGCCAGTG | CGGTAGTAATCCCCGTTGTAGA |
| Mouse  TNS1 | CTGGTGTATGTCACCGAACG | GTTCAGAGAGGTTGAATAGCAGG |
| Mouse  BIRC5 | CCTACCGAGAACGAGCCTGATT | CCATCTGCTTCTTGACAGTGAGG |
| Mouse  SGK1 | CTCATTCCAGACCGCTGACAAAC | CCAAGGCACTGGCTATTTCAGC |
| Mouse  TGM2 | GAAGGAACACGGCTGTCAGCAA | GATGAGCAGGTTGCTGTTCTGG |
| Mouse  CYR61 | GTGAAGTGCGTCCTTGTGGACA | CTTGACACTGGAGCATCCTGCA |
| Mouse  AMOTL2 | CAGAGGGACAATGAGCGATTGC | TCACGCTTGGAAGAGGTCCTCA |
| Mouse  ETV5 | CAAGTCCCTTTTATGGTCCCAG | ACTCTTCAGAATCGTGAGCCA |
| Mouse  FLNA | CAGCAAGCTACAGGTGGAACCT | TCAGTGGTTGCCTCTCGGAAGA |
| OCT4  Promoter  ChIP#1 | AGGTGTGGCCAGGCACTTT | CTCCCAGGTTCAAGCGATTCT |
| OCT4  Promoter  ChIP#2 | TCGCTTGAACCTGGGAGATG | GCTGGTCAGGAGACCTCAAGTG |
| OCT4  Promoter  ChIP#3 | AAAATTAGCCGGGCATGGT | GCGAAATGATCTCTCACCCTTT |
| OCT4  Promoter  ChIP#4 | GTGAGAGATCATTTCGCTTGGA | GGGTAAAGGAGGGAAGGAGATT |
| OCT4  Promoter  ChIP#5 | CCTTCCCTCCTTTACCCTACTC | CCCCTATAATCCCAGCTACTCA |
| OCT4  Promoter  ChIP#6 | GTTGACCAGGCTAGTCTTGAACT | CCACCCCTGCTGCCTCTATTTA |
| OCT4  Promoter  ChIP#7 | CTATGTTACCCAGGCTGCTT | TTCCCTGCCCTATAACTTTT |
| PD-L1  Promoter  ChIP#1 | GCTGGGCCCAAACCCTATTG | AGCCCTTGCATTGGTTATGACA |
| PD-L1  Promoter  ChIP#2 | ACAACGAAGAGTCCAATTTCTCAA | CCAAGATGACAGACGATGGTG |
| PD-L1  Promoter  ChIP#3 | ACCATCGTCTGTCATCTTGGG | TCCCATCCCGAGCTACATCT |
| PD-L1  Promoter  ChIP#4 | GATGTAGCTCGGGATGGGAA | ACAACATATAAAAAGTCAGCAGCAG |
| PD-L1  Promoter  ChIP#5 | AAAGGGGGACGCCTTTCTG | ACATGTCAGTCCAGTTTTCTTGT |
| PD-L1  Promoter  ChIP#6 | TCTTCGAAACTCTTCCCGGT | TGGACTTTCCTGACCTTCGG |

Table S3. List of antibodies

| **Immunoblot analysis** | | | | |
| --- | --- | --- | --- | --- |
| Target | Conjugate | | Catalog# (company or provider) | |
| Human Dysadherin | - | | M53/provided by Dr. Ino | |
| Human YAP (Active) | - | | ab205270/Abcam | |
| Human p-YAP (S127) | - | | ab76252/Abcam | |
| Human YAP | - | | 14074/Cell Signaling Technology | |
| Human CTGF | - | | 86641/Cell Signaling Technology | |
| Human GAPDH | - | | 5174/Cell Signaling Technology | |
| Human OCT4 | - | | 75463/Cell Signaling Technology | |
| Human KLF4 |  | | ab72543/Abcam | |
| Human MYC |  | | ab32072/Abcam | |
| Human SOX2 |  | | ab184149/Abcam | |
| Human PD-L1 |  | | 13684/Cell Signaling Technology | |
| Human p-FAK | - | | 3283/Cell Signaling Technology | |
| Human t-FAK | - | | 3285/Cell Signaling Technology | |
| Mouse YAP (Active) | - | | ab205270/Abcam | |
| Mouse p-YAP (S127) | - | | ab76252/Abcam | |
| Mouse YAP | - | | 14074/Cell Signaling Technology | |
| Mouse CTGF | - | | 86641/Cell Signaling Technology | |
| Goat anti-mouse | HRP | | 554002/BD Pharmingen™ | |
| Goat anti-rabbit | HRP | | 554021/BD Pharmingen™ | |
| **IF staining** | | | | |
| Target | | Conjugate | | Catalog# (company or provider) |
| Human Dysadherin | | - | | M53/provided by Dr. Ino |
| Human YAP | | - | | 14074/Cell Signaling Technology |
| Human YAP (Active) | | - | | ab205270/Abcam |
| Human p-FAK | | - | | ab81298/ Abcam |
| Human PD-L1 | | - | | 13684/Cell Signaling Technology |
| Mouse Dysadherin | | - | | sc377163/Santa Cruz Biotechnology |
| Goat anti-mouse | | Alexa 555 | | A21422/ThermoFisher |
| Donkey anti-rabbit | | Alexa 488 | | A21206/ThermoFisher |

| **ChIP assay** | | |
| --- | --- | --- |
| Target | Conjugate | Catalog# (company or provider) |
| Human TEAD2 | - | 8248060/MyBioScource |
| Human YAP | - | H00010413-M01/Abnova |

Table S4. List of siRNAs

| Target | | | Sequence | |  |
| --- | --- | --- | --- | --- | --- |
|  |  |  | Sense | | Anti-sense |
| Dysadherin |  | GUGUCUUCUCACCAUCGUU | | AACGAUGGUGAGAAGACAC |  |
| YAP | #1 | CAGAAGAUCAAAGCUACUU | | AAGUAGCUUUGAUCUUCUG |  |
|  | #2 | AGAACCGUUUCCCAGACUA | | UAGUCUGGGAAACGGUUCU |  |
|  | #3 | CAGGAAUUGAGAACAAUGA | | UCAUUGUUCUCAAUUCCUG |  |
| TEAD2 | #1 | CUCCAUGAUCUGUGAGAUC | | GAUCUCACAGGUCAUGGAG |  |
|  | #2 | CCCGAAGGAAAUCAAGGGA | | UCCCUUGAUUUCCUUCGGG |  |
|  | #3 | GGGCCCAGCAUCACAUUUA | | UAAAUGUGAUGCUGGGCCC |  |
| TEAD4 | #1 | GACACUACUCUUACCGCAU | | AUGCGGUAAGAGUAGUGUC |  |
|  | #2 | CAGAGUAUGCUCGCUAUGA | | UCAUAGCGAGCAUACUCUG |  |
|  | #3 | GGAGACCUUGCUGUGCAUU | | AAUGCACAGCAAGGUCUCC |  |
