# Supplementary material for: Dysadherin/YAP axis fuels stem plasticity and immune escape in liver cancer
Source: Signal Transduct Target Ther. 2025 Dec 29;10:421. doi: 10.1038/s41392-025-02520-4 (PMC12745361; doi:10.1038/s41392-025-02520-4)

Figure 1f

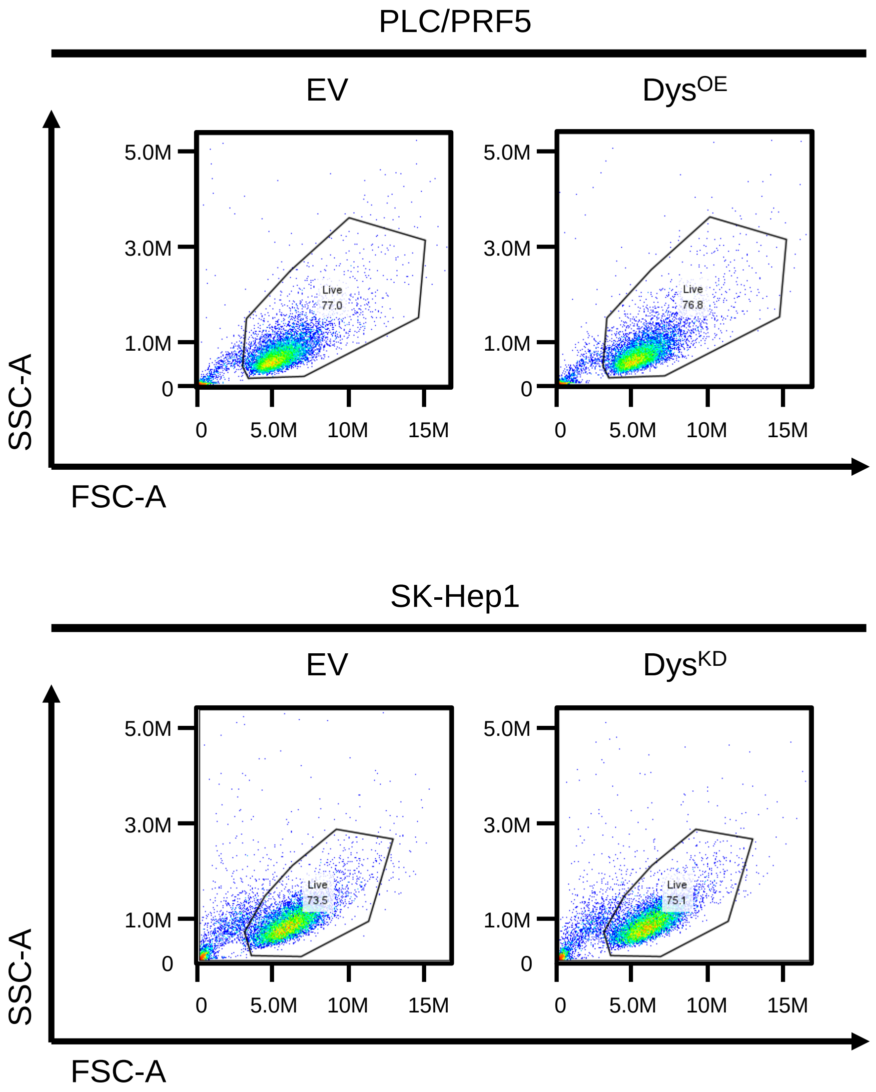
PLC/PRF5
EV
Dys^{OE}
SSC-A
FSC-A
5.0M
3.0M
1.0M
0
5.0M
10M
15M
Live
77.0
Live
76.8
SK-Hep1
EV
Dys^{KD}
Live
73.5
Live
75.1

Supplementary Figure 2f

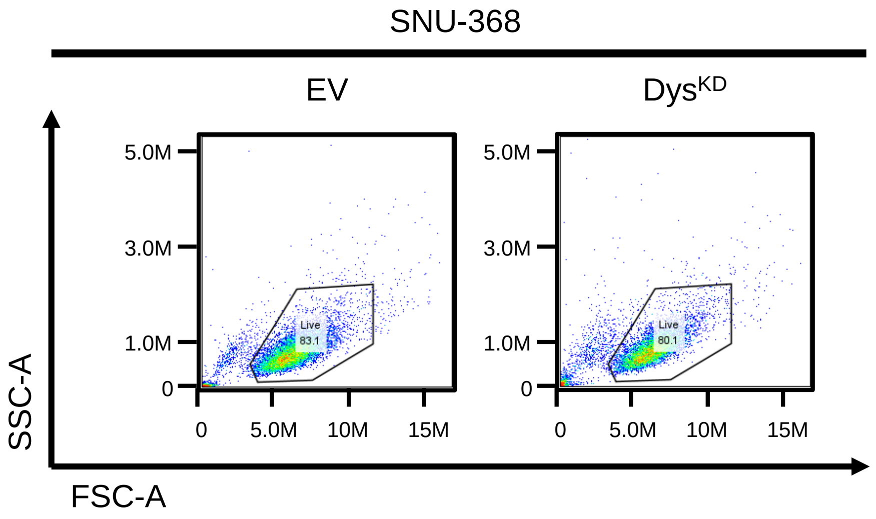

Supplementary Figure 2k

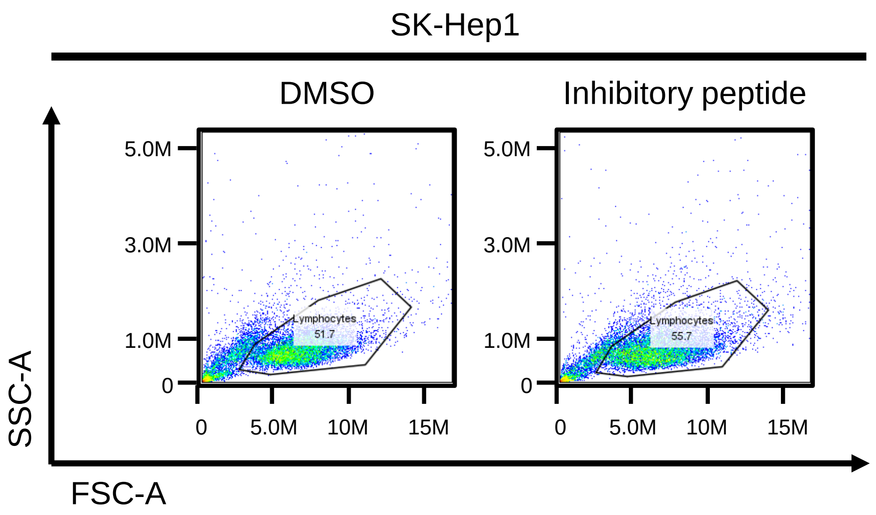

Supplementary Figure 3f

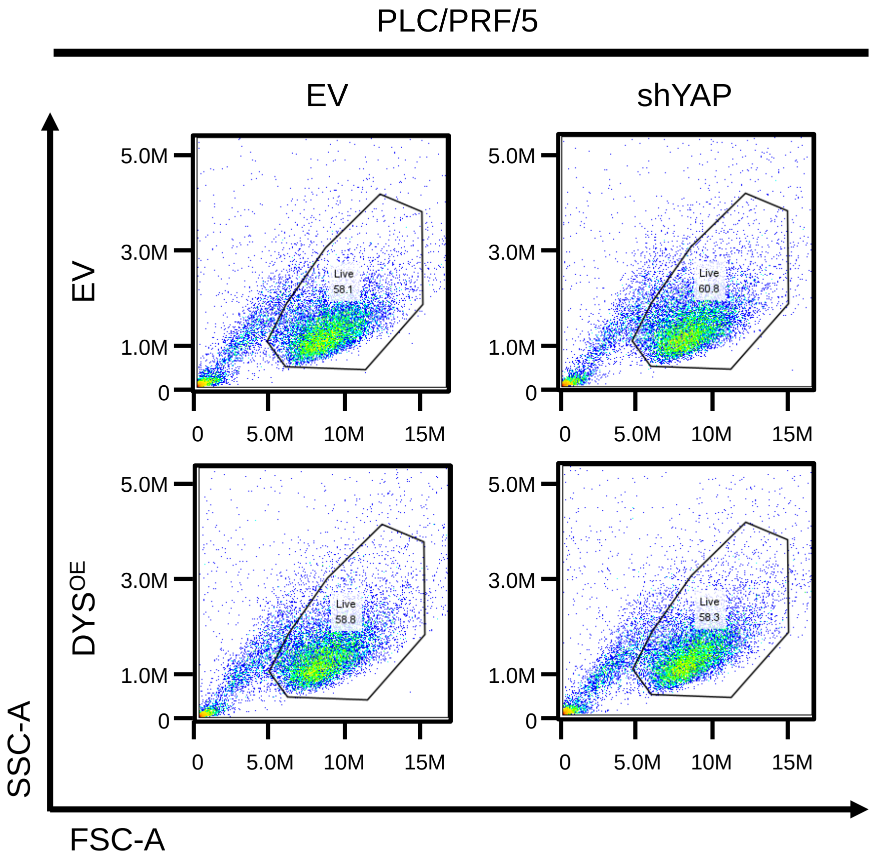

Supplementary Figure 3g

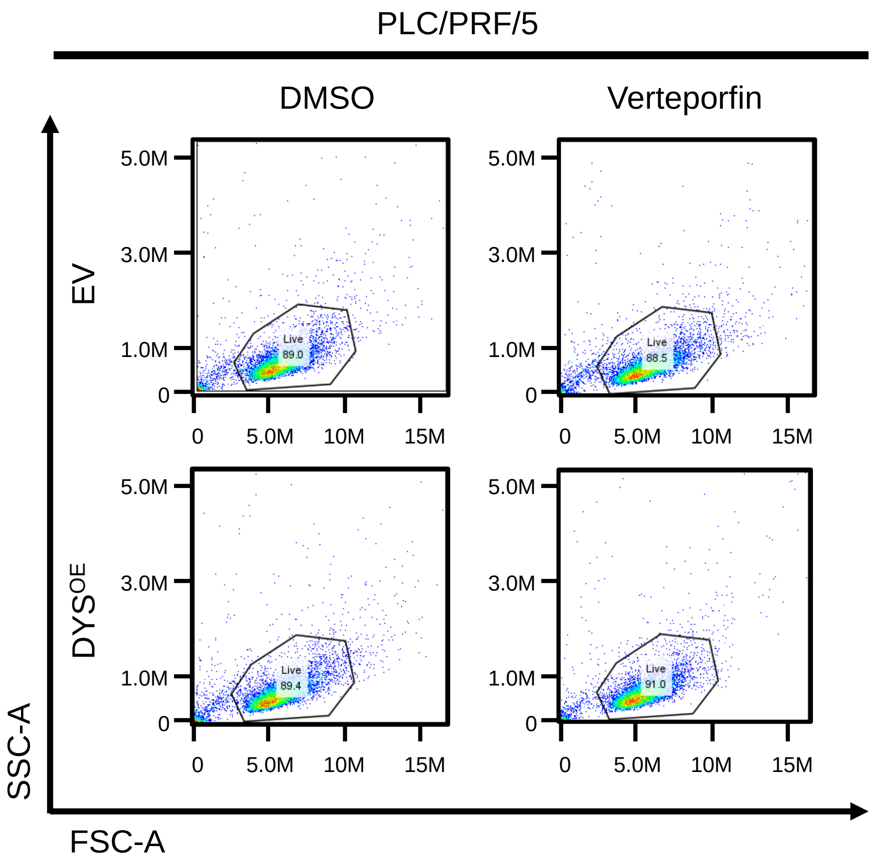

Supplementary Figure 3h

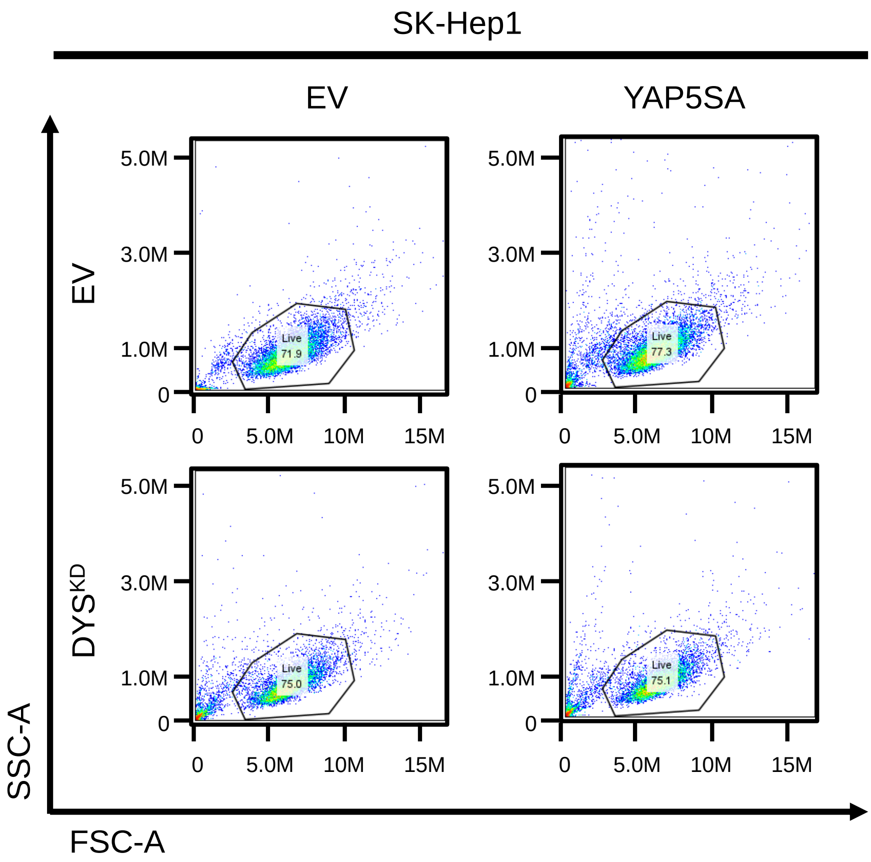

Supplementary Figure 5a

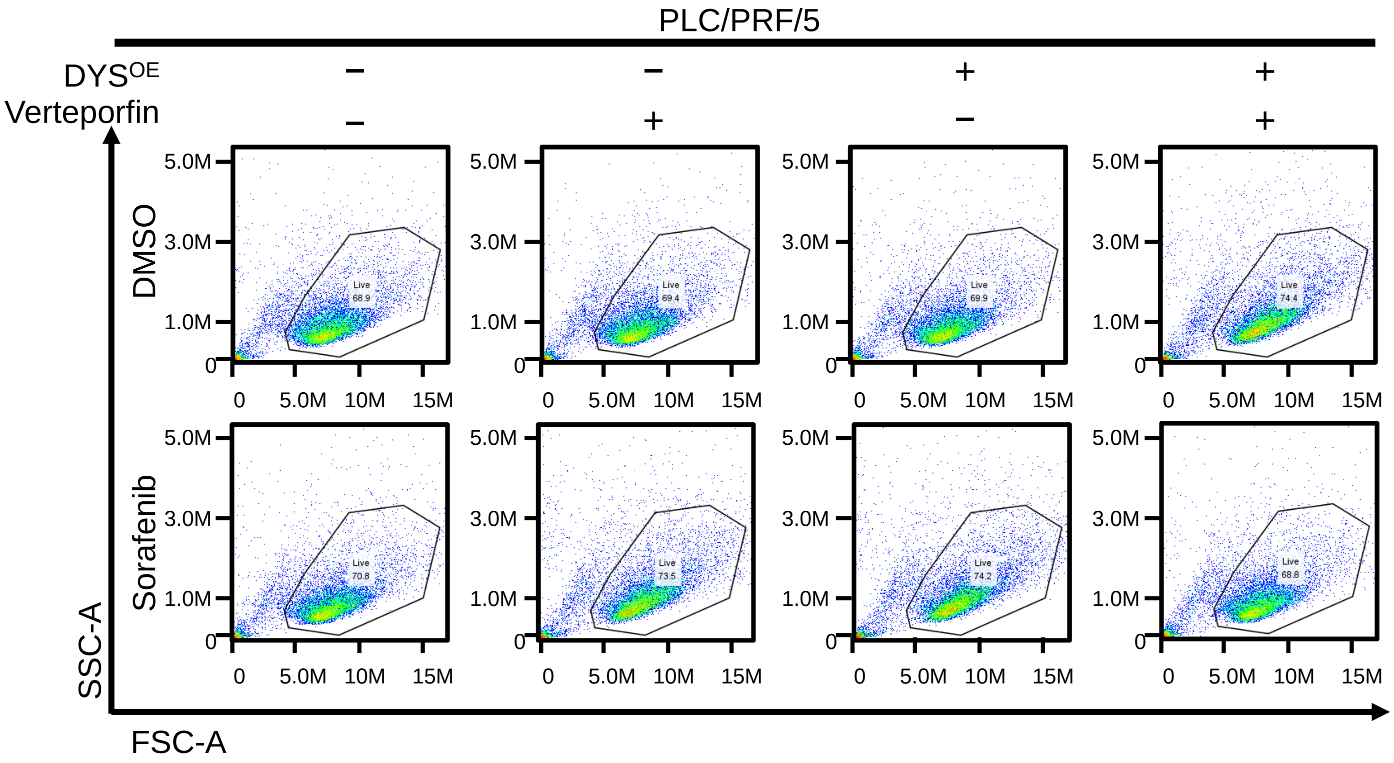

Supplementary Figure 5a

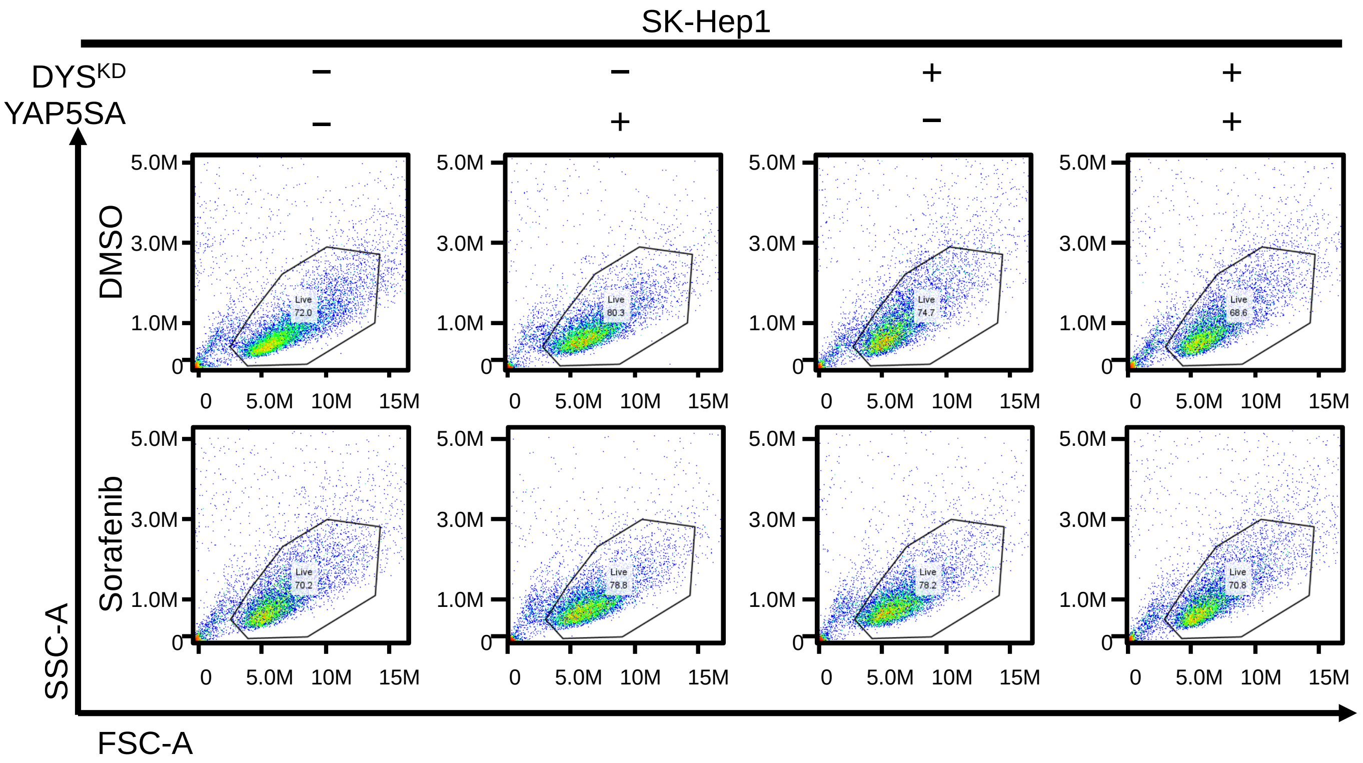

Supplementary Figure 5b

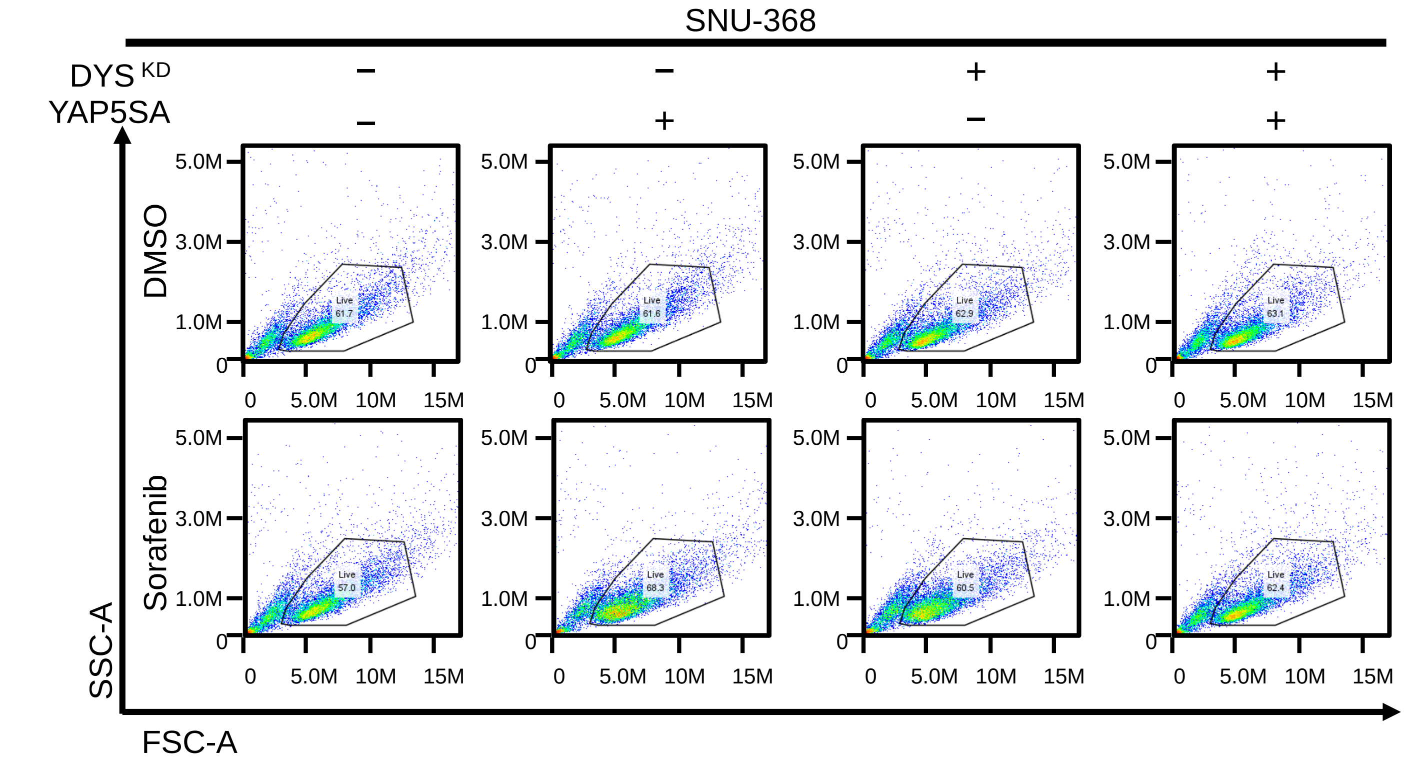

Supplementary Figure 5c

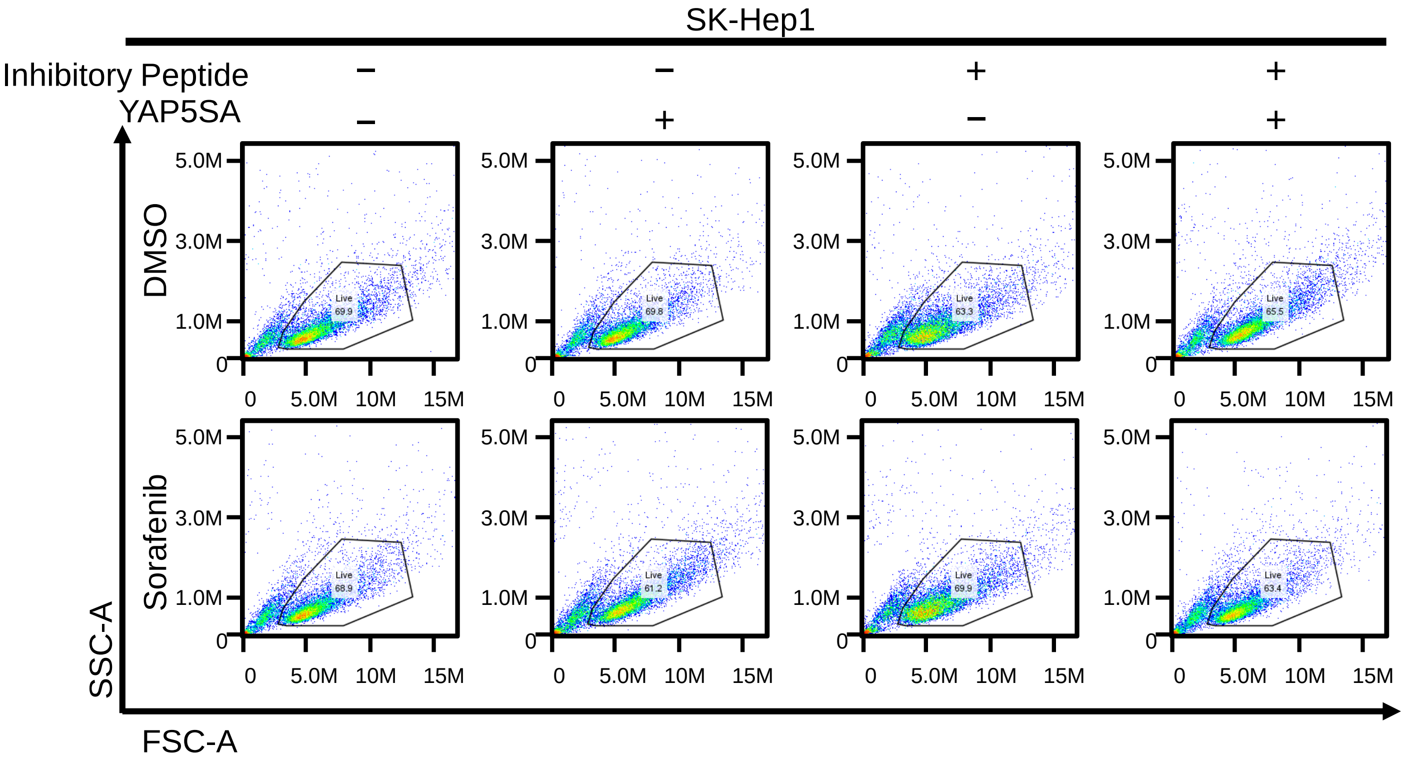

Figure 5i

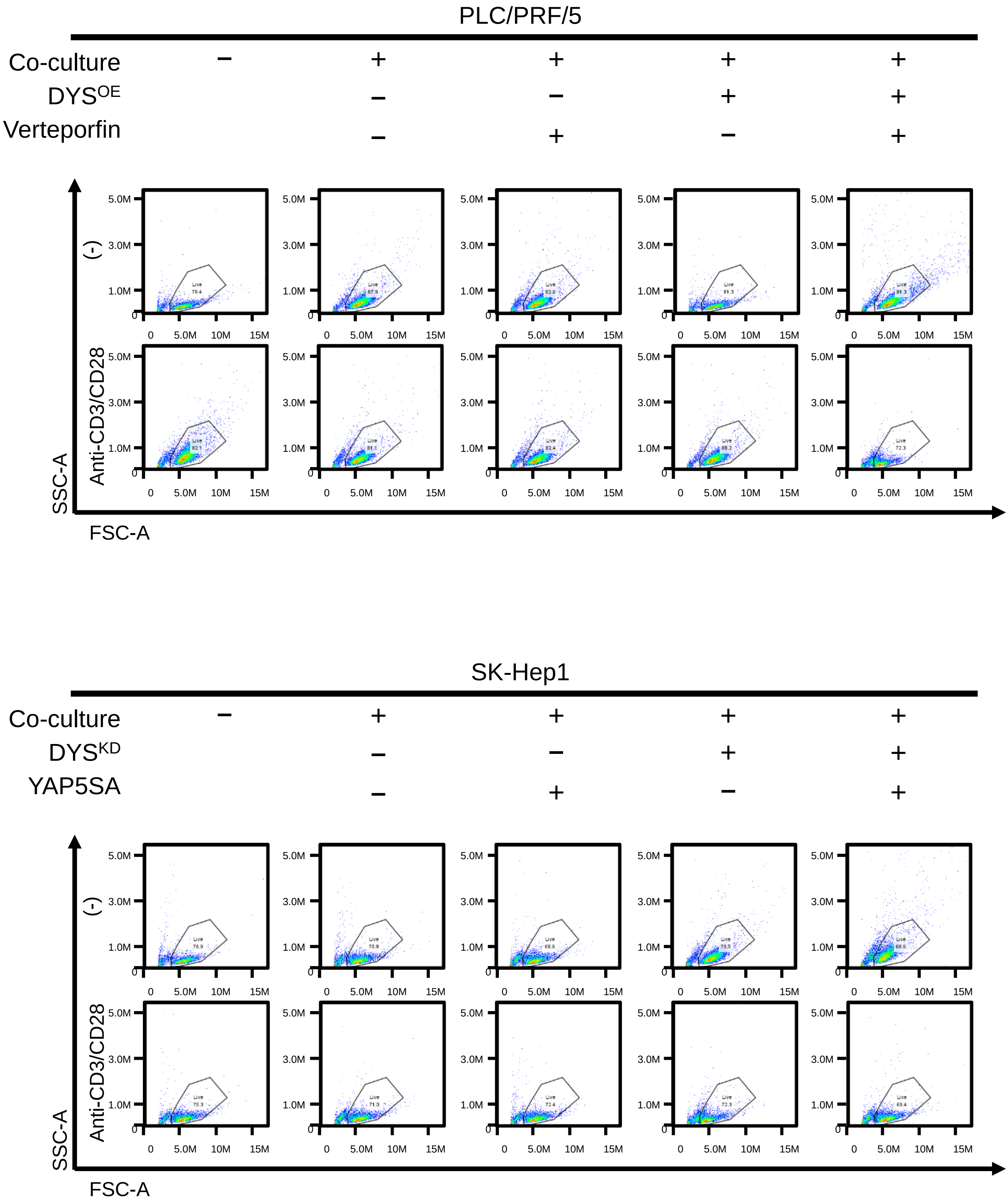
PLC/PRF/5
Co-culture − + + + +
DYS^OE − − + +
Verteporfin − + − +
(-)
Anti-CD3/CD28
SSC-A
FSC-A
5.0M
3.0M
1.0M
0
5.0M
10M
15M
SK-Hep1
Co-culture − + + + +
DYS^KD − − + +
YAP5SA − + − +
(-)
Anti-CD3/CD28
SSC-A
FSC-A

Supplementary Figure 5l

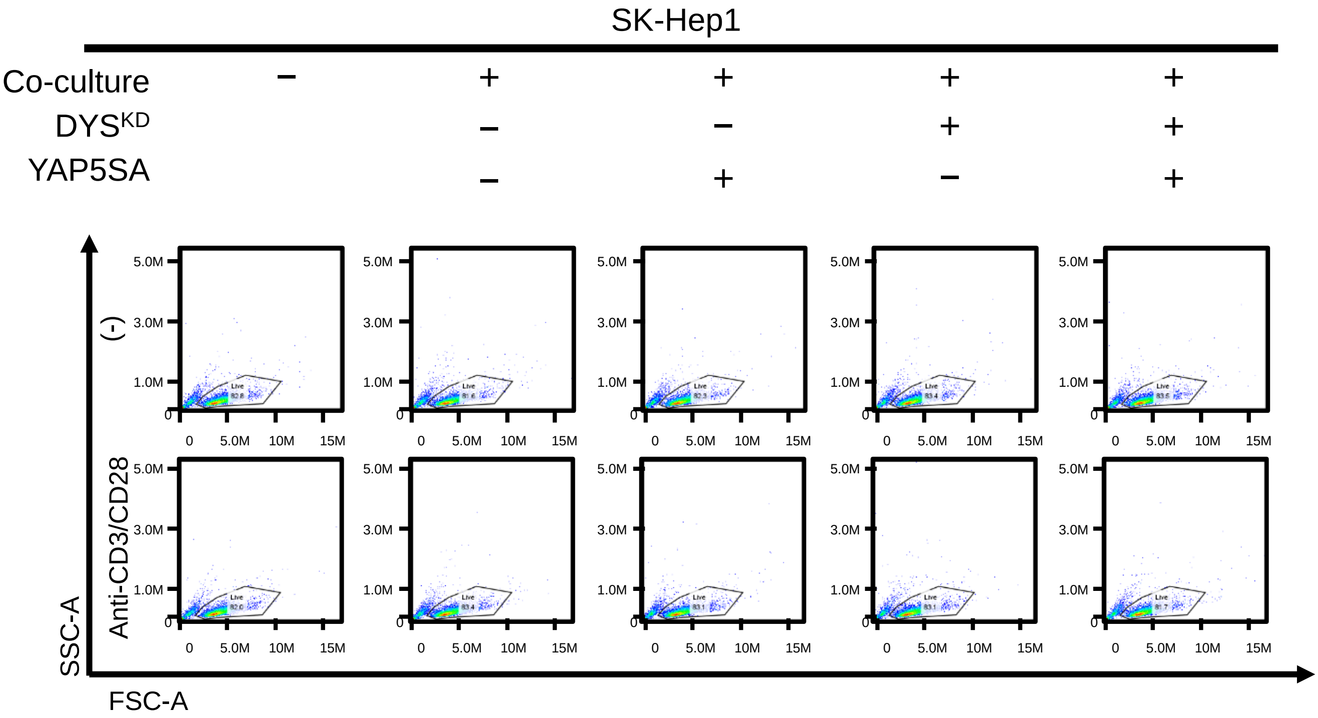

Supplementary Figure 6a

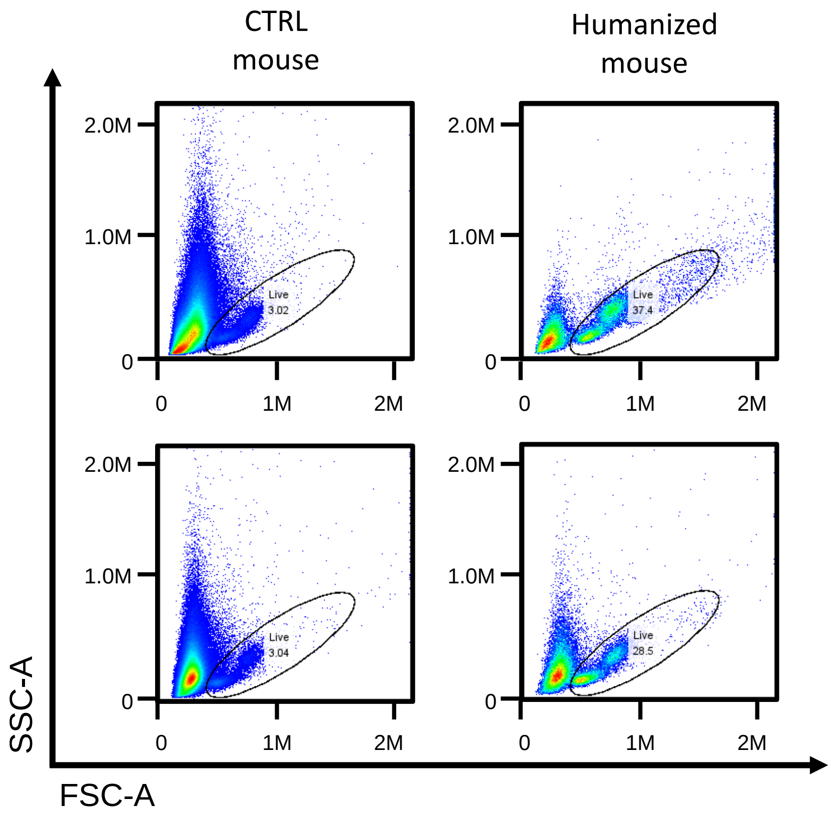

Supplement: Supplementary file 2 — Supplementary Information [file 41392_2025_2520_MOESM2_ESM.pdf]
