# Supplementary material for: Dysadherin/YAP axis fuels stem plasticity and immune escape in liver cancer
Source: Signal Transduct Target Ther. 2025 Dec 29;10:421. doi: 10.1038/s41392-025-02520-4 (PMC12745361; doi:10.1038/s41392-025-02520-4)

Supplementary Figure 1b

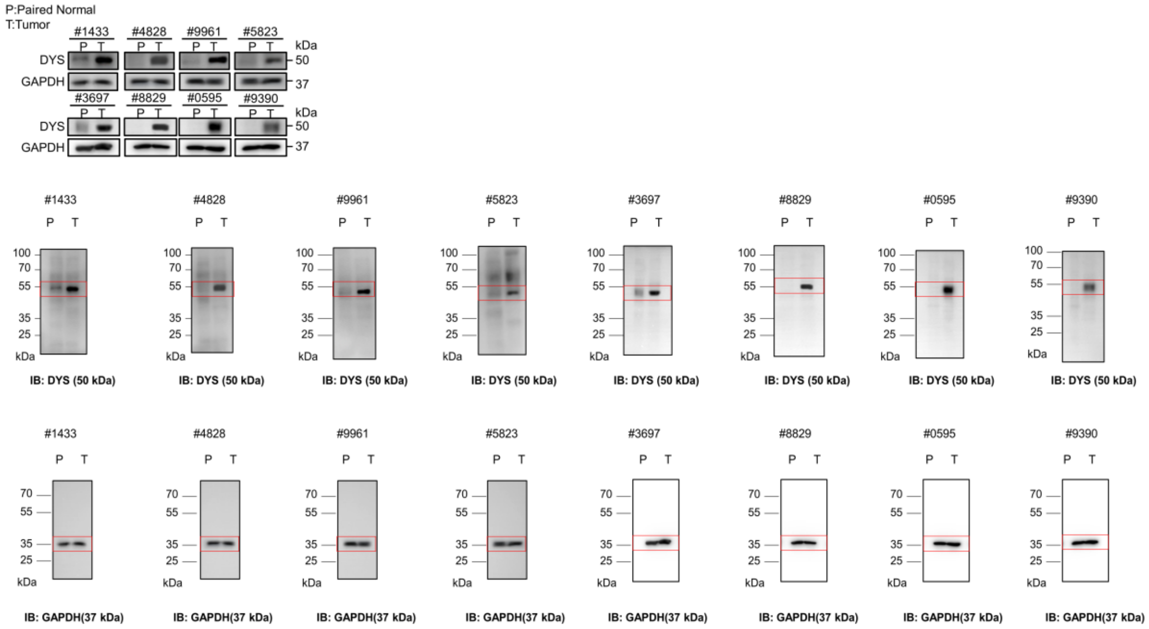

Supplementary Figure 1g

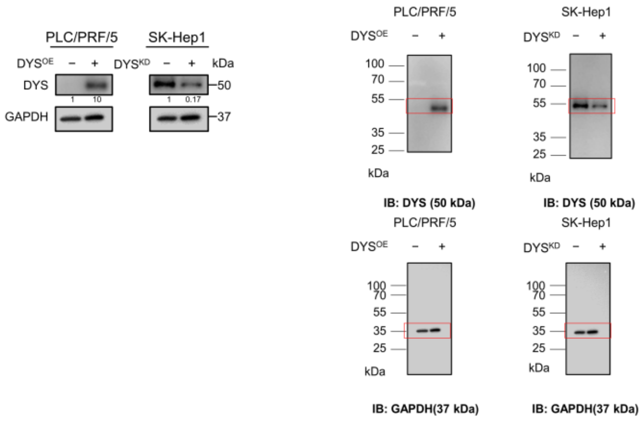

Figure 2f

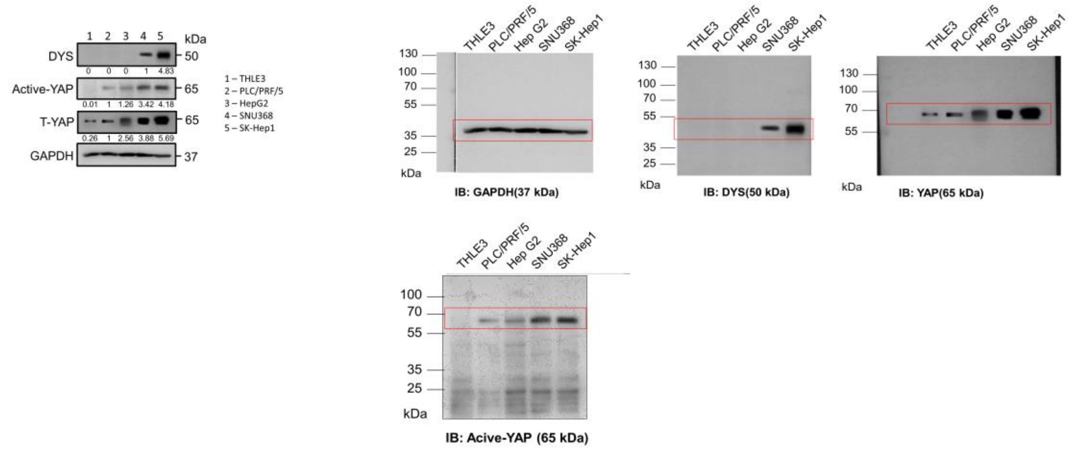

Figure 2h

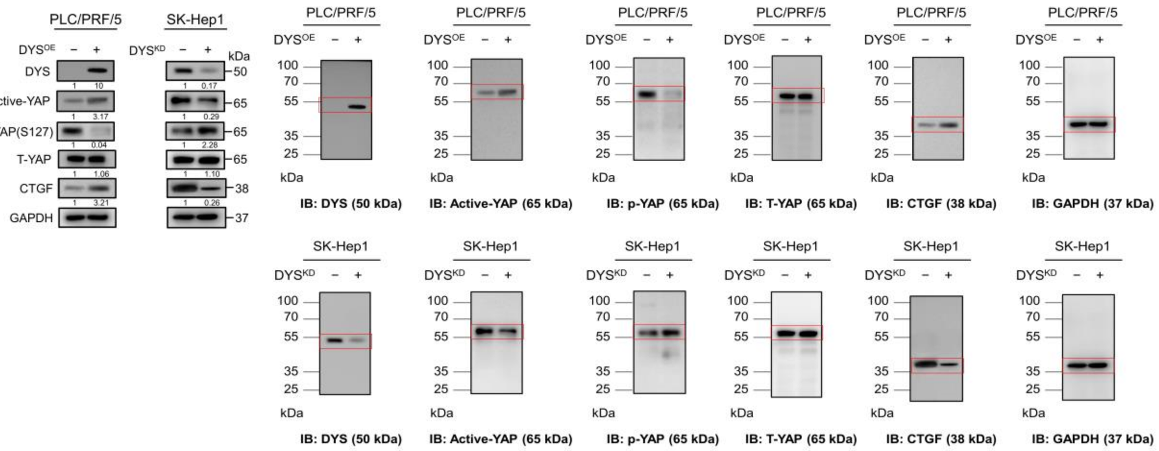

Figure 2j

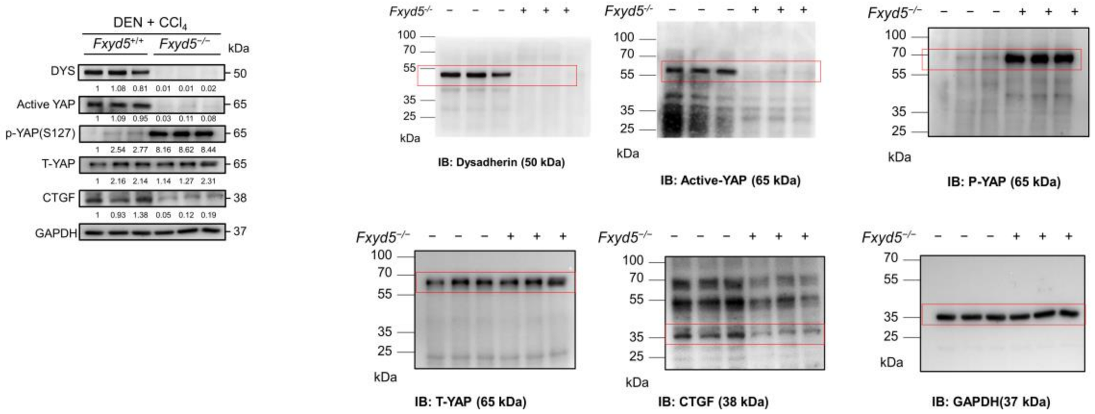

Supplementary Figure 2i

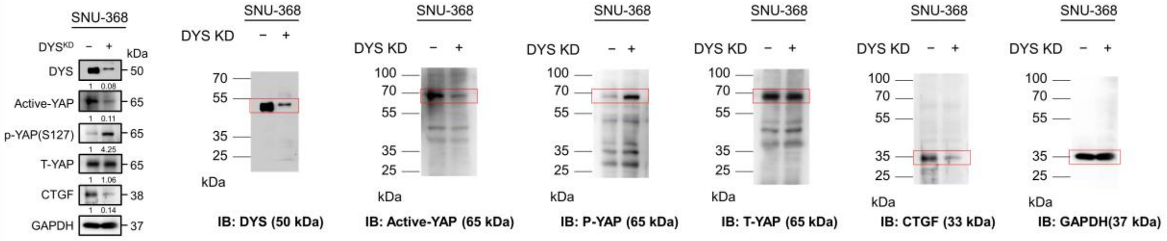

Supplementary Figure 2n

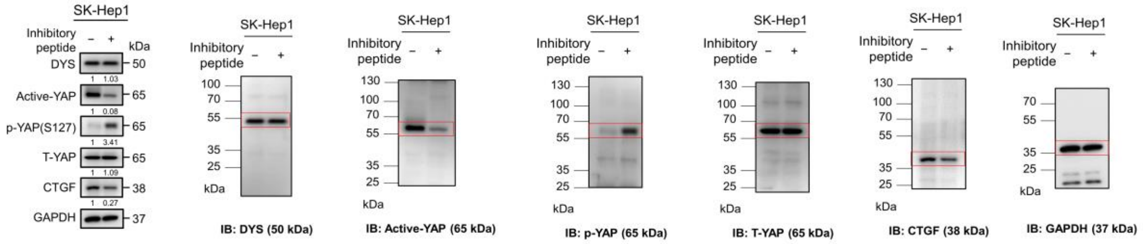

Figure 3d

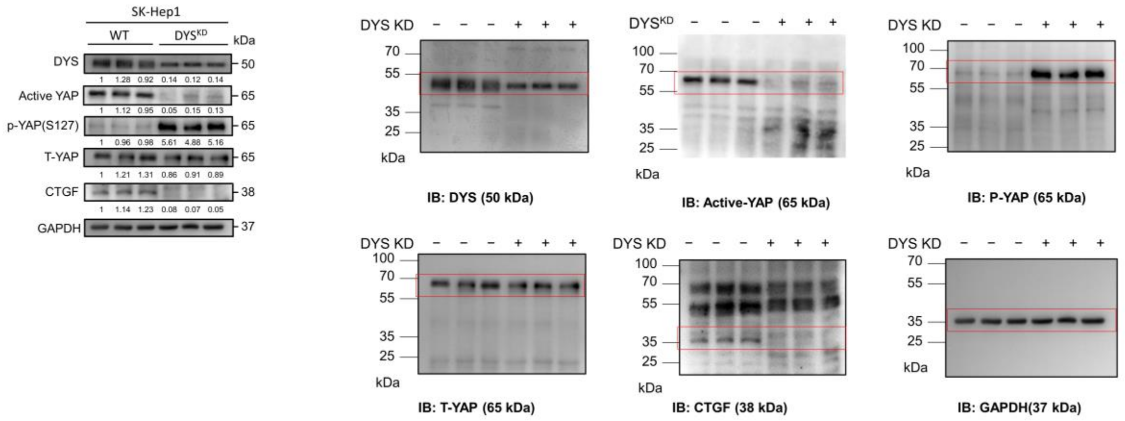

Figure 3e

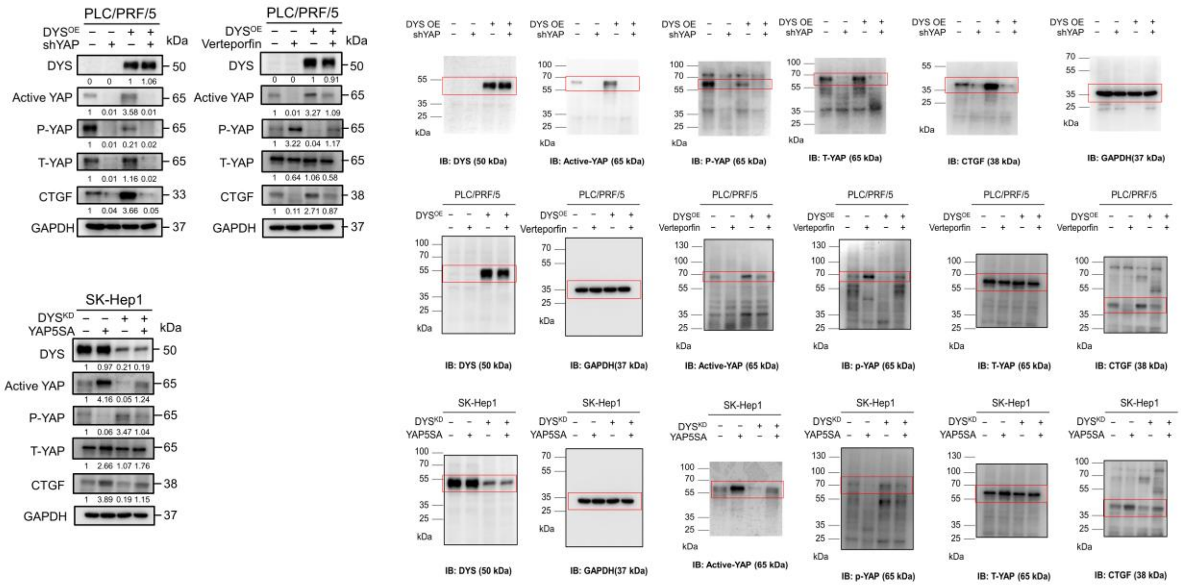

Supplementary Figure 3c

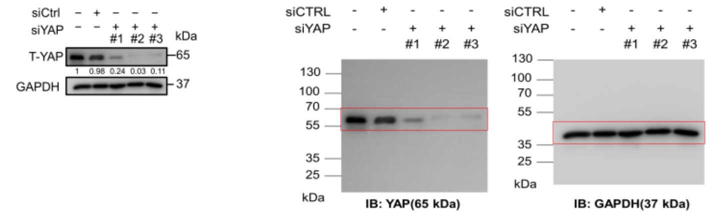

Supplementary Figure 3d

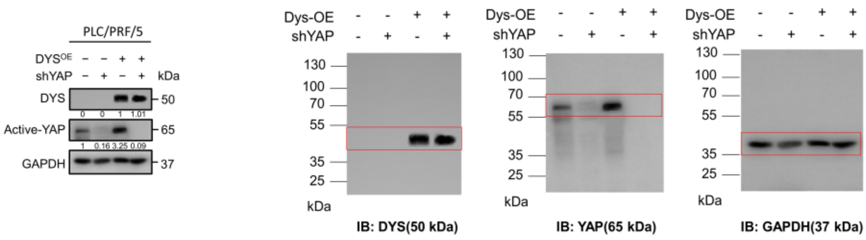

Figure 4b-1

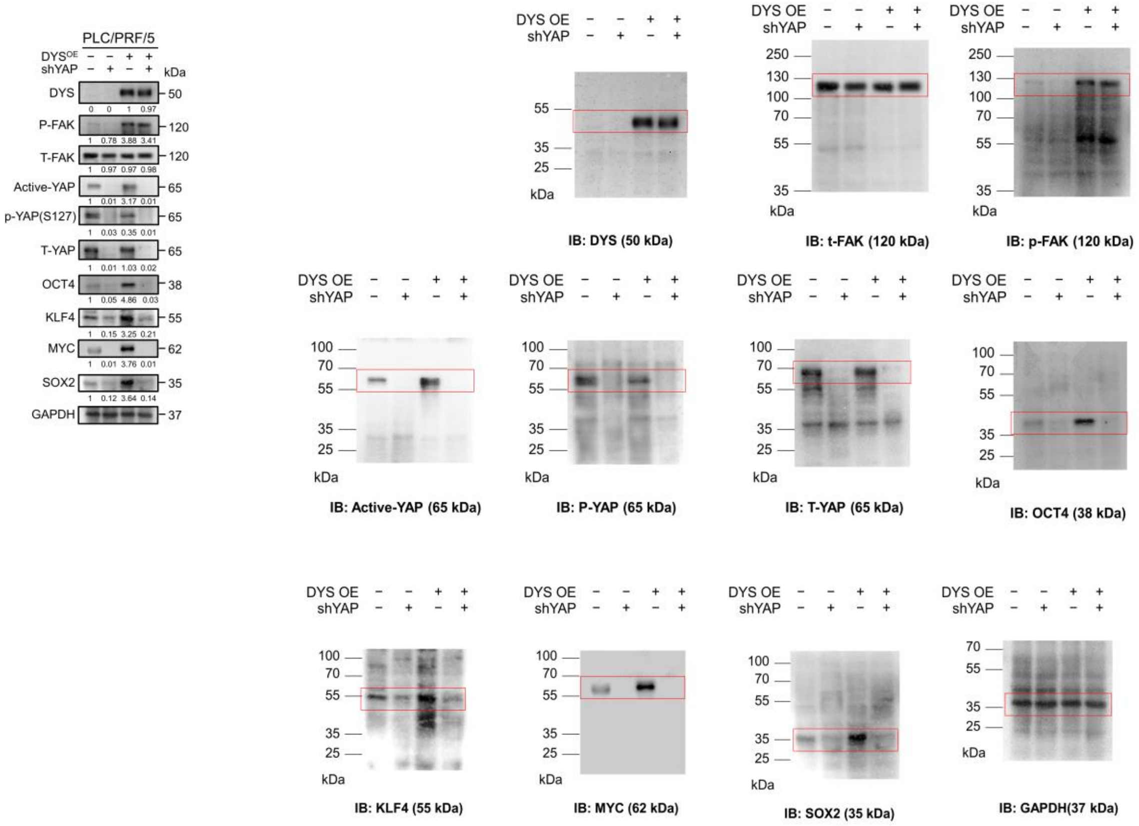
PLC/PRF/5
DYS^OE
shYAP
kDa
DYS
P-FAK
T-FAK
Active-YAP
p-YAP(S127)
T-YAP
OCT4
KLF4
MYC
SOX2
GAPDH
0 0 1 0.97
1 0.78 3.88 3.41
1 0.97 0.97 0.98
1 0.01 3.17 0.01
1 0.03 0.35 0.01
1 0.01 1.03 0.02
1 0.05 4.86 0.03
1 0.15 3.25 0.21
1 0.01 3.76 0.01
1 0.12 3.64 0.14
DYS OE
shYAP
IB: DYS (50 kDa)
IB: t-FAK (120 kDa)
IB: p-FAK (120 kDa)
IB: Active-YAP (65 kDa)
IB: P-YAP (65 kDa)
IB: T-YAP (65 kDa)
IB: OCT4 (38 kDa)
IB: KLF4 (55 kDa)
IB: MYC (62 kDa)
IB: SOX2 (35 kDa)
IB: GAPDH(37 kDa)

Figure 4b-2

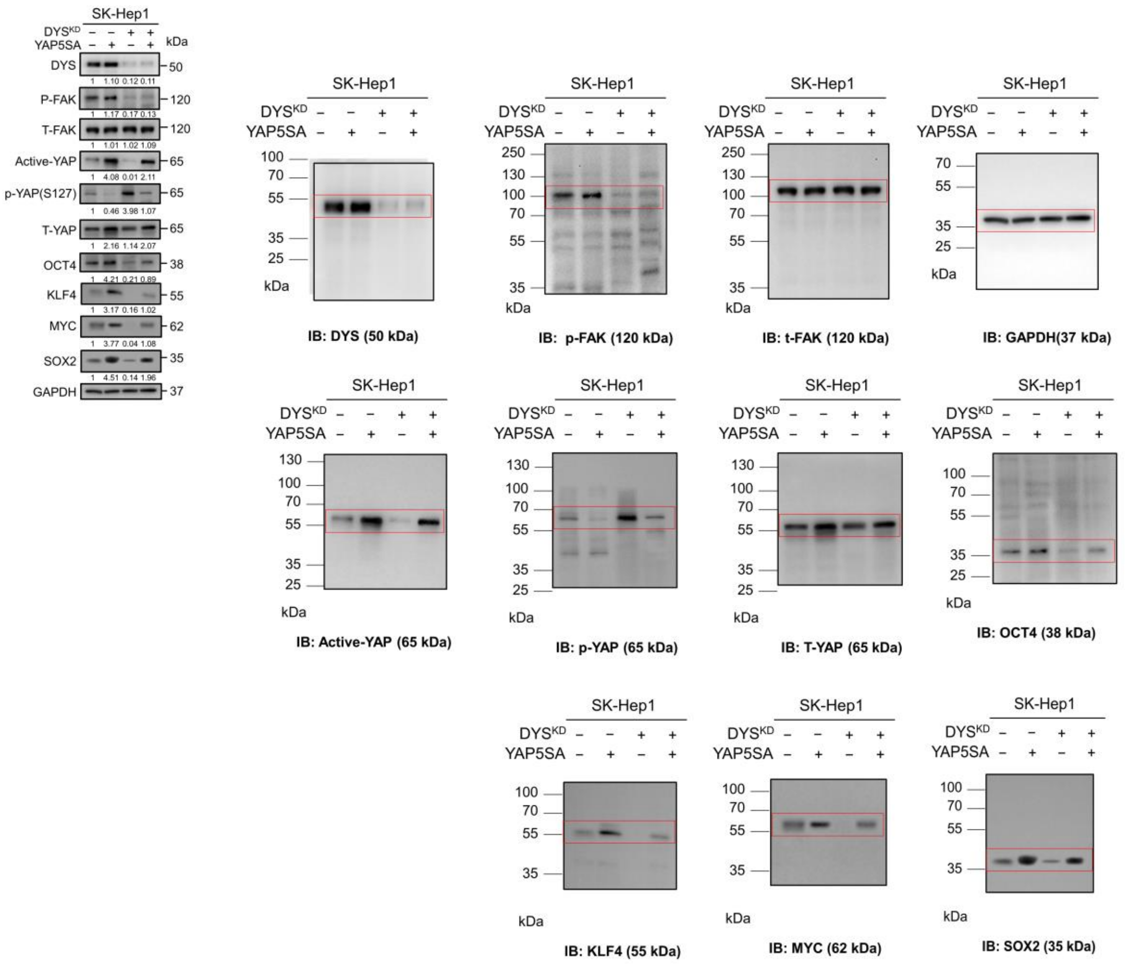
SK-Hep1
DYS^KD
YAP5SA
kDa
DYS
50
1 1.10 0.12 0.11
P-FAK
120
1 1.17 0.17 0.13
T-FAK
120
1 1.01 1.02 1.09
Active-YAP
65
1 4.08 0.01 2.11
p-YAP(S127)
65
1 0.46 3.98 1.07
T-YAP
65
1 2.16 1.14 2.07
OCT4
38
1 4.21 0.21 0.89
KLF4
55
1 3.17 0.16 1.02
MYC
62
1 3.77 0.04 1.08
SOX2
35
1 4.51 0.14 1.96
GAPDH
37
IB: DYS (50 kDa)
IB: p-FAK (120 kDa)
IB: t-FAK (120 kDa)
IB: GAPDH(37 kDa)
IB: Active-YAP (65 kDa)
IB: p-YAP (65 kDa)
IB: T-YAP (65 kDa)
IB: OCT4 (38 kDa)
IB: KLF4 (55 kDa)
IB: MYC (62 kDa)
IB: SOX2 (35 kDa)

Figure 4c

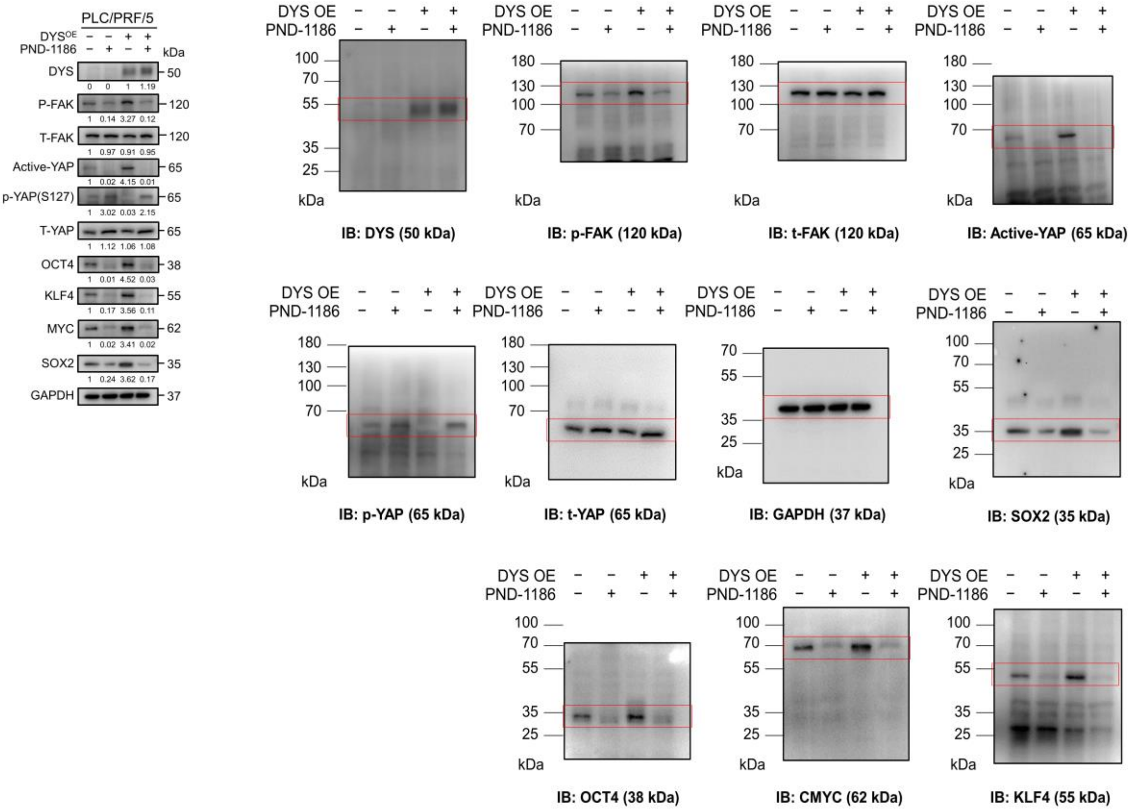

PLC/PRF/5
DYS OE
PND-1186
kDa
IB: DYS (50 kDa)
IB: p-FAK (120 kDa)
IB: t-FAK (120 kDa)
IB: Active-YAP (65 kDa)
IB: p-YAP (65 kDa)
IB: t-YAP (65 kDa)
IB: GAPDH (37 kDa)
IB: SOX2 (35 kDa)
IB: OCT4 (38 kDa)
IB: CMYC (62 kDa)
IB: KLF4 (55 kDa)

Figure 4d

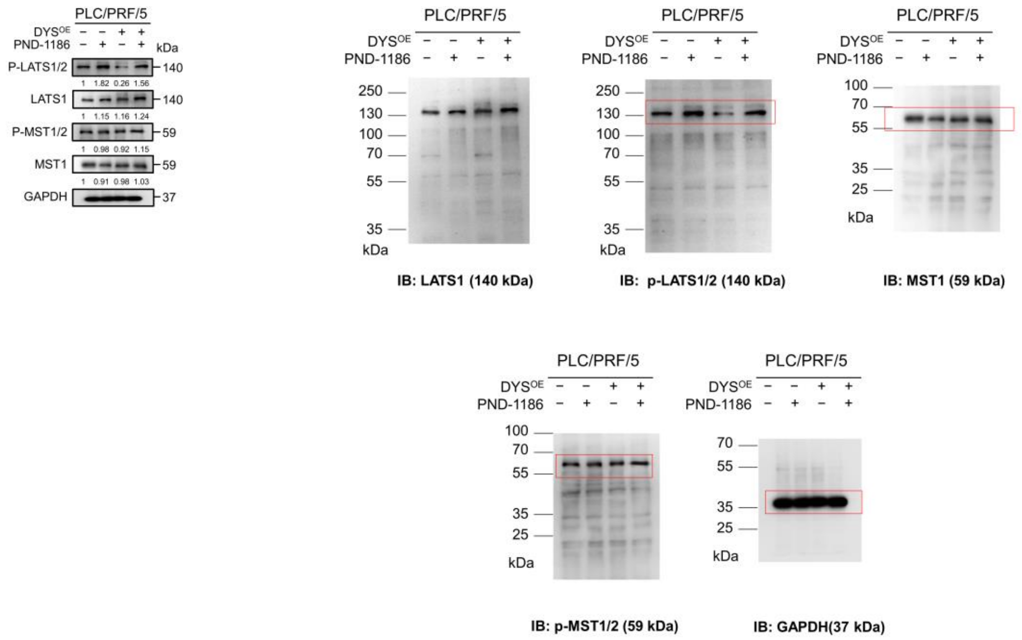

PLC/PRF/5
DYS^OE
PND-1186
kDa
P-LATS1/2
LATS1
P-MST1/2
MST1
GAPDH
140
59
37
1 1.82 0.26 1.56
1 1.15 1.16 1.24
1 0.98 0.92 1.15
1 0.91 0.98 1.03
IB: LATS1 (140 kDa)
IB: p-LATS1/2 (140 kDa)
IB: MST1 (59 kDa)
IB: p-MST1/2 (59 kDa)
IB: GAPDH(37 kDa)

Supplementary Figure 4b-1

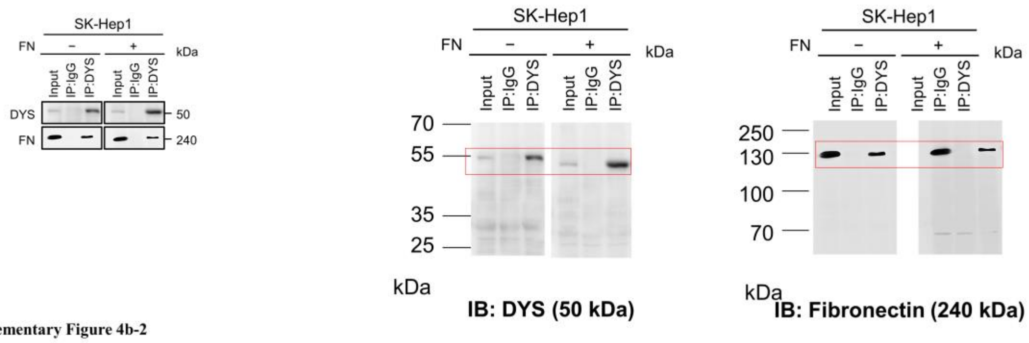

Supplementary Figure 4b-2

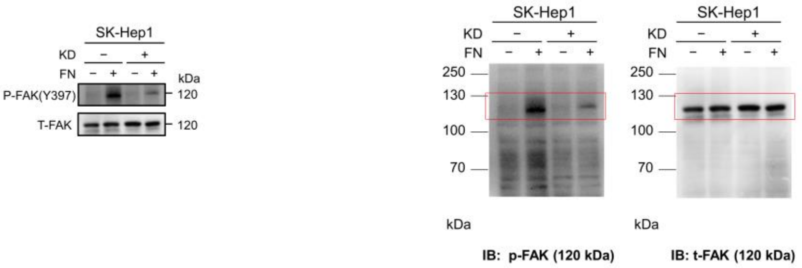

Supplementary Figure 4c-1

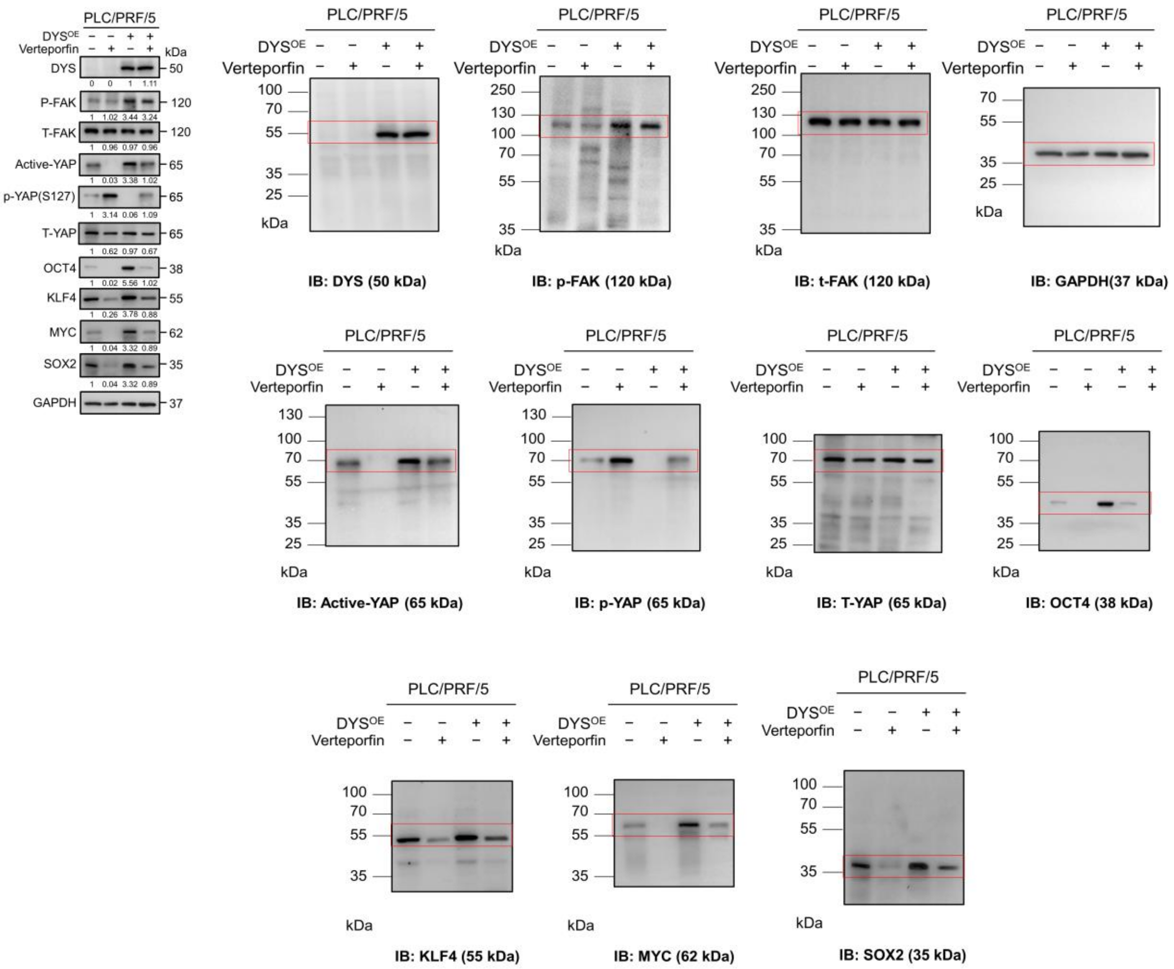

Supplementary Figure 4c-2

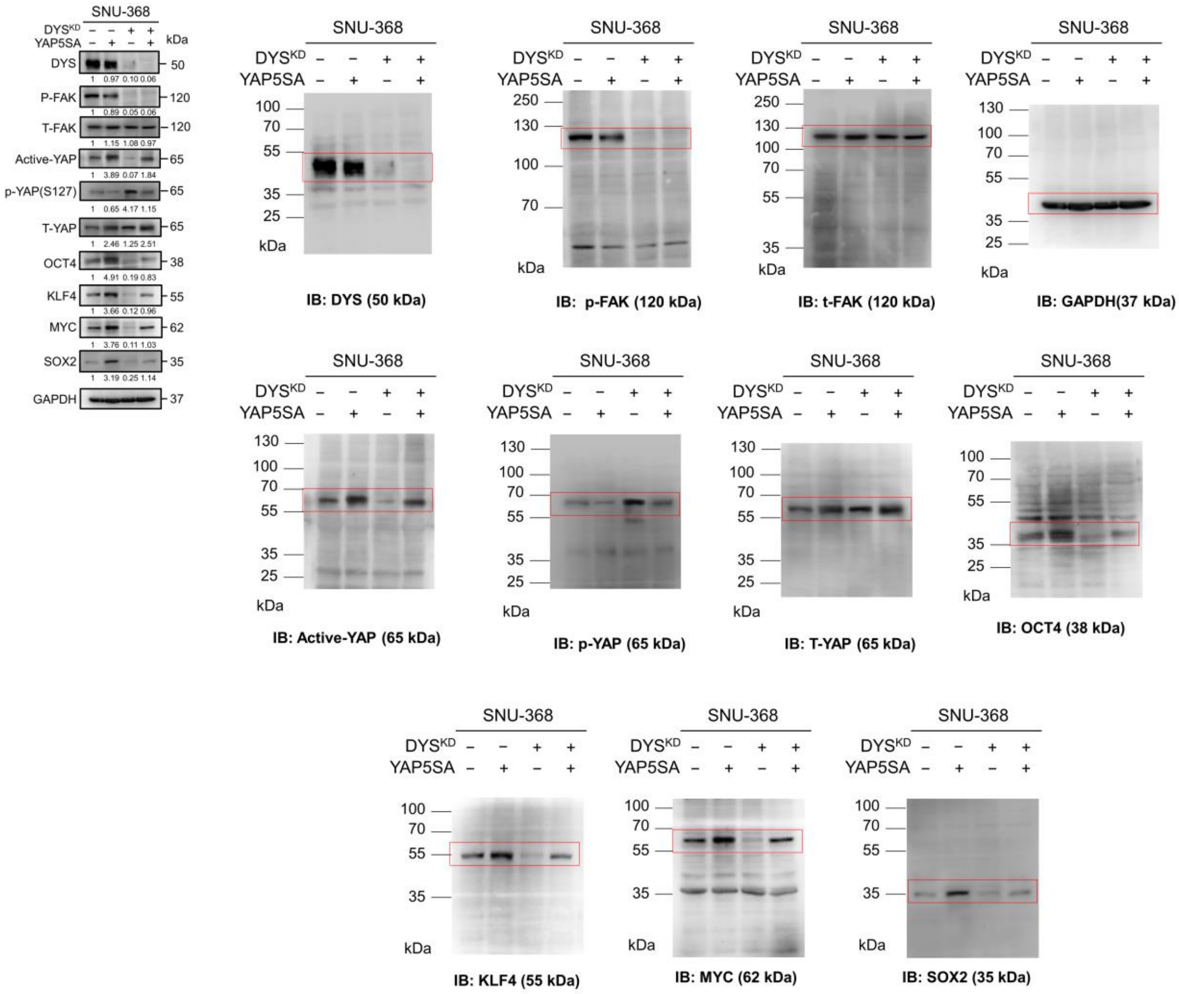

Supplementary Figure 4e

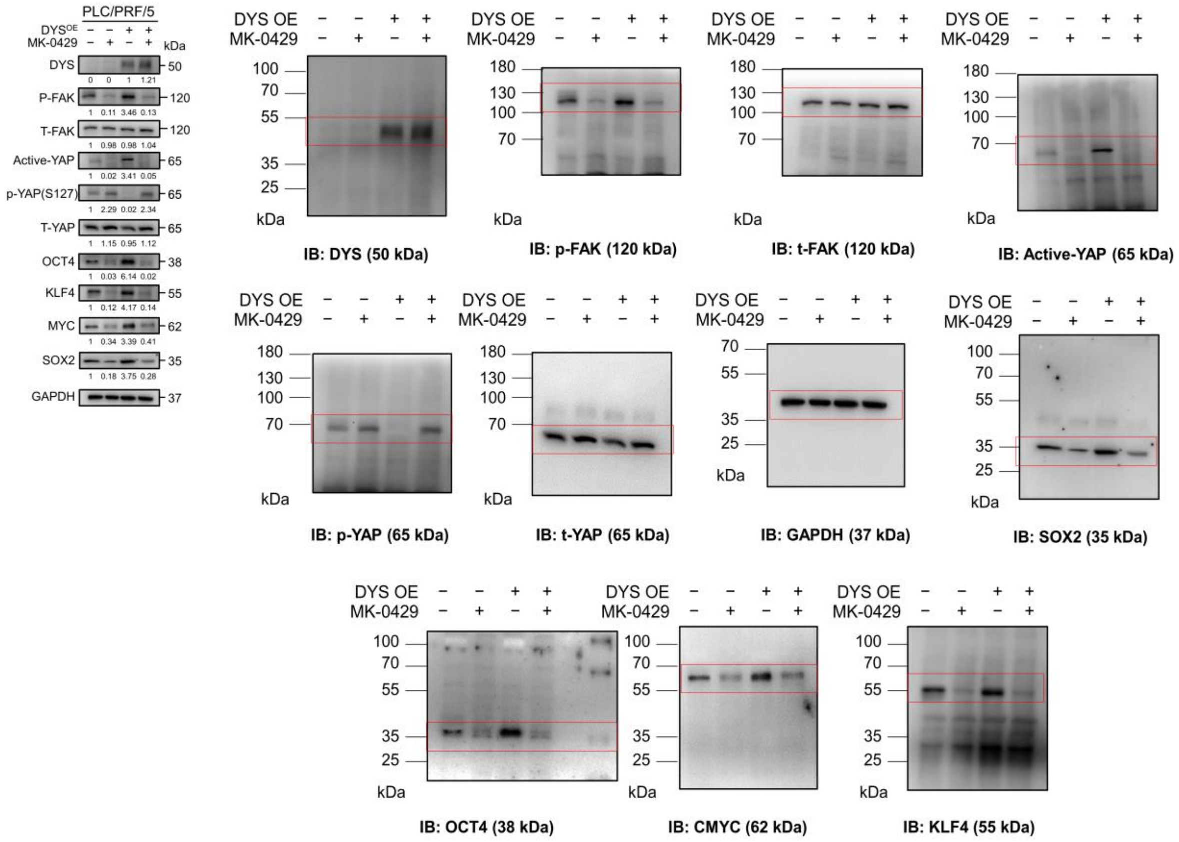

Supplementary Figure 4h

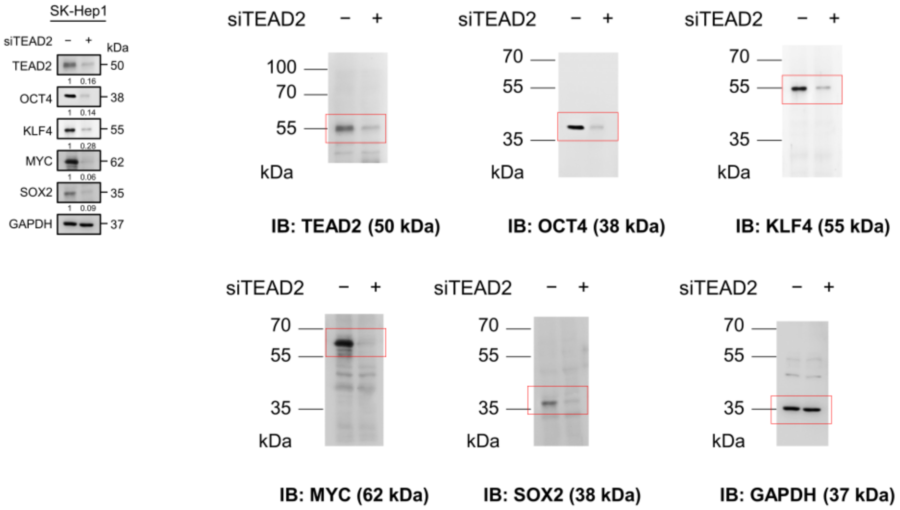

Figure 5f-1

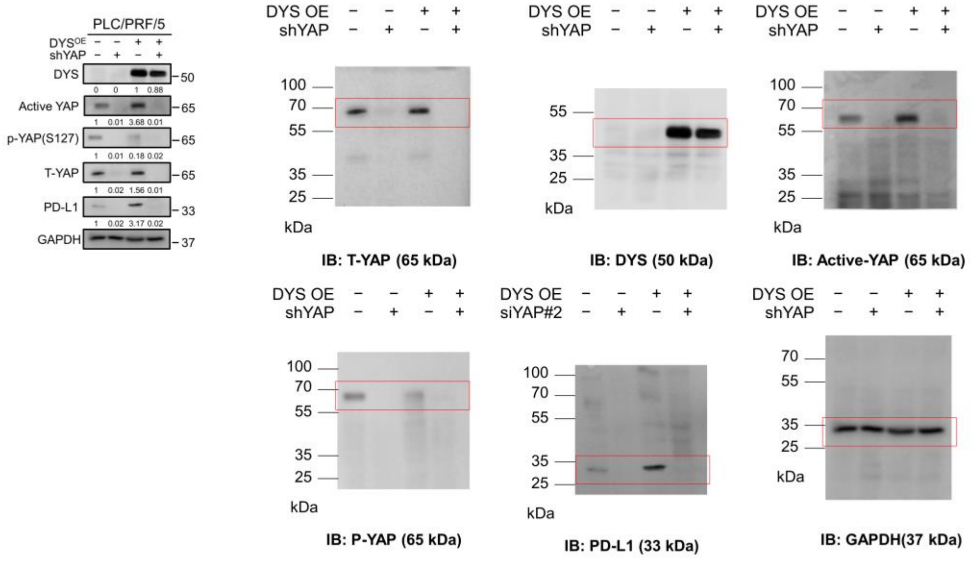

Figure 5f-2

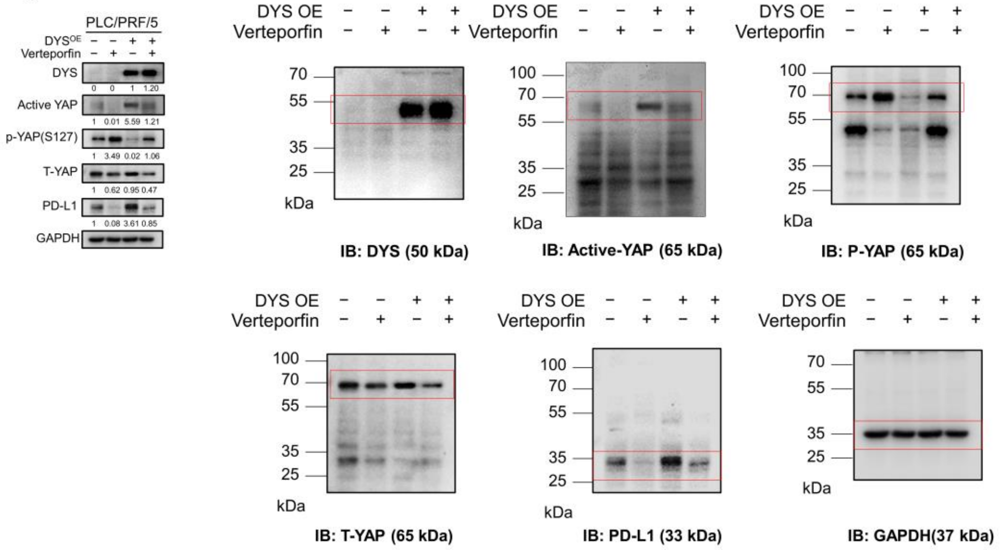

Figure 5f-3

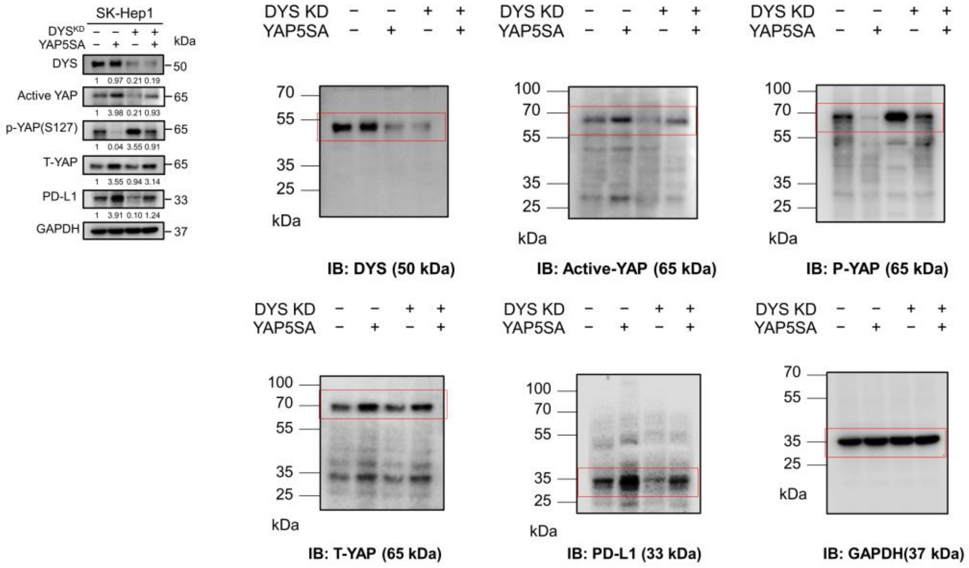
SK-Hep1
DYS^KD − − + +
YAP5SA − + − +
kDa
DYS 50
1 0.97 0.21 0.19
Active YAP 65
1 3.98 0.21 0.93
p-YAP(S127) 65
1 0.04 3.55 0.91
T-YAP 65
1 3.55 0.94 3.14
PD-L1 33
1 3.91 0.10 1.24
GAPDH 37
DYS KD − − + +
YAP5SA − + − +
IB: DYS (50 kDa)
IB: Active-YAP (65 kDa)
IB: P-YAP (65 kDa)
IB: T-YAP (65 kDa)
IB: PD-L1 (33 kDa)
IB: GAPDH(37 kDa)

Figure 6d

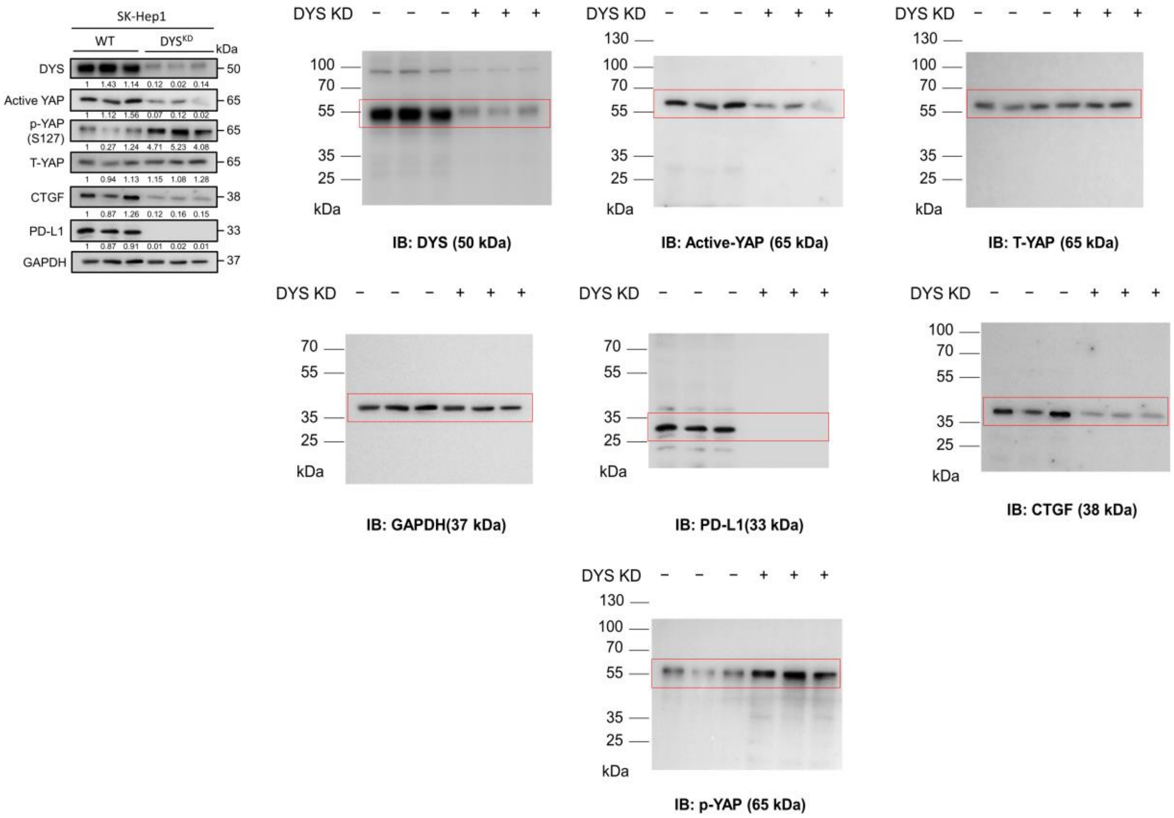
SK-Hep1
WT
DYS^KD
kDa
DYS
Active YAP
p-YAP (S127)
T-YAP
CTGF
PD-L1
GAPDH
DYS KD
IB: DYS (50 kDa)
IB: Active-YAP (65 kDa)
IB: T-YAP (65 kDa)
IB: GAPDH(37 kDa)
IB: PD-L1(33 kDa)
IB: CTGF (38 kDa)
IB: p-YAP (65 kDa)

Figure 6j

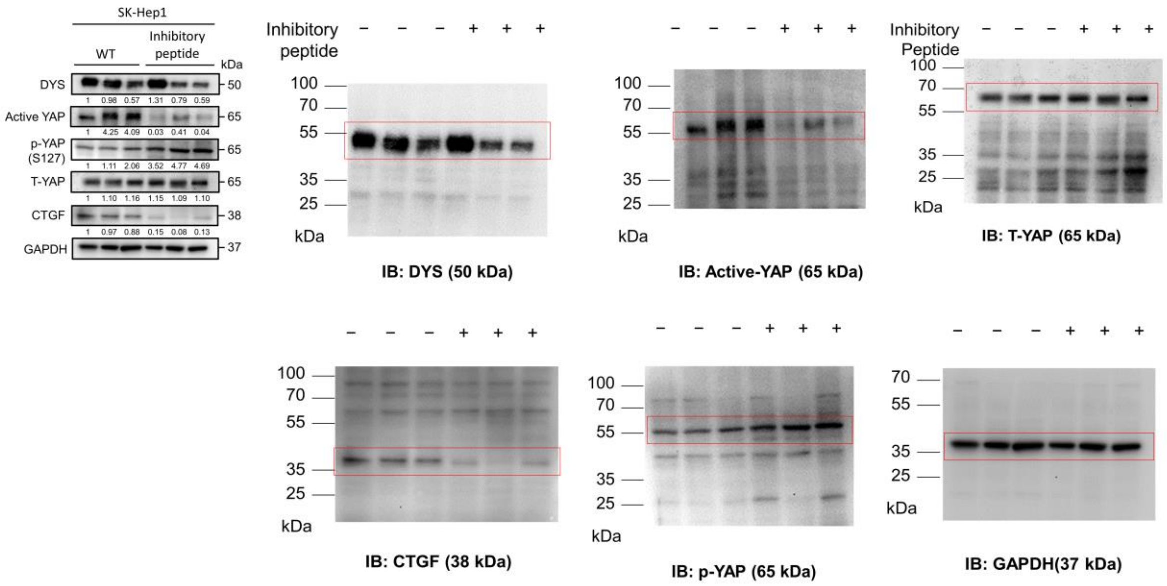
SK-Hep1
WT
Inhibitory peptide
kDa
DYS
Active YAP
p-YAP (S127)
T-YAP
CTGF
GAPDH
1 0.98 0.57 1.31 0.79 0.59
1 4.25 4.09 0.03 0.41 0.04
1 1.11 2.06 3.52 4.77 4.69
1 1.10 1.16 1.15 1.09 1.10
1 0.97 0.88 0.15 0.08 0.13
50
65
65
65
38
37
Inhibitory peptide
Inhibitory Peptide
100
70
55
35
25
kDa
IB: DYS (50 kDa)
IB: Active-YAP (65 kDa)
IB: T-YAP (65 kDa)
IB: CTGF (38 kDa)
IB: p-YAP (65 kDa)
IB: GAPDH(37 kDa)

Supplementary Figure 7h

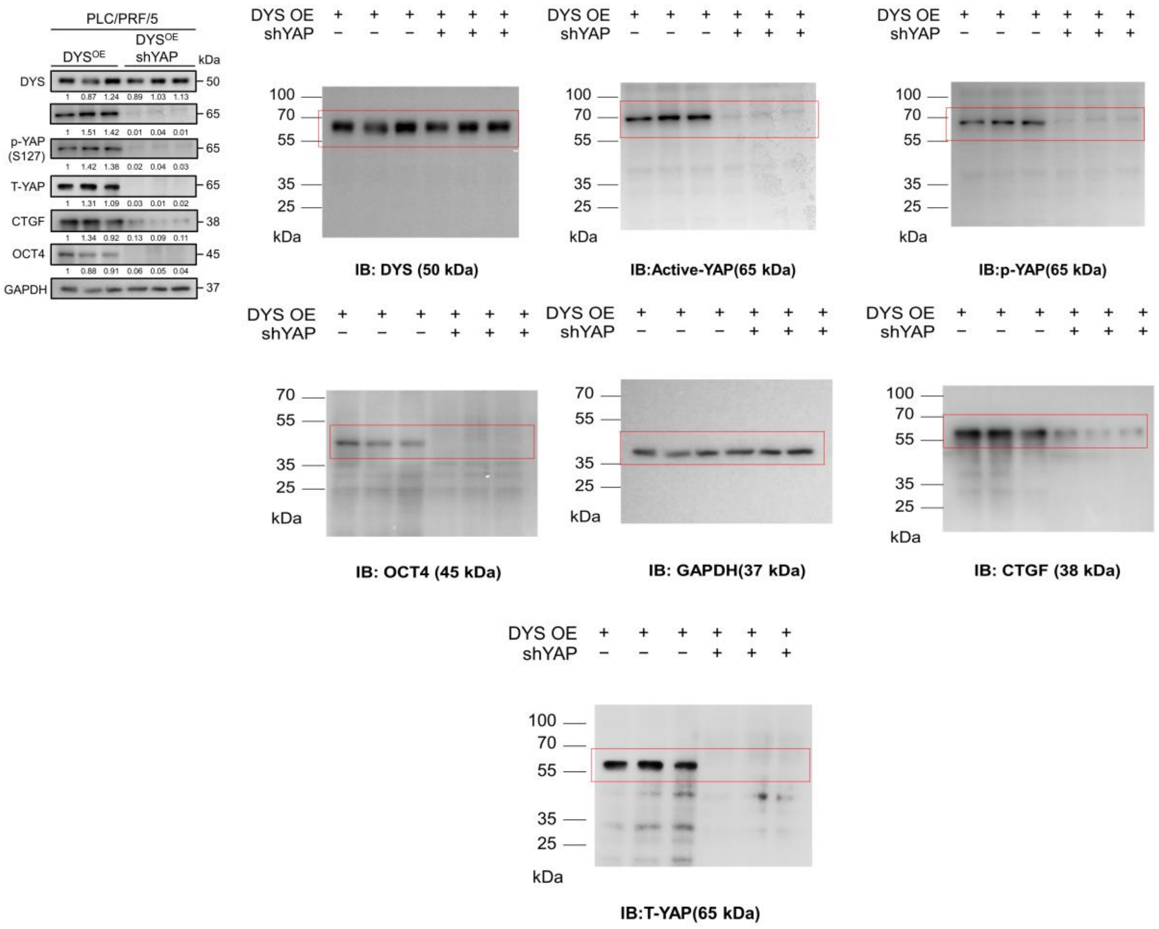

Supplement: Supplementary file 3 — Supplementary Information [file 41392_2025_2520_MOESM3_ESM.pdf]
